# Adaptive introgression from distant Caribbean islands contributed to the diversification of a microendemic adaptive radiation of trophic specialist pupfishes

**DOI:** 10.1371/journal.pgen.1006919

**Published:** 2017-08-10

**Authors:** Emilie J. Richards, Christopher H. Martin

**Affiliations:** Biology Department, University of North Carolina at Chapel Hill, Chapel Hill, North Carolina, United States of America; Harvard University, UNITED STATES

## Abstract

Rapid diversification often involves complex histories of gene flow that leave variable and conflicting signatures of evolutionary relatedness across the genome. Identifying the extent and source of variation in these evolutionary relationships can provide insight into the evolutionary mechanisms involved in rapid radiations. Here we compare the discordant evolutionary relationships associated with species phenotypes across 42 whole genomes from a sympatric adaptive radiation of *Cyprinodon* pupfishes endemic to San Salvador Island, Bahamas and several outgroup pupfish species in order to understand the rarity of these trophic specialists within the larger radiation of *Cyprinodon*. 82% of the genome depicts close evolutionary relationships among the San Salvador Island species reflecting their geographic proximity, but the vast majority of variants fixed between specialist species lie in regions with discordant topologies. Top candidate adaptive introgression regions include signatures of selective sweeps and adaptive introgression of genetic variation from a single population in the northwestern Bahamas into each of the specialist species. Hard selective sweeps of genetic variation on San Salvador Island contributed 5 times more to speciation of trophic specialists than adaptive introgression of Caribbean genetic variation; however, four of the 11 introgressed regions came from a single distant island and were associated with the primary axis of oral jaw divergence within the radiation. For example, standing variation in a proto-oncogene (*ski*) known to have effects on jaw size introgressed into one San Salvador Island specialist from an island 300 km away approximately 10 kya. The complex emerging picture of the origins of adaptive radiation on San Salvador Island indicates that multiple sources of genetic variation contributed to the adaptive phenotypes of novel trophic specialists on the island. Our findings suggest that a suite of factors, including rare adaptive introgression, may be necessary for adaptive radiation in addition to ecological opportunity.

## Author summary

Groups of closely related species can rapidly evolve to occupy diverse ecological roles, but the ecological and genetic conditions that trigger this diversification are still highly debated. We examined patterns of molecular evolution across the genomes of a recent radiation of pupfishes that includes two trophic specialists. Despite apparently widespread ecological opportunities and gene flow across the Caribbean, this radiation is endemic to a single Bahamian island. Using the whole genomes of 42 pupfish we found evidence of extensive and previously unexpected variation in evolutionary relatedness among Caribbean pupfish. While adaptive introgression appears to be rare across the genomes of the San Salvador Island species, it may have introduced adaptive variants important in the evolution of the complex phenotypes of the specialists. Four of the 11 candidate adaptive introgression regions contain genes with known effects on jaw morphology in zebrafish or associated with pupfish jaw size, the primary axis of phenotypic divergence between species in this system. Our findings that multiple sources of genetic variation contribute to the San Salvador Island radiation suggests that a complex suite of factors, including hybridization with other species, may be necessary for adaptive radiation in addition to ecological opportunity.

## Introduction

Adaptive radiations are central to our understanding of evolution because they generate a wealth of ecological, phenotypic, and species diversity in rapid bursts. However, the mechanisms that trigger rapid bursts of trait divergence, niche evolution, and diversification characteristic of classic adaptive radiations are still debated. The availability of resources in new environments with few competitors has long been seen as the major force driving adaptive radiations [1–3], but it is a longstanding question why only some lineages rapidly diversify in response to such ecological opportunities while others do not [4–9].

While gene flow can impede or reverse diversification among incipient species by reducing genetic differentiation and subsequent recombination can break down locally adapted haplotypes [10–13], it can also introduce adaptive genetic variants [14,15] and/or genetic incompatibilities [16–18] that initiate or contribute to the process of speciation. A growing number of studies have identified gene flow and genome-wide introgression across a range of adaptive radiations [19–26], contributing to the emerging view that gene flow is pervasive throughout the history of many young rapidly diversifying groups and may be necessary for adaptive radiation. Examples of adaptive radiations with histories of extensive hybridization include *Heliconius* butterflies [27–29], Darwin’s finches [21,30–32], *Anopheles* mosquitos [20,33], and cichlids [24,25,34–39]. The hybrid swarm hypothesis [40] proposes that hybridization among distinct lineages can introduce genetic diversity and novel allele combinations genome-wide that may trigger rapid diversification in the presence of abundant ecological opportunity. However, it is still unclear how often hybridization is necessary for rapid diversification, as opposed to simply being pervasive throughout the history of any young rapidly diversifying group [25,41]. One of the only examples with strong evidence of hybridization leading to ecological and species diversification is that of several hybrid species within a radiation of *Helianthus* sunflowers [42–47]. However, these may simply represent examples of multiple homoploid speciation events within an already radiating lineage rather than a hybrid swarm scenario. So while there is convincing evidence that hybridization can facilitate diversification among species pairs (but see [26,38] for a potential multispecies outcome of hybridization), it is still unclear whether gene flow is a major factor constraining adaptive radiation in some lineages or if ecological opportunity is the sole constraint.

The adaptive radiation of San Salvador Island pupfishes provides an outstanding system to compare the contributions of different sources of genetic variation to rapid diversification and the role of gene flow in the evolution of complex phenotypes. Pupfish species of the genus *Cyprinodon* inhabit saline lakes and coastal areas across the Caribbean and Atlantic and nearly all pupfishes are allopatric, dietary generalists consuming algae and small invertebrates [48]. In contrast, three *Cyprinodon* species live sympatrically in the hypersaline lakes of San Salvador Island and comprise a small radiation that has occurred within the past 10,000 years based on the most recent glacial maximum when these lakes were dry due to lowered sea levels [49–51]. This radiation is composed of the widespread generalist algae-eating species *Cyprinodon variegatus* and two endemic specialists that coexist with the generalist in all habitats in some lakes. These specialists have adapted to unique trophic niches using novel morphologies: the molluscivore *Cyprinodon brontotheroides* with a unique nasal protrusion and the scale-eater *Cyprinodon desquamator* with enlarged oral jaws and adductor mandibulae muscles [48,52]. Surveys of populations living on neighboring islands in the Bahamas and phylogenetic analyses with other *Cyprinodon* species indicate that these specialist species are endemic to the hypersaline lakes of San Salvador Island and that both specialists arose from a generalist common ancestor during this recent radiation [53].

The currently available ecological and genetic data on the group provides little indication as to why this radiation is localized to a single island. Variation in ecological opportunity among hypersaline lake environments in the Caribbean does not appear to explain the rarity of this radiation [53]. This finding suggests a potentially important role for sufficient genetic variation to respond to abundant, underutilized resources in these environments. However, a hybrid swarm hypothesis about the origins of the radiation does not appear to explain its rarity either: genetic diversity is comparable among islands and gene flow occurs among all Caribbean islands investigated, not only into San Salvador Island [53]. Novel traits and increased rates of diversification associated with them are well documented in this system [48,53,54], but understanding the rarity of this adaptive radiation requires a thorough investigation of the underlying genetic variation that accompanies these rare ecological transitions. A recent study investigating the genetic basis of trophic specialists in this radiation revealed very few regions underlying these phenotypes [55]. Only thousands of variants out of 12 million were fixed between the scale-eater and molluscivore species. Since genetic divergence is limited to particular regions, localized rather than genome-wide investigations of the genome will be important for understanding how genetic variation, possibly originating outside of San Salvador Island, has contributed to the exceptional phenotypic diversification restricted to this island. Here, we use a machine-learning approach to identify regions of the genome with different evolutionary relationships among 42 pupfish genomes sampled from the San Salvador Island radiation, two distant Caribbean islands, and 3 additional outgroups. We then scan the genome for evidence of localized introgression with pupfish populations outside of San Salvador Island and compare the relative contributions of adaptive introgression from two distant islands and hard selective sweeps to the divergence of each specialist species.

## Results

### Extensive variation in patterns of evolutionary relatedness across the genome

To identify localized patterns of population history across the genome, we used the machine-learning approach SAGUARO. SAGUARO combines a hidden Markov model with a self-organizing map to characterize local topologies across the genome among aligned individuals [56]. This method does not require any *a priori* hypotheses about the relationships among individuals, but rather infers them directly from the genome by finding regions of consecutive nucleotides with a similar pattern of genetic differentiation, building hypotheses about relationships among individuals from these genetic differences, and then assigning regions of the genome to these hypothesized local topologies. Since smaller segments with fewer informative SNPs are more likely to be incorrectly assigned to a hypothesized topology by chance (pers. comm. M.G. Grabherr), we tested various minimum SNP filters for reducing the amount of short, uninformative segments assigned to topologies by chance and found that increasingly stringent filters over 20 SNPs did not substantially reduce the number of uninformative segments. Using this approach and our 20 SNP filter, we partitioned the genome into a total of 15 unique topologies across 227,248 genomic segments that ranged from 101–324,088 base pairs in length (median: 852 bp) (S1 and S2 Figs; S1 Table). The 15^th^ topology was uninformative about either species or population level relationships, so it was removed from downstream analyses.

**Fig 1:**
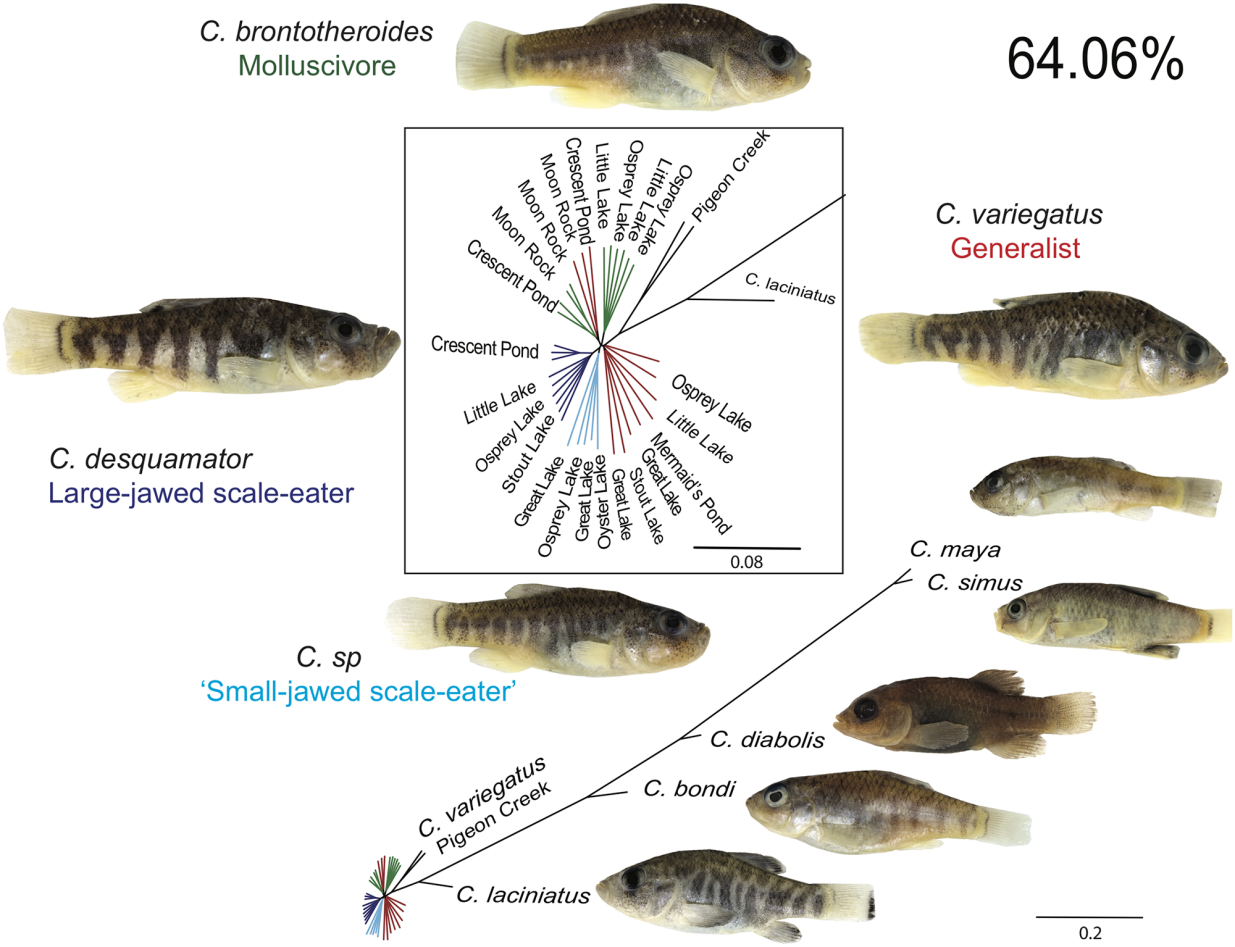
The most common topology estimated in SAGUARO showing a monophyletic San Salvador Island clade covered 64% of the genome. San Salvador Island generalists (red), molluscivores (green), large-jawed scale-eaters (dark blue), small-jawed scale-eaters (light blue), and outgroup species (black) in the Caribbean, California, and Mexico. Other topologies featuring a monophyletic San Salvador Island clade are presented in S1 Fig.

The most prevalent history across 64% of the genome featured the expected species phylogeny for this group from previous genome-wide studies [48,53,57], in which all individuals from San Salvador Island grouped by species into a single clade with distant relationships to outgroup generalist pupfish populations from other islands in the Caribbean, Death Valley in California, and a second radiation in Mexico spanning the most divergent branch of the *Cyprinodon* tree (Fig 1). Unlike previous genome-wide phylogenies [53,57], and with the exception of a few individuals that grouped with molluscivores by lake, the generalists on San Salvador Island form a discrete clade from the molluscivores and scale-eaters.

Within this dominant topology, scale-eater*s* from six lakes on San Salvador Island fell into one of two separate clades: small-jawed individuals from Osprey Lake, Great Lake, and Oyster Pond and large-jawed individuals from Crescent Pond, Stout Lake, Osprey Lake, and Little Lake (Fig 1). Molluscivores did not form a single clade as individuals from some lakes (Crescent Pond and Moon Rock) were more closely related to generalists from the same lake than molluscivores from other lakes, similar to previous genome-wide phylogenies [57]. Another topology covering 10% of the genome was very similar to the dominant one, differing only in the relationships among San Salvador Island generalists (S1 Fig). Additional topologies spanning 7.6% of the genome featured a single San Salvador Island clade but also depicted a closer relationship between San Salvador Island and the outgroups as well as groupings of all three San Salvador Island species by lake in Crescent Pond and Moon Rock Pond. When combined with the dominant topology, only 82.6% of the genome supported the expected San Salvador Island clade (S1 Table).

**Fig 2:**
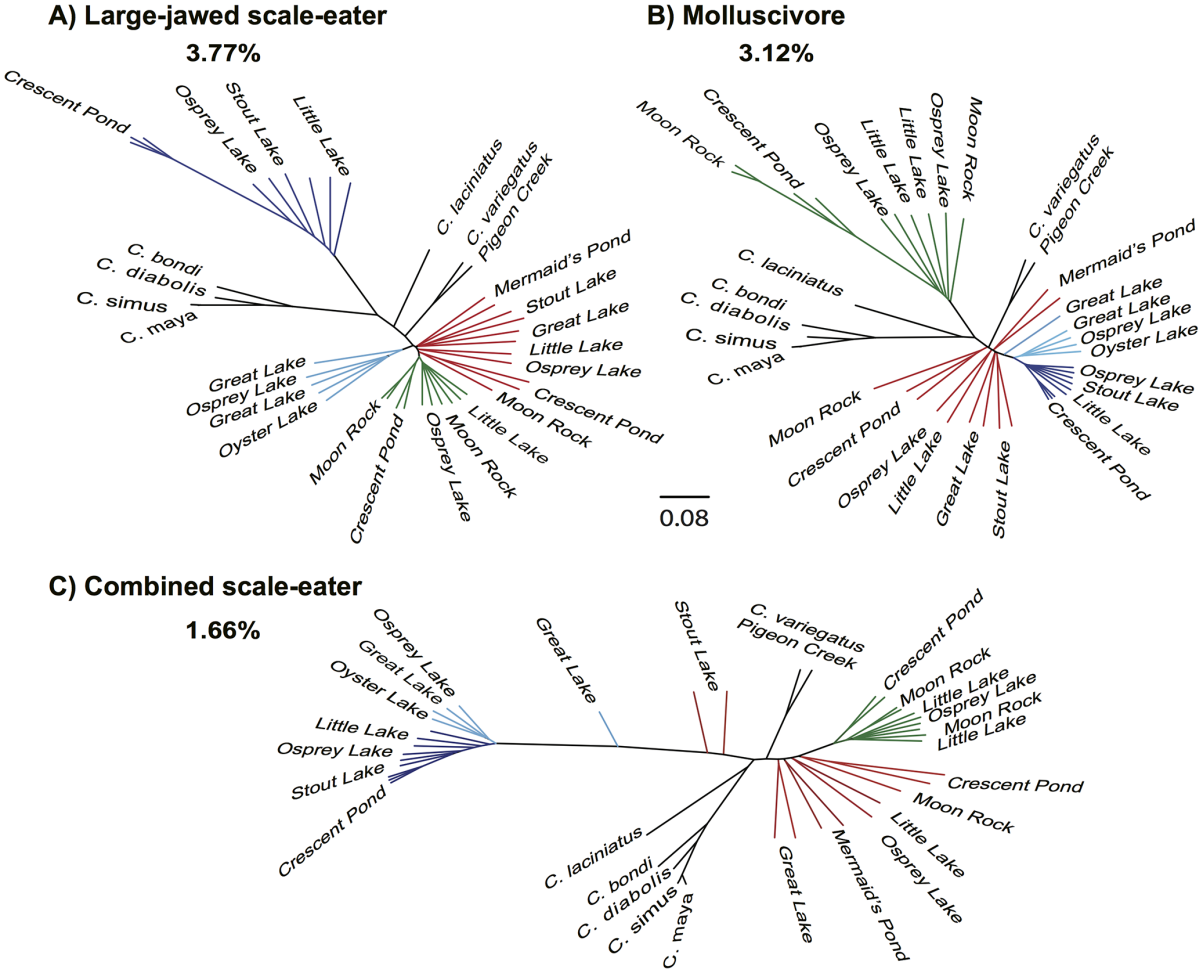
Alternative topologies estimated in SAGUARO showing San Salvador Island specialists grouping with outgroups. (A) The large-jawed scale-eater topology covered 3.77% of the genome, in which large-jawed scale-eater individuals showed a sister relationship to outgroup pupfish species. (B) The molluscivore topology covered a non-overlapping 3.12% of the genome, in which molluscivores showed a sister relationship to outgroup pupfish species. (C) The combined scale-eater topology covered a non-overlapping 1.66% of the genome, where all scale-eaters (along with two generalists from Stout’s Lake) showed a sister relationship to the outgroup pupfish species. Additional alternative topologies are presented in S2 Fig.

In other regions of the genome, San Salvador Island did not form a single clade (Fig 2A–2C and S2 Fig, S1 Table). The most frequently observed alternative relationships depicted specialist individuals as a clade outside of the San Salvador Island group and sister to all the outgroup *Cyprinodon* species (Fig 2A and 2B). The ‘large-jawed scale-eater topology’ featured large-jawed scale-eaters outside of the San Salvador Island clade, sister to all other outgroups, and was assigned to 4,437 segments covering 3.77% of the genome (Fig 2A). Another topology, the ‘molluscivore topology’, showed a similar pattern in which the molluscivores formed a single clade outside of the San Salvador Island group and sister to all other outgroups (Fig 2B). This molluscivore topology was assigned to 3,916 segments and covered 3.12% of the genome. Another 2,029 segments covering 1.66% of the genome were assigned to a topology where both the large-jawed and small-jawed scale-eaters formed a combined clade outside of the San Salvador Island group, the ‘combined scale-eater topology’ (Fig 2C). Other topologies featuring one of the specialists separated from the rest of San Salvador Island covered 0.76%-2.48% of the genome (S1 Table).

Unexpectedly, all 14 informative topologies separated scale-eaters into groups of small- and large-jawed individuals and the relationships between these two groups and other species differed across different regions of the genome. In some regions, the small-jawed scale-eater individuals were sister to the large-jawed scale-eaters (Figs 1, 2B and 2C, S1 and S2 Figs). In other regions, the small-jawed scale-eaters were more closely related to the generalists and molluscivores (Fig 2A, S1 and S2 Figs). These small-jawed scale-eaters may be a product of ongoing hybridization between species on San Salvador Island or a new ‘occasional’ scale-eating ecomorph, perhaps representing an intermediate yet viable stage on the evolutionary path towards large-jawed scale-eaters, in which scales form the majority of their diet [54]. The presence of homozygous genotypes in all five individuals of small-jawed scale-eaters for variants fixed in both large-jawed scale-eaters and generalists is not consistent with first generation hybrids (S2 Table). They also do not fit the ancestry proportions expected in F_2_ hybrids (*χ*^2^ = 429.6, *P* = 5.16e^-94^). We might expect increased linkage disequilibrium (LD) in the small-jawed scale-eaters if they represent recent hybridization events between distinct populations. Consistent with this idea, LD decays more slowly in the small-jawed scale-eaters (after approximately 120 kb) than in the three San Salvador Island species (after approximately 50kb: S3 Fig). However, strong LD and long haplotype blocks may also result from other evolutionary phenomena like recent population bottlenecks (e.g. [58]). Demographic modeling with a larger sample will be needed to distinguish whether these small-jawed scale-eaters represent hybrids from ongoing or recent gene flow on San Salvador Island or a potential new ecomorph.

### Localized introgression into both specialists from across the Caribbean

We examined signals of introgression from two distant pupfish generalist populations in the Caribbean: Lake Cunningham, New Providence Island in the Bahamas (described as the endemic species *Cyprinodon laciniatus* [59]) and Etang Saumatre / Lac Azuei in the Dominican Republic (described as the endemic species *Cyprinodon bondi* [60]). *C*. *laciniatus* exhibits morphological variation not observed in other generalist species, including laciniated scales and variation in oral jaw size [59], although not the extreme oral jaw morphologies observed in the specialists, and is an interesting candidate for looking at adaptive introgression of variants involved in oral jaw size morphology on San Salvador Island. *C*. *bondi* is a generalist species of the *variegatus* complex from the south-eastern end of the range of Greater Antillean pupfish and introgression with San Salvador Island populations would suggest that Caribbean-wide gene flow may have contributed to the adaptive radiation on San Salvador Island. We characterized the genomic landscape of introgression in the three San Salvador Island species using *f*_*4*_ statistics that were initially developed to test for introgression among human populations [61–63].

Genome-wide *f*_*4*_ tests provided evidence of introgression between Caribbean outgroups and San Salvador Island. *f*_*4*_ values significantly deviated from the null hypothesis of no introgression (*f*_*4*_ = 0) in the scale-eater/molluscivore (Z = 4.2, *P* = 2.67x10^-5^), and scale-eater/generalist combinations (Z = 4.67, *P* = 3.01x10^-6^), but were not significant in the molluscivore/generalist combination (Z = -1.63, *P* = 0.103).

**Fig 3:**
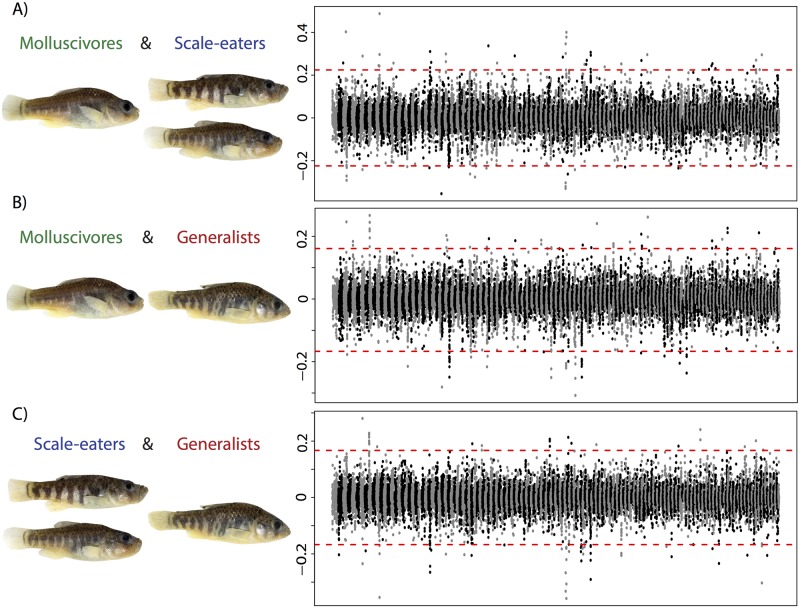
Variable introgression from distant Caribbean islands across the genomes of the San Salvador Island trophic specialists. Manhattan plot of the *f*_*4*_ values between the *C*. *laciniatus* from New Providence Island, Bahamas, *C*. *bondi* from Etang Saumatre, Dominican Republic and (A) molluscivores and scale-eaters on San Salvador Island, (B) molluscivores and generalists from San Salvador Island, (C) scale-eaters and generalists on San Salvador Island. Alternating gray/black colors indicate different scaffolds from the largest 170 scaffolds of the genome. Dotted red lines mark the permutation based significance threshold. Full Manhattan plots for each comparison are presented in S3–S5 Figs.

When *f*_*4*_ was calculated in windows, we found that 181 10-kb regions out of 100,260 (0.18%) contained significant evidence of introgression between *C*. *laciniatus* or *C*. *bondi* and the San Salvador Island specialists (Fig 3A). Introgressed regions were scattered across the genome in 107 of the 9,259 scaffolds in our dataset. These regions were not typically concentrated in one section of the genome, with the largest cluster within a single scaffold containing 12% of the total (Fig 3A).

The genomic regions with significant evidence of introgression varied between the two specialists (Fig 3B and 3C): only 15 regions from the 176 and 112 regions with significant evidence of introgression were shared between generalist/scale-eater and generalist/molluscivore comparisons, respectively. This suggests that admixture with other Caribbean populations occurred multiple times and independently for each specialist or that different introgressed regions were used by the two specialists after a single admixture event (see S4–S6 Figs for full Manhattan plots).

We also tested for introgression with the small-jawed scale-eaters excluded to search for potential introgression with the large-jawed scale-eaters alone (S7 Fig). Introgressed regions were less variable between the two groups of scale-eaters, with 122 of 209 candidate introgressed regions shared. The 87 introgressed regions unique to the large-jawed scale-eaters suggest that some introgression may have occurred between populations on other Caribbean islands and the large-jawed scale-eater population independently from the small-jawed scale-eaters.

Regions of low diversity and low recombination may be biased when genome-wide tests of introgression, such as the *f*_*4*_ statistic, are applied to genomic windows [64]. To assess whether our introgressed regions were the result of this bias, we looked at π estimates across the detected regions of introgression in comparison to the genome-wide estimates (mean D_xy_ = *0*.*007*; mean π scale-eater = *0*.*0048*; mean π molluscivore = *0*.*0054*) and variance in *f*_*4*_ statistic values. *f*_*4*_ statistics do appear slightly sensitive to the level of diversity in a region, with variance in *f*_*4*_ values having a weak negative correlation with mean scaffold π (Pearson’s *r* = -0.18; S8 Fig), and a weaker correlation between the value of *f*_*4*_ and π (Pearson’s *r* = -0.013; S9 Fig). However, in selecting our top candidate introgressed regions, we assessed π in all three San Salvador Island species and looked for other signals of introgression to complement the *f*_*4*_ test. This included pairwise estimates of D_xy_ between each San Salvador Island species and outgroups, TREEMIX analyses used to infer admixture events on population graphs [63], presence of alternative topologies in the regions, and maximum likelihood trees supporting close relationships between outgroups and either of the specialists. *f*_*4*_ outliers that appeared in extensive regions of low diversity in all three San Salvador Island species and did not have supporting evidence from other statistics or trees were excluded from the list of candidates as potential false positives in areas of low recombination (n = 2; S10 and S11 Figs).

### Multiple sources of genetic variation underlie species divergence

The relationships observed in the three alternative topologies (Fig 2) underlie most of the divergence observed between the molluscivores and scale-eaters: 75% and 88% of the fixed SNPs between molluscivores and large-jawed scale-eaters and molluscivores and all scale-eaters, respectively, fall within these topologies that make up less than 5% of the genome in total. Many of these regions contained candidate genes previously associated with variation in *Cyprinodon* jaw size within the San Salvador Island radiation [55]: 18 of the 31 candidate jaw genes occurred in the combined scale-eater topology and 1 candidate region in the molluscivore topology.

**Fig 4:**
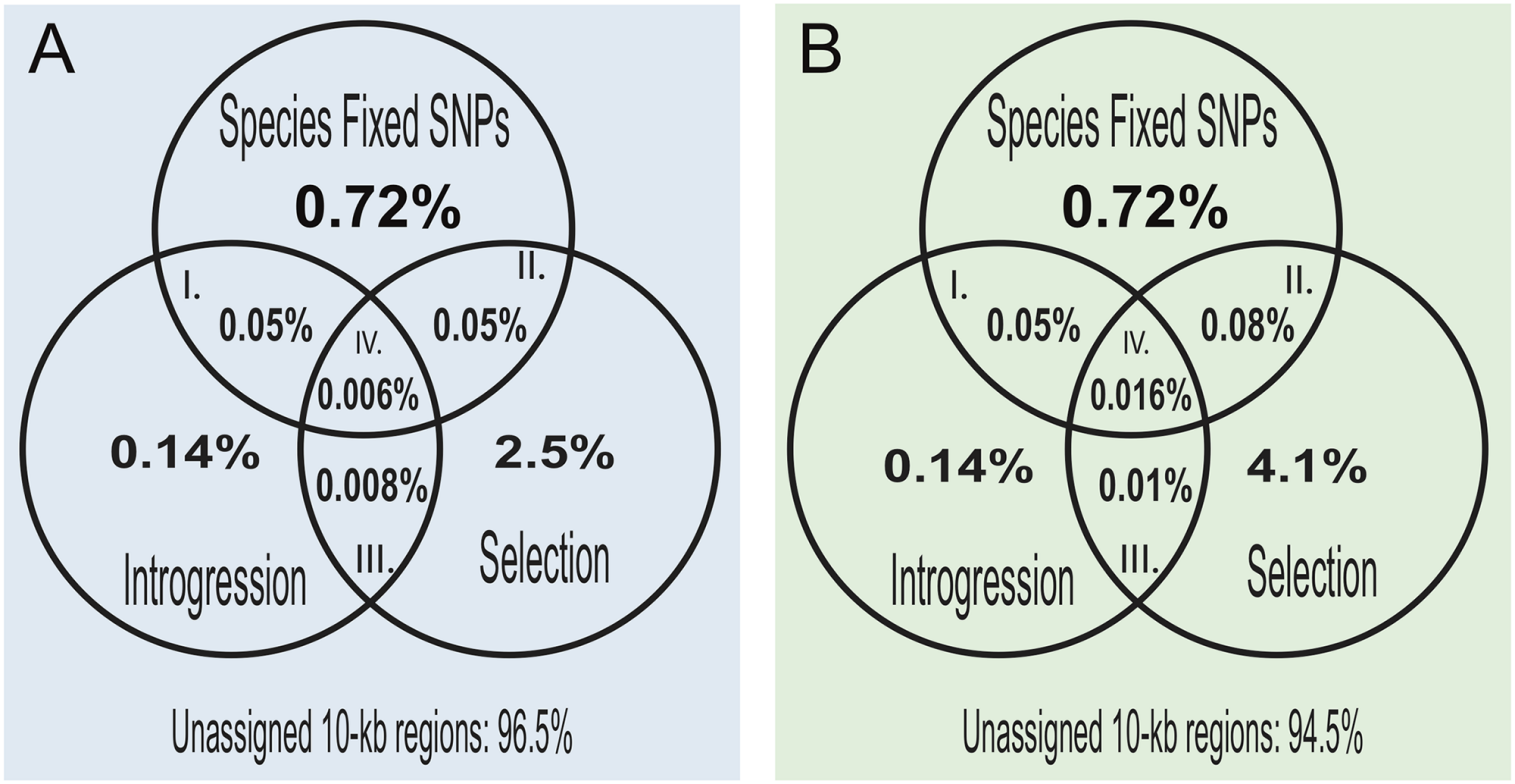
Contributions of selection and introgression to species divergence in the San Salvador Island specialists. Venn diagrams of the contribution of different sources of genetic variation to speciation in this system based on fixed SNPs between the molluscivore and combined scale-eaters, significant *f*_*4*_ values of introgression, hard selective sweeps (the lower 2% of the simulated distribution of Tajima’s D values) in (A) combined scale-eaters and (B) molluscivores. We calculated the percentage of I) Introgression: regions that contain introgressed genetic variation from the Caribbean contributing to species divergence but not under a selective sweep, II) Selective Sweeps: regions that have undergone strong selective sweeps from genetic variation on San Salvador Island that did not introgress from our two outgroup populations, III) Adaptive introgression: adaptively introgressed regions not contributing to species divergence, and IV) Adaptive introgression involved in speciation: regions that have undergone selective sweeps of introgressed variation that contributed to species divergence of the two specialist species. The percentage of 10-kb windows not assigned to the above categories is provided below each Venn diagram. Overlap of these categories with the dominant and alternative topologies is provided in S12 Fig.

We also assessed the relative contributions of different sources of genetic variation to the divergence between the two specialists (also see S12 Fig). Selective sweeps of introgressed variation from our two focal outgroups contributed 5 and 8 times less to species divergence between the scale-eaters and molluscivores, respectively, than sweeps of other sources of genetic variation (Fig 4). Adaptive introgression in regions of high divergence among the specialists appears to be rare, occurring in only 0.006 and 0.016% of the scale-eater and molluscivore genomes, respectively. The higher percentage in the molluscivore genome may be due to stronger bottlenecks in their past than in the scale-eaters, rather than more selective sweeps in this species. Within individual lakes, molluscivores have lower genetic diversity than both scale-eaters and generalists [57]. When segments are additionally separated based on topology assigned by SAGUARO, the alternative topologies contained a greater proportion of regions with introgressed genetic variation and selective sweeps than those regions assigned to the dominant topology. None of the fixed SNPs in adaptive introgression candidates occurred in a segment assigned to the dominant topology (S12 Fig).

### Adaptive introgression contributed to localized adaptive radiation

In general, selective sweeps of introgressed genetic variation that contributed to species divergence between the specialists were rare. However, four of the 11 candidate adaptive introgression regions contained genes with known craniofacial effects in model organisms or have been strongly associated with oral jaw size variation in the specialists [55], the primary axis of diversification in this system (Table 1 and S3 Table). Only one of these, the proto-oncogene *ski*, has both known craniofacial effects and was associated with jaw size variation in the specialists. *Ski* encodes for a corepressor protein involved in the SMAD-dependent transcription growth factor B pathway [65–67]. Mutations in *ski* cause marked reductions in skeletal muscle mass, depressed nasal bridges, and shortened, thick lower jaw bones in mice [68,69] and malformed craniofacial cartilage and shortened lower jaws in zebrafish [70]. These phenotypic changes are remarkably similar to the novel craniofacial morphologies in San Salvador Island molluscivore pupfishes, including increased nasal/maxillary protrusion, shortened lower jaw, and thicker dentary and articular bones [52].

**Table 1:** Adaptive introgression candidates in San Salvador Island specialists. These 11 candidate regions feature significant *f*_*4*_ values and signatures of selective sweeps in specialists, SNPs fixed between specialists, and low genetic divergence (*D*_xy_) between *C*. *laciniatus* and one of the specialists. The number of fixed SNPs that were in coding positions of a gene are provided in parentheses after the total number in the region. The specialist(s) with a selective sweep detected in the 98^th^ percentile of the SweeD composite likelihood ratio test and the lowest levels of genetic diversity (π) and Tajima’s D estimates within the 2% lower tail of the simulated Tajima’s D distribution are listed for each region.

Gene	*f*_*4*_	Sweep	TREEMIX Directionality	Fixed SNPs	Segregating in generalist?	Low π	Low Tajima's D	GO terms
**ski**^†^	0.261**	molluscivores	*C*. *laciniatus* → molluscivores	3(1)	Yes	molluscivore	molluscivore	SMAD binding, cartilage development
**rbms3**	-0.274*	scale-eaters		1(0)	Yes	scale-eater	scale-eater	RNA binding
pard3^†^	-0.223**	scale-eaters		57(0)	Yes	scale-eater/molluscivore	scale-eater	embryonic eye morphogenesis, neuroblast proliferation
NA^†^	0.255**	scale-eaters		14(-)	Yes	scale-eater/molluscivore	scale-eater/molluscivore	-
nbea	-0.28**	molluscivores		40(0)	Yes	scale-eater/molluscivore	molluscivore	synapse assembly, dendrite development
celf4	0.246**	scale-eaters		27(0)	Yes	scale-eater/molluscivore	scale-eater	mRNA binding, alternative mRNA splicing
NA	-0.279**	molluscivores/scale-eaters		1(-)	Yes	molluscivore	molluscivore	-
ltbp2	-0.255**	molluscivores	*C*. *laciniatus* → scale-eaters	2(0)	Yes	molluscivore	molluscivore	microfibril proliferation, calcium ion binding,
srbd1	0.269*	molluscivores/scale-eaters	*C*. *laciniatus* → molluscivores	19(0)	Yes	scale-eater/molluscivore	molluscivore	nucleic acid binding, RNA binding
srbd1	-0.267**	molluscivores/scale-eaters	*C*. *laciniatus* → scale-eaters	20(0)	Yes	scale-eater/molluscivore	molluscivore	nucleic acid binding, RNA binding
mcu	-0.228**	molluscivores		7(0)	Yes	scale-eater/molluscivore	molluscivore/scale-eater	mitochondrial calcium homeostasis

*P-value = 0.001;

**P-value<0.001;

^†^ gene associated with oral jaw size morphology in San Salvador Island pupfish [55];

(-) unannotated region

**Fig 5:**
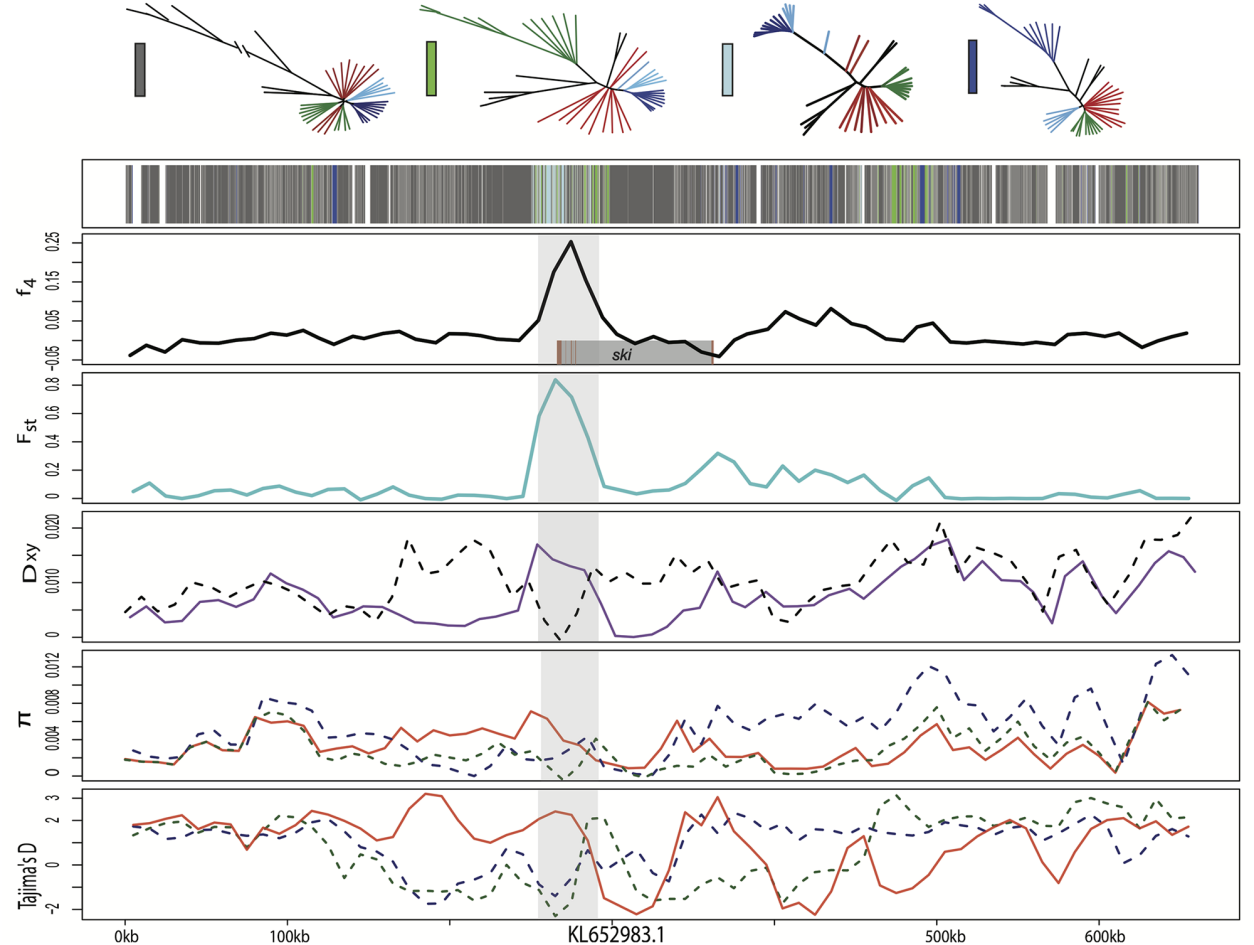
Candidate adaptive introgression region in craniofacial development gene *ski*. Fixed variants in this region were previously associated with pupfish oral jaw size [55]. Row 1 shows the topology assigned by SAGUARO to segments along a 600-kb scaffold (dark grey: dominant topology; blue: large-jawed scale-eater topology; light blue: combined scale-eater topology; green: molluscivore topology; light grey: all other topologies; white: unassigned segments). Row 2 shows average *f*_4_ value across non-overlapping 10-kb windows between mollsucivores/scale-eaters. Shaded grey box shows region annotated for *ski* gene with exons in red. Row 3 shows average *F*_*st*_ value across non-overlapping 10-kb windows between molluscivores/scale-eaters (turquoise). Row 4 shows between-population divergence (*D*_*xy*_) across non-overlapping 10-kb windows between molluscivores/scale-eaters (purple) and molluscivores/*C*. *laciniatus* (grey-dashed). Row 5 shows within-population diversity (*π*) across non-overlapping 10-kb windows (blue-dashed: scale-eater; green-dashed: molluscivores; red: generalist). Row 6 shows Tajima’s D across non-overlapping 10-kb windows (blue-dashed: scale-eater; green-dashed: molluscivores; red: generalists).

**Fig 6:**
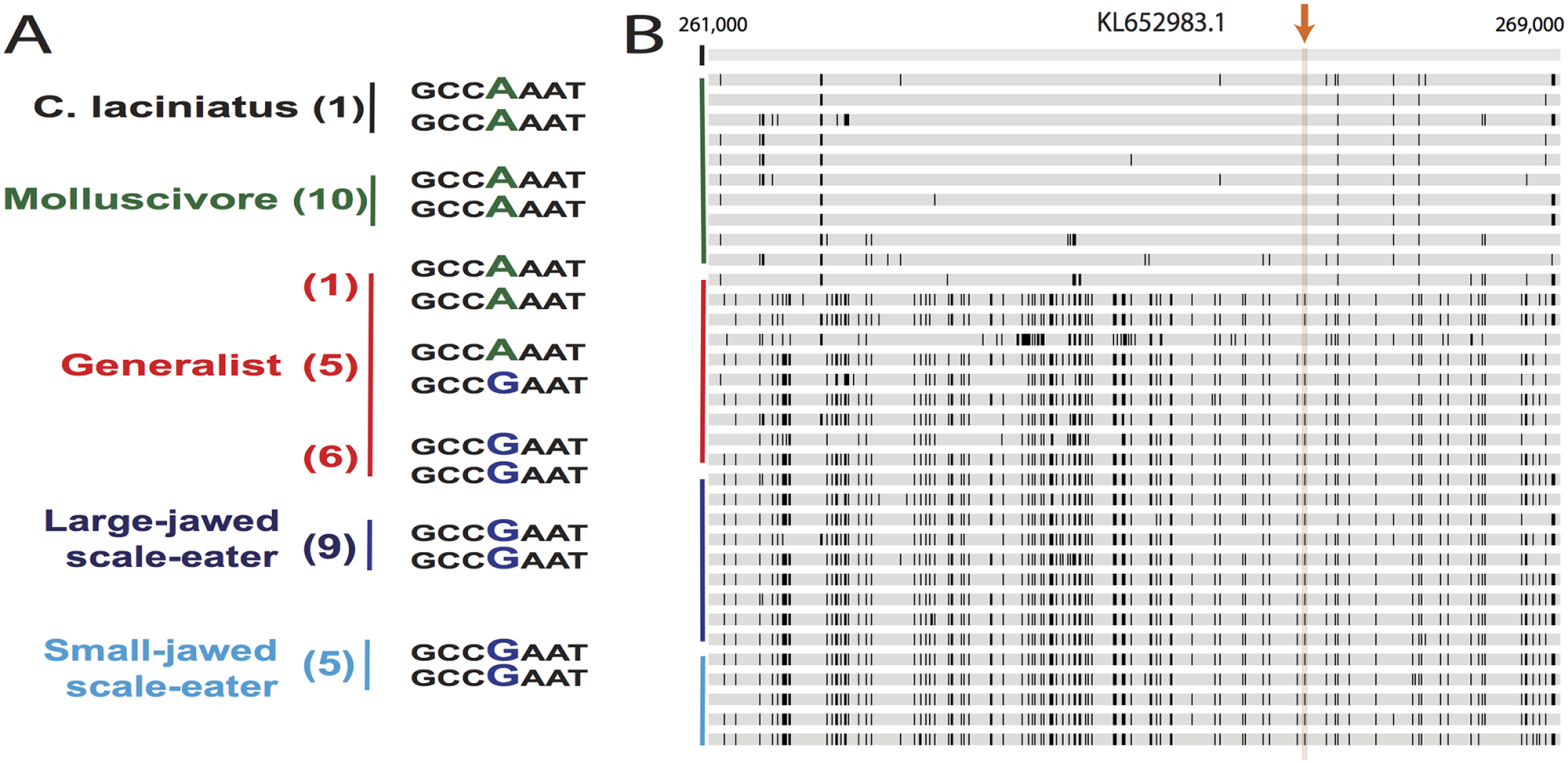
Genetic diversity surrounding the fixed variant in *ski* region assigned to the combined scale-eater topology. (A) The 3’ untranslated region variant fixed between the two specialists. The number of individuals with the haplotype(s) are located in parentheses next to species names. The other two fixed SNPs follow the same pattern across species as the SNP shown.(B) A comparison of the San Salvador Island genotypes (green = molluscivore; red = generalists; blue = scale-eater) with the *C*. *laciniatus* genotype (black) across an 8-kb window surrounding the fixed variant (orange arrow). The alleles that do not match the alleles of *C*. *laciniatus* are highlighted with black bars. The arrow points to the conflicting genotypes in the surrounding 8-kb region of the SNPs.

The candidate adaptive introgression region spans the start of *ski* and contains three fixed SNPs, one in the 3’ untranslated region, one in the 3^rd^ codon position of an exon, and one in an intron. This region contains a signature of high absolute genetic divergence between the two specialists and a selective sweep in the molluscivore (Fig 5). This region also features low nucleotide diversity within scale-eaters and negative estimates of Tajima’s D, although this does not appear to be as strong as in the molluscivores. Several lines of evidence point towards the introgression of *ski* variants between molluscivores and *C*. *laciniatus*. Genetic differentiation is minimal between molluscivores and *C*. *laciniatus* (*D*_*xy*_ = 0.0011) (Fig 5) and higher in all other pairwise comparisons (*D*_*xy*_ > 0.013) between the two specialists and two outgroup Caribbean pupfish species (S4 Table), indicating gene flow between the molluscivores on San Salvador Island and the generalist *C*. *laciniatus* on New Providence Island. Taking a closer look at the genetic variation in this region, we observe that the *ski* SNPs fixed in the San Salvador Island molluscivores are homozygous in *C*. *laciniatus* and segregating in the generalists (Fig 6A), suggesting that they occur at an appreciable frequency in the generalists. The surrounding molluscivore genetic background of the fixed *ski* SNPs is very similar to *C*. *laciniatus* (Fig 6B). In this 10-kb region, only 62 SNPs differ between the molluscivores and *C*. *laciniatus* in our sample. Segments of this region were assigned to the combined scale-eater topology (Fig 2C) and a maximum likelihood tree of the SAGUARO segment containing these three fixed SNPs features *C*. *laciniatus* in a clade with molluscivores (S13 Fig).

**Fig 7:**
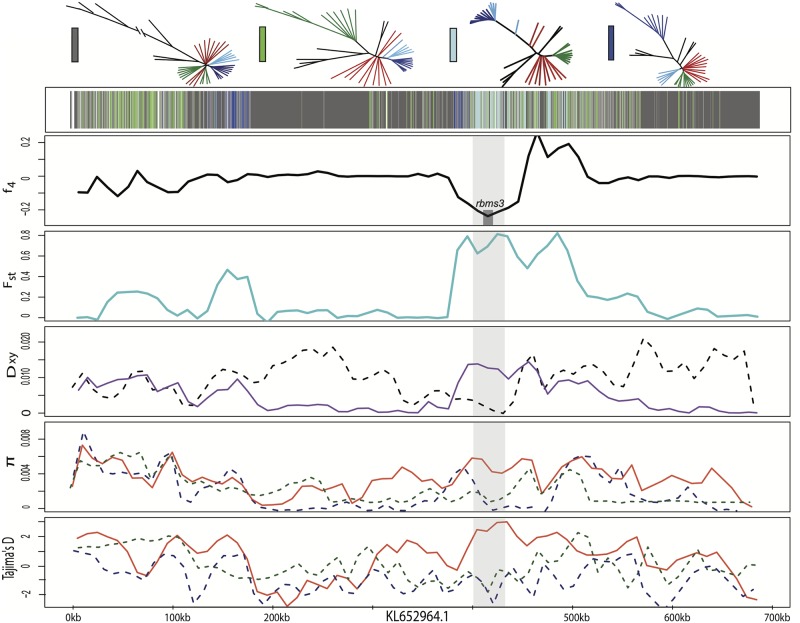
Candidate adaptive introgression region in craniofacial development gene *rbms3*. Row 1 shows the topology assigned by SAGUARO to segments along a 700-kb scaffold (dark grey: dominant topology; blue: large-jawed scale-eater topology; light blue: combined scale-eater topology; green: molluscivore topology; light grey: all other topologies; white: unassigned segments). Row 2 shows average *f*_4_ value across non-overlapping 10-kb windows between mollsucivores/scale-eaters. Shaded grey box shows region annotated for *rbms3* gene. Row 3 shows average *F*_*st*_ value across non-overlapping 10-kb windows between molluscivores/scale-eaters (turquoise). Row 4 shows between-population divergence (*D*_*xy*_) across non-overlapping 10-kb windows between molluscivores/scale-eaters (purple) and scale-eaters/*C*. *laciniatus* (grey-dashed). Row 5 shows within-population diversity (*π*) across non-overlapping 10-kb windows (blue-dashed: scale-eater; green-dashed: molluscivores; red: generalists). Row 6 shows Tajima’s D across non-overlapping 10-kb windows (blue-dashed: scale-eater; green-dashed: molluscivores; red: generalists).

In addition to *ski*, one other adaptively introgressed candidate region with known craniofacial effects in fish lies in the RNA-binding protein *rbms3*, a posttranscriptional regulator in the same SMAD-dependent transcription growth factor B pathway. Mutations in this gene cause cartilage and neural crest related abnormalities in zebrafish [71]. This region contains a non-coding SNP fixed in the San Salvador Island scale-eaters that is homozygous in *C*. *laciniatus* and segregating in the generalist population, a signature of high absolute genetic divergence between the two specialists, and a selective sweep in the scale-eater (Fig 7). Several lines of evidence point towards the introgression of *rbms3* variants between scale-eater and *C*. *laciniatus*. First, genetic differentiation is minimal between scale-eaters and *C*. *laciniatus* (*D*_*xy*_ = 0.002) and higher in all other pairwise comparisons (*D*_*xy*_ > 0.0104) between the two specialists and two outgroup Caribbean pupfish species (S4 Table). Segments of this region were assigned to the combined scale-eater topology (Fig 2C) and a maximum likelihood tree of the segment containing the fixed SNP features *C*. *laciniatus* in a clade with scale-eaters (S14 Fig). Similar to the pattern we find in *rbms3*, another candidate region previously associated with oral jaw size variation on San Salvador Island spanning *pard3* contained fixed scale-eater variants shared with *C*. *laciniatus*, strong genetic similarity in the surrounding region between the two and signs of a selective sweep in the scale-eaters (S15 and S16 Figs, Table 1).

In an unannotated candidate adaptive introgression region which has previously been associated with oral jaw size variation on San Salvador Island, we find a slightly different pattern than those mentioned above. The direction of introgression appears to be between *C*. *laciniatus* and the molluscivores, but is under a selective sweep in the scale-eaters (S17 and S18 Figs, Table 1 and S3 Table). We also see a similar pattern in *nbea*, where the direction of introgression appears to be between *C*. *laciniatus* and scale-eaters but is under a selective sweep in the molluscivores (S19 and S20 Figs, Table 1 and S3 Table). *Nbea* encodes for a scaffolding protein involved in neurotransmitter release and synaptic functioning and has been identified as a candidate gene for non-syndromic autism disorder [72–74]. In zebrafish, mutations disrupt electrical and chemical synapse formation and cause behavioral abnormalities such as decreased startle response [75]. Introgression in this regions is of interest because behavior is another axis of divergence between specialists in this system alongside craniofacial traits, as the species vary in mate choice [76,77], aggression, and prey capture behavior [54]. Both of these candidate regions feature nearly equivalent negative Tajima’s D statistics and low nucleotide diversity in the both of the specialists. The regions do not appear to be under strong selection in the generalist populations on San Salvador Island, so the signatures of selective sweeps in both specialists most likely stem from parallel molecular evolution in these regions rather than purifying selection in the ancestral population. Seven of 11 candidate regions show this pattern of equivalent low diversity and negative Tajima’s D statistics in both specialists (Table 1 and S3 Table).

The other 6 adaptive introgression candidates contained genes with a variety of functions including angiogenesis, calcium ion binding, embryonic eye morphogenesis, and RNA binding (Table 1) and had similar patterns to those mentioned above. Four of these regions feature low genetic diversity in both specialists. Two of these candidates lie in consecutive regions of the gene *srbd1*, which encodes for an RNA binding protein, and it appears that one has introgressed between the molluscivores and *C*. *laciniatus* and the other between scale-eaters and *C*. *laciniatus*. Both of these regions appear to be under a selective sweep in both of the specialists (Table 1 and S3 Table).

**Fig 8:**
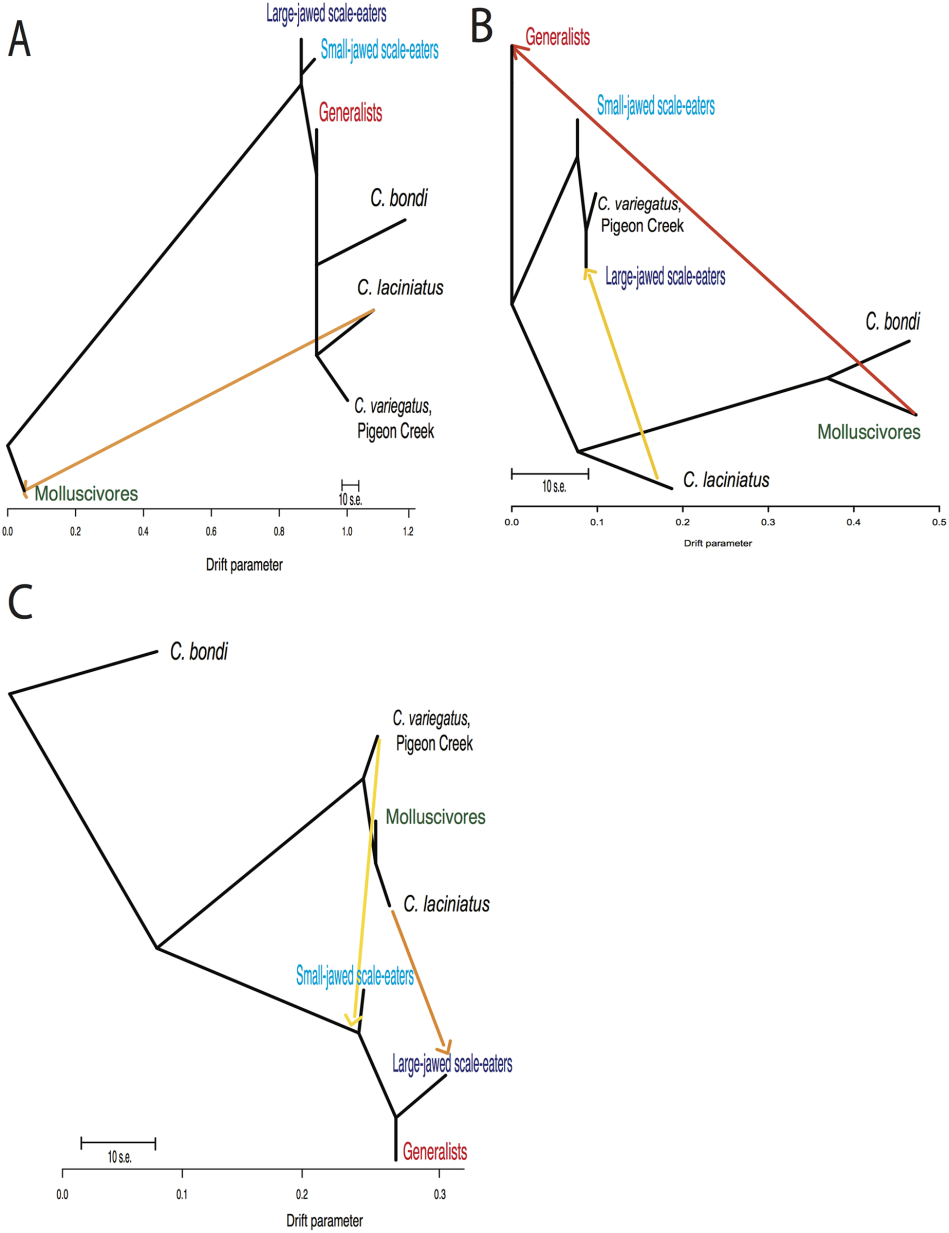
Introgression into the specialists on San Salvador Island from *C*. *laciniatus*. TREEMIX population graphs for three candidate adaptive introgression regions showing gene flow from *C*. *laciniatus* into the molluscivores in the (A) *ski* region (change in composite log-likelihood with increase in number of migration events: m = 0, LnL: -320; m = 1, LnL: 81; m = 2, LnL: 93), (B) *C. laciniatus* into the large-jawed scale-eaters in the *ltpb2* region (m = 0, LnL: 11; m = 1, LnL: 62; m = 2, LnL: 137; m = 3, LnL: 62), and (C) in the *srbd1* region (m = 0, LnL: -17; m = 1, LnL: -11; m = 2, LnL: 74; m = 3, LnL: 68).

Overall, potential adaptive variants contributing to species divergence among the specialists appear to be coming from New Providence Island in the northern Caribbean, rather than the southern Caribbean (Table 1 and S3 Table). Since it is impossible to infer the directionality of gene flow directly from *f*_*4*_ values, we used TREEMIX [63] to visualize gene flow in adaptively introgressed regions. Across the candidate adaptive introgression regions, we found evidence of an admixture event directly from *C*. *laciniatus* into the molluscivores in *ski* and *ltbp2* and *C*. *laciniatus* into scale-eaters in *srbd1* (Fig 8 and S5 Table). This suggests that genetic variation found on New Providence Island introgressed into the San Salvador Island radiation. There is no direct evidence from the TREEMIX population graphs of admixture from *C*. *bondi* into a specialist in the candidate regions (S5 Table), and D_xy_ between *C*. *bondi* and the specialists in pairwise comparisons is greater than those found between *C*. *laciniatus* and specialists across these regions (S3 Table). Both lines of evidence suggest that the high *f*_*4*_ values in these regions stem from gene flow between *C*. *laciniatus* and the specialists rather than *C*. *bondi*.

## Discussion

### Diverse sources of genetic variation contributed to a highly localized adaptive radiation

Our investigation of genetic variation reveals that multiple sources of genetic variation were important for the assembly of the complex phenotypes associated with the novel ecological transitions seen only on San Salvador Island, Bahamas. While species divergence appears to mostly come from selective sweeps of variation from San Salvador Island (Fig 4), rare adaptive introgression has also played a role in the radiation (Table 1; Figs 5 and 7, S15, S17 and S19 Figs). The adaptive introgression we found in this study has come from large admixture events into San Salvador Island from a generalist pupfish population on another Bahamian island approximately 300 km away. In contrast, we found no evidence of introgression from a generalist population 700 km away in the Dominican Republic in our top candidate regions (Table 1 and S5 Table), although it is impossible to rule out that candidate adaptive variants may also exist in this population at lower frequencies. Importantly, our limited sampling of one individual from each of two distant islands suggests that long-distance adaptive introgression is common and arises from abundant genetic variation found in only some parts of the Caribbean. An intriguing implication of these findings is that adaptive variants within the San Salvador Island radiation may have been partly assembled from the overlap of different pools of standing variation distributed across different parts of the Caribbean.

We found introgressed variants in four genes associated with the primary axis of jaw size variation within the radiation, as well as one in a gene with known behavioral effects in zebrafish. Both specialists appear to have candidate introgressed adaptive variants implicated in jaw morphology. Our best candidate for molluscivores was a region containing three fixed variants previously associated with jaw size variation on San Salvador Island in the proto-oncogene *ski*, which introgressed from *C*. *laciniatus*, another pupfish species on an island 300 km away (Figs 5, 6 and 8A, Table 1). The best candidate for scale-eaters was a region containing a single fixed variant in the gene *rbms3* (Fig 7, Table 1), which is also present in *C*. *laciniatus*. Other candidate regions contained genes with functions in behavior, angiogenesis, calcium ion binding, embryonic eye morphogenesis, and RNA binding (Table 1).

We rarely know the source of candidate variants involved in diversification or the contributions of multiple sources of genetic variation to rapid diversification. Genomic investigations of other adaptive radiations have also inferred roles for multiple genetic sources contributing to rapid diversification. For example, in the apple maggot fly, ancient gene flow from Mexican populations introduced an inversion affecting key diapause traits that aided the sympatric host shift to apples in the United States [78]. Hybridization within Darwin’s finches also appears to play a role in the origin of new lineages through adaptive introgression of functional loci contributing to beak shape differences between species [21]. In a *Mimulus* species complex, introgression of a locus affecting flower color appears to have been a driver of adaptation in the early stages of their diversification [79]. However, even in case studies demonstrating multiple sources of genetic variation, the relative contributions to the diverse ecological traits in these radiations still remain unknown in most cases (but see [80]).

### The genomic landscape of introgression differs between sympatric trophic specialists

Only 10% of all introgressed regions in either the molluscivore or scale-eater were shared between the two. This minimal overlap may reflect the complexity of different performance demands. Performance in the two specialists involves very different sets of functional traits (i.e. higher mechanical advantage and a novel nasal protrusion in the molluscivores vs. enlarged oral jaws and adductor muscles in the scale-eaters [54]) and divergent selective regimes (narrow and shallow vs. wide and deep fitness valleys [53,81,82]). The extensive variability in the genetic variation that introgressed between the two specialists may reflect multidimensional adaptation to two distinct trophic niches in this radiation, rather than variation along a linear axis (e.g. see [83–88]).

### Did introgression trigger adaptive radiation?

Although introgression is rare and localized across the genome, it was likely important for the assembly of the complex phenotypes observed on San Salvador Island (e.g. *ski and rbms3*). Our findings suggest two alternative possibilities. One intriguing possibility is that rare introgression of the necessary adaptive alleles into San Salvador Island may have been required to trigger the radiation in the presence of ecological opportunity. Indeed, a paradox in this system is why generalist populations in hypersaline lakes on neighboring islands with similar levels of ecological opportunity, lake areas, and overall genetic diversity have not radiated [53]. Alternatively, adaptive radiation on San Salvador Island may have initiated from standing and *de novo* variation and only later benefited from introgressed alleles to further refine species phenotypes. Of course, these scenarios are not mutually exclusive and may vary across loci. Based on our TREEMIX analysis, introgression from *C*. *laciniatus* into the molluscivores brought the *ski* variants (Fig 8A), but the candidate adaptive variants in this region are also segregating in the generalist population (Fig 6).

We can roughly estimate the timing of introgression for this *ski* region from the number of variants that have accumulated between the *C*. *laciniatus* and molluscivore haplotypes (*n* = 62 differences; Fig 6). Assuming neutrality, the observed genetic differences between the two lineages should equal 2μt, the time since their divergence in each lineage and μ, the mutation rate [89]. Using mutation rate estimates ranging from 5.37x10^-7^ (phylogeny-based estimate of *Cyprinodon* substitution rate [90]) to 1.32x10^-7^ mutations site^-1^ year^-1^ (estimated from a cichlid pedigree estimate of the per generation mutation rate [91] using a pupfish generation time of 6 months), introgression of the *ski* adaptive haplotype from *C*. *laciniatus* into the molluscivore specialist occurred between 5,700 to 23,500 years ago. The 10,000 year age estimate of the San Salvador Island radiation (based on estimates of dry lakes on the island [49–51]) falls within this window. This suggests the intriguing scenario in which widespread introgression during the last glacial maximum may have triggered adaptive radiation within pupfish populations isolated in the saline lakes of San Salvador Island during their initial formation. The 10-fold larger land mass of the Great Bahama Bank during this time could have created the opportunity for larger pupfish populations and greater genetic diversity. These pupfish populations would have been connected more extensively across the region than currently by the increased expanses of coastline habitats on the exposed bank. However, these are only exploratory inferences of the directionality of gene flow and timing of introgression. They should be confirmed with demographic analyses focused on testing different scenarios of admixture into San Salvador Island (e.g. [26,90,92–95]).

While there are rare and convincing examples of hybridization leading to homoploid speciation (reviewed in [47]), no study, including ours, has yet provided convincing evidence that hybridization was directly involved in triggering an adaptive radiation. For example, while there is strong evidence in Darwin’s finches that adaptive introgression of a loci controlling beak shape has contributed to phenotypic diversity of finches in the Galapagos, this hybridization occurred between members within the radiation [21]. Similarly, a recent study argued that hybridization between ancestral lineages of the Lake Victoria superflock cichlid radiations and distant riverine cichlid lineages fueled the radiations, based on evidence of equal admixture proportions across the genomes of the Victorian radiations from the riverine lineages and the presence of allelic variation in opsins in the riverine lineages which are also important in the Victoria radiation [38]. However, the timing of introgression and necessity of introgressed alleles for initiating adaptive radiations remains unclear in these systems, including our own. Admittedly, hybridization as the necessary and sufficient trigger of adaptive radiation is a difficult prediction to test.

Those examples with more direct evidence linking hybridization to adaptation and reproductive isolation within a radiation are often special cases where a single introgressed adaptive allele automatically results in increased reproductive isolation. Examples include introgressed adaptive loci controlling wing patterns in *Heliconius* butterflies involved in mimicry and mate selection [28,96], a locus controlling copper tolerance in *Mimulus* that is tightly associated with one causing hybrid lethality [16], and loci contributing to differing insecticide resistance in the M/S mosquito mating types [97–99]. While these cases provide convincing evidence that adaptive introgression can facilitate both ecological divergence and reproductive isolation, it is still unclear whether this introgression has actually triggered or simply contributed to the ongoing process of adaptive radiation.

Truly addressing the question of whether adaptive introgression triggered the radiation on San Salvador Island will require a better understanding of the timing of introgression and the necessity of introgressed variation for the speciation process. Although we have candidate alleles (e.g. in *ski* and *rbms3*) that we think play a role in the evolution of complex specialist phenotypes, it still remains unclear what minimal set of alleles is necessary for the major ecological transitions in this system. Knowledge of the age of variants important for these transitions, and whether these variants are present and adaptive in the other non-radiating lineages of Caribbean generalist populations is needed. Estimation of the age of introgressed variation relative to standing or *de novo* could also shed light on whether adaptive introgression simply contributed to an ongoing diversification process or triggered it on San Salvador Island.

### A new small-jawed scale-eating species within the radiation?

We also found evidence of a distinct clade of small-jawed scale-eaters, separate from the large-jawed scale-eaters (Figs 1 and 2). The consistent clustering of this clade across the genome suggests that they may be a distinct, partially reproductively isolated population on San Salvador Island, rather than a product of hybridization between generalists and scale-eaters in the lakes where they exist sympatrically (Figs 1 and 2; S1 and S2 Figs). They have only been observed in six lakes connected to the Great Lake System on San Salvador Island (Great Lake, Mermaid’s Pond, Osprey Pond, Oyster Pond, Little Lake, and Stout’s Lake), but not in isolated lakes such as Crescent Pond. Consistent with this pattern of occurrence, F_2_ hybrid phenotypes resembling the scale-eaters have previously been shown to have extremely low survival and growth rates in these isolated lakes [81].

Small-jawed scale-eaters may represent a viable intermediate ecotype on the evolutionary path toward more specialized scale-eating. Small-jawed scale-eater diets appear to be consistent with intermediate levels of scale-eating. Preliminary gut content analyses revealed that scales were found in the stomachs of 33% of small-jawed scale-eaters (*n* = 33) compared to 91% of large-jawed scale-eaters (*n* = 53). The idea that specialization can open the door to further specialization has been seen in other systems, including pollinator syndromes for bees, hummingbirds, and hawkmoths in *Mimulus* [100–102], Darwin’s ground finch specializing on blood on two islands in their range [103], and transitions in mammals between omnivory, carnivory, and herbivory [104]. If small-jawed scale-eaters represent an ecotype stepping stone on the path toward more specialized scale-eating, we might expect regions of the genome to reflect a nested relationship between the large-jawed and small-jawed scale-eaters. We see this predicted pattern in the combined scale-eater topology that underlies most of the fixed variants between the two scale-eating species (Fig 2).

If the small-jawed scale-eaters were instead the result of recent or recurrent hybridization events, we would expect certain patterns of large-jawed scale-eater and generalist ancestry across their genomes. For example, if they represent F_1_ hybrids, they should have equal ancestry from the two parental species across their genomes. The lack of fit in all five small-jawed scale-eater individuals to the ancestry proportions excepted if they represent F_1_ hybrids, F_2_ hybrids, or backcrosses to parental species (S2 Table) suggests that the small-jawed scale-eaters are not the result of such recent hybridization events, although they might have resulted from more complicated scenarios of hybridization that do not follow these simple patterns of ancestry [105,106]. LD does appear to be stronger in the small-jawed scaler-eaters than in the three San Salvador Island species (S3 Fig), a pattern expected in recent hybrids of distinct populations. These small-jawed scale-eaters may indeed be the products of ongoing or recent gene flow on San Salvador Island. A reconstruction of the history of gene flow among San Salvador Island species from demographic modeling with a larger sample, along with estimates of selection and reproductive isolation in the small-jawed scale-eaters, will be needed to assess whether they represent the products of ongoing gene flow on San Salvador Island or a potential new ecomorph.

### Conclusion

Here we demonstrate that the complex phenotypes associated with the novel ecological transitions within a nascent adaptive radiation of San Salvador Island pupfishes arose from multiple sources of genetic variation spread across the Caribbean. The variation important to this radiation is localized to small regions across the genome that are obscured by genome-wide summaries of the history of the radiation. Species divergence appears to mostly come from selective sweeps of standing or *de novo* genetic variation on San Salvador Island, but rare adaptive introgression events may also be necessary for the evolution of trophic specialists. This genomic landscape of introgression is variable between the specialists and has come from large admixture events from populations as far as 700 km across the Caribbean, although all top adaptive introgression candidates appear to have introgressed from a population 300 km away in the northwestern Bahamas. Our findings that multiple sources of genetic variation contribute to the San Salvador Island radiation suggests a complex suite of factors, including rare adaptive introgression, may be required to trigger adaptive radiation in the presence of ecological opportunity.

## Methods

### Study system and sampling

Individual pupfish were caught in hypersaline lakes on San Salvador Island in the Bahamas with either a hand or seine net in 2011, 2013, and 2015. Samples were collected from eight isolated lakes on this island (Crescent Pond, Great Lake, Little Lake, Mermaid Pond, Moon Rock Pond, Oyster Lake, Osprey Lake, and Stout’s Lake) and one estuary (Pigeon Creek). 13 *Cyprinodon variegatus* were sampled from all eight lakes on San Salvador Island; 10 *C*. *brontotheroides* were sampled from four lakes; and 14 *C*. *desquamator* were sampled from six lakes. The specialist species occur in sympatry with the generalists in only some of the lakes. Individual pupfish that were collected from other localities outside of San Salvador Island served as outgroups to the San Salvador Island radiation, including *C*. *laciniatus* from Lake Cunningham, New Providence Island in the Bahamas, *C*. *bondi* from Etang Saumatre lake in the Dominican Republic, *C*. *diabolis* from Devil’s Hole in California (collected as a dead specimen by National Park Staff in 2012), as well as captive-bred individuals of extinct-in-the-wild species *C*. *simus* and *C*. *maya* originating from Laguna Chichancanab, Quintana Roo, Mexico. Fish were euthanized by an overdose of buffered MS-222 (Finquel, Inc.) following approved protocols from University of California, Davis Institutional Animal Care and Use Committee (#17455) and University of California, Berkeley Animal Care and Use Committee (AUP-2015-01-7053) and stored in 95–100% ethanol. Only degraded tissue was available for *C*. *diabolis*, as described in [90]. Field research and export/collection permits were authorized by the BEST Commission in the Bahamas, the Ministry of Protected Areas and Biodiversity in the Dominican Republic, and the U.S. Fish & Wildlife Service and National Park Service.

### Genomic sequencing and bioinformatics

DNA was extracted from muscle tissue using DNeasy Blood and Tissue kits (Qiagen, Inc.) and quantified on a Qubit 3.0 fluorometer (Thermofisher Scientific, Inc.). Genomic libraries were prepared using the automated Apollo 324 system (WaferGen Biosystems, Inc.) at the Vincent J. Coates Genomic Sequencing Center (QB3). Samples were fragmented using Covaris sonication, barcoded with Illumina indices, and quality checked using a Fragment Analyzer (Advanced Analytical Technologies, Inc.). Nine to ten samples were pooled in four different libraries for 150PE sequencing on four lanes of an Illumina Hiseq4000.

2.8 billion raw reads were mapped from 42 individuals to the *Cyprinodon* reference genome (NCBI, *C*. *variegatus* Annotation Release 100, total sequence length = 1,035,184,475; number of scaffold = 9,259, scaffold N50, = 835,301; contig N50 = 20,803) with the Burrows-Wheeler Alignment Tool [107] (v 0.7.12). Duplicate reads were identified using MarkDuplicates and BAM indices were created using BuildBamIndex in the Picard software package (http://picard.sourceforge.net(v.2.0.1)). We followed the best practices guide recommended in the Genome Analysis Toolkit [108](v 3.5) to call and refine our SNP variant dataset using the program HaplotypeCaller. We filtered SNPs based on the recommended hard filter criteria (i.e. QD < 2.0; FS < 60; MQRankSum < -12.5; ReadPosRankSum < -8) [97,108] because we lacked high-quality known variants for these non-model species. Our final dataset after filtering contained 16 million variants and a mean sequencing coverage of 7.2X per individual (range: 5.2–9.3X).

### Characterization of genomic heterogeneity in evolutionary relationships among individuals

We used the machine learning program SAGUARO [56] to identify regions of the genome that contain different signals about the evolutionary relationships across San Salvador Island and outgroup *Cyprinodon* species. Saguaro combines a hidden Markov model with a self-organizing map (SOM) to characterize local phylogenetic relationships among individuals without requiring *a priori* hypotheses about the relationships. When diploid data is used, the SOM selects one allele at random for training. This method infers local relationships among individuals in the form of genetic distance matrices and assigns segments across the genomes to these topologies. These genetic distance matrices can then be transformed into neighborhood joining trees to visualize patterns of evolutionary relatedness across the genome. Three independent runs of SAGUARO were started using the program’s default settings and each was allowed to assign 15 different topologies across the genome. To determine how many topologies to estimate, analogous to a scree plot [109,110], we plotted the proportion of the genome explained by each hypothesized topology and looked for an inflection point (S21 Fig). We also looked at the neighborhood joining trees to assess whether additional topologies were informative about the evolutionary relationships among individuals (S21 Fig). The 15^th^ topology and additional topologies that we investigated tended to be uninformative about the evolutionary relationships among individuals and represented less than 0.5% of the genome. We excluded the last topology (15^th^) from downstream analyses due to lack of genetic distinction at both the level of populations and species included in the proposed genetic distance matrix and the low percentage of the genome assigned to it. The 14 topologies included in downstream analyses and the total percentages of the genome assigned to them were robust across all three independent runs. These topologies also appeared to be fairly robust to the influences of poorly mapped regions in the genome. We generated a mask file to identify poorly mapped regions in our dataset using the program SNPable (http://bit.ly/snpable; k-mer length = 50, and ‘stringency’ = 0.5) and removed these segments from downstream analyses of the topologies. Rerunning the SAGUARO analysis on the masked dataset resulted in very similar trees across the 14 different topologies, with the exception of several generalist individuals grouping with molluscivores in the molluscivore topology (S22 Fig).

### Comparison of linkage disequilibrium among San Salvador Island species

We calculated LD within each of the San Salvador Island species and compared it to estimates for the small-jawed scale-eaters to look for patterns of high linkage consistent with recent hybridization events. Pairwise LD across the largest scaffold in our dataset (4.2 Mb) was calculated for each species using the ‘r2 inter-chr’ function in PLINK v1.90 [111] for five individuals. These were chosen from a pool of individuals from Great Lake system populations (average genome-wide F_st_ < 0.05 across these lakes for each of the species) to balance the effects of small sample sizes and population structure on estimates of LD and more accurately compare LD decay between species. LD may be overestimated for each of the species due to the small number of individuals available to calculate it from in this study, and should be compared to estimates from other studies with caution.

### Characterization of introgression patterns across the genome

We characterized the heterogeneity in introgression across the genome using *f*_*4*_ statistics that were initially developed to test for introgression among human populations [61–63]. The *f*_*4*_ statistic tests if branches among a four-taxon tree lack residual genotypic covariance (as expected in the presence of incomplete lineage sorting and no introgression) by comparing allele frequencies among the three possible unrooted trees. A previous study [53] provided evidence of potential admixture with the Caribbean outgroup species used in this study, preventing their use in a D-statistic framework which requires designation of an outgroup with no potential introgression.

To look for evidence of gene flow across the Caribbean, we focused on tests of introgression with the two outgroup clades from our sample that came from other Caribbean islands in the Bahamas and Dominican Republic. Based on the tree ((P1, P2),(*C*. *laciniatus*, *C*. *bondi*)), *f*_*4*_ statistics were calculated for all three possible combinations of P1,P2 among the pooled populations of generalists, scale-eaters, and molluscivores on San Salvador Island. These *f*_*4*_ statistics were calculated using the population allele frequencies of biallelic SNPs and summarized over windows of 10 kb with a minimum of 50 variant sites using a custom python script (modified from ABBABABA.py created by Simon H. Martin, available on https://github.com/simonhmartin/genomics_general; [64]; our modified version is provided in the supplemental material), allowing for up to 10% missing data within a population per site. All 10 molluscivore and 14 scale-eater individuals from San Salvador Island were used in the tests for comparison to the molluscivore and combined scale-eater topologies, respectively. In another calculation of *f*_*4*_ statistics across the genome, the 5 small-jawed scale-eater individuals were excluded for the comparison to the large-jawed scale-eater topology. Although only single individuals from New Providence Island, Bahamas and the Dominican Republic were used to represent *C*. *laciniatus* and *C*. *bondi* in the *f*_*4*_ tests, the individuals that were sequenced are a random sample from these populations and should be representative. This resulted in 100,276 *f*_*4*_ statistics (mean *f*_*4*_ = -2x10^-4^) calculated across the genome for the test that included all scale-eaters and 100,097 *f*_*4*_ statistics (mean *f*_*4*_ = -9x10^-5^) for the test excluding the small-jawed scale-eaters.

We conducted 1,000 permutations of the *f*_*4*_ test to evaluate the significance of *f*_*4*_ values in sliding windows across the genome. For each permutation, individuals from the four original populations were randomly assigned without replacement to one of the four populations based on the tree ((P1,P2),(P3,P4)) to assess how likely a given *f*_*4*_ value would be observed by chance within our empirical dataset. We calculated the 1% tails of this null distribution and used these thresholds for our candidate introgressed regions (i.e. significant at alpha = 0.02). The null distribution illustrating the 1^st^ and 99^th^ quantiles for all combinations of the sliding window *f*_*4*_ test are provided in the supplementary material (S23 Fig). Each candidate introgressed region was assigned a *P*-value by counting the number of permutations that had an *f*_*4*_ value greater than (or lesser than if the *f*_*4*_ value was negative) or equal to the observed value.

It is difficult to distinguish between genetic variation that is similar among taxa due to introgression from a hybridization event and that from ancestral population structure, so some of the regions with significant *f*_*4*_ values may represent the biased assortment of genetic variation into modern lineages from a structured ancestral population [51]. A recent simulation study [64] found that extending the use of genome-wide introgression statistics such as Patterson’s D statistic to small genomic regions can result in a bias of detecting statistical outliers mostly in genomic regions of reduced diversity. Although it hasn’t been formally tested, *f*_*4*_ statistics may be subject to the same biases, so we additionally considered the nucleotide diversity present in outlier *f*_*4*_ regions in downstream analyses by comparing π across the detected regions of introgression in comparison to scaffold- and genome-wide estimates among the three San Salvador Island species.

### Comparison of patterns of introgression to patterns of genetic divergence and diversity

We then calculated several population genetic summary statistics in sliding windows across the genome to compare to the *f*_*4*_ patterns of introgression: F_st_, between-population nucleotide divergence (*D*_*xy*_), within-population nucleotide diversity (π) for pairwise species comparisons, and Tajima’s D estimates of selection in each species. D_xy_ between molluscivores and scale-eaters was calculated over the same 10-kb windows as the *f*_*4*_ tests using the python script popGenWindows.py created by Simon Martin (available on https://github.com/simonhmartin/genomics_general; [64]). Since our vcf file contained only variant sites and this script does not factor the missing sites into the calculation of *D*_*xy*_ by assuming they are invariant, we post-hoc incorporated the missing sites as invariant sites in the calculation of *D*_*xy*_. Missing sites in our dataset may include poorly aligned regions with lots of variants, so by assuming the missing sites are all invariant, *D*_*xy*_ may be underestimated in this study and should be compared to diversity values from other organisms with caution.

The remaining statistics were calculated in non-overlapping sliding windows of 10 kb using ‘wier-fst-pop’, ‘window-pi’, and ‘TajimaD’ functions in VCFtools v.0.1.14 [112]. Negative values of Tajima’s D indicate a reduction in nucleotide variation across segregating sites [113], which may result from hard selective sweeps due to positive selection. To determine regions of the genome potentially under positive selection, we created a null distribution of Tajima’s D values expected for each of three species under neutral coalescent theory using ms-move [114], a program that adds more flexibility in incorporating introgression events into the coalescent simulator ms [115]. Based on the demographic history estimated for the three San Salvador Island species in a previous study [55], we incorporated a 100-fold decrease in population size approximately 10,000 years ago (-eN 0.8 0.01) and an introgression event from one population into another to mimic introgression between a San Salvador Island species and an outgroup population at the beginning of the radiation (ex. -ej 0.8 2 1 –ev 0.8 2 1 0.1). We estimated the null distribution of Tajima’s D for 100,000 loci for 10–14 individuals with a variable number of segregating sites (ranging from 50 to the maximum observed in a 10-kb window of the genomes of each species). We modeled the timing of introgression from approximately 6,000–23,000 years (based on the rough estimate of the timing of introgression of *ski* in this study) with 10% of population composed of migrants (although the distribution appeared robust to variations in this fraction). Tajima’s D values were calculated from the simulated loci using the ‘sample stats’ feature available in the ms package [114]. The simulated introgression event and bottleneck skewed the null distribution towards negative Tajima’s D values (S24 Fig). Windows from the observed genomes that had Tajima’s D values in the lower 2% tail of the null distribution were considered candidate regions for selective sweeps.

We also estimated regions under selective sweeps from the expected neutral folded site frequency spectrum calculated with SweeD [116]. In this calculation, we included the bottleneck of a 100-fold decrease around 10,000 years ago and the recommended grid size of 1 kb across scaffolds to calculate the composite likelihood ratio (CLR) of a sweep. The values of CLR from 1 kb windows were averaged across 10-kb to compare with the other statistics calculated in windows. Windows with an average CLR estimate above the 98^th^ percentile across the background site frequency spectrum for their respective scaffold were considered candidate regions under a selective sweep.

We also used the function ‘wier-fst-pop’ to calculate *F*_*st*_ across individual SNPs to locate SNPs fixed between species and identify whether candidate adaptive introgression regions potentially contributed to species divergence. We assessed mean coverage across individuals at SNPs fixed between specialists and found that they ranged from 4.8–8.2x. The SNPs fixed in this study may be an overestimate of the variants potentially contributing to diversification in the specialists, as alleles may be missing from our individuals at these sites due to the low coverage. Average coverage and standard deviation across SNPs fixed in candidate regions are reported in the supplementary material (S3 Table). Only regions of overlap between significant *f*_*4*_ values, strongly negative Tajima’s D values, 98^th^ percentile CLR estimates, and fixed SNPs between the two specialists were considered candidate adaptive introgression regions that have contributed to species divergence. For each of these regions, we looked for annotated genes and searched their gene ontology in the phenotype database ‘Phenoscape’ [117–120] and AmiGO2 [121] for pertinent functions, particularly skeletal system effects. Skeletal features, particularly craniofacial morphologies such as jaw length, have extremely high rates of diversification among the species on San Salvador Island [48,53] and likely play a key role in the diversification of this group.

### Estimation of the direction of gene flow in candidate adaptive introgression regions

While the sign of *f*_*4*_ hints at the directionality of introgression (e.g. for the tree (P1,P2),(P3,P4), a positive *f*_*4*_ value indicates gene flow either between P1 and P3 or P2 and P4), the lack of an explicit outgroup in the *f*_*4*_ statistics makes it difficult to determine the exact direction of gene flow among the included populations and limits our ability to determine if candidate introgressed regions came from admixture with *C*. *laciniatus* or *C*. *bondi*. We examined each candidate region for signs of directionality using several methods.

To visualize gene flow among the Caribbean populations included in this study, we used TREEMIX v1.12 [63] to estimate population graphs with 0–4 admixture events connecting populations. Population graphs were estimated for each region with a significant *f*_*4*_ value, each with a minimum of 50 SNPs. The number of admixture events was estimated by comparing the rate of change in log likelihood of each additional event, an approach similar to one used in Evanno et al. ([122]; also see [53]). However, this analysis should be viewed only as an exploratory tool as the reliability of TREEMIX to detect the number of admixture events has not been tested. This method was designed to be applied on genome-wide allele frequencies and estimates covariance in allele frequencies among populations in branch lengths using a model that assumes allele frequency differences between populations are solely caused by genetic drift [63]. The use of fewer SNPs (≥50) in our window-based approach also makes it harder to reliably distinguish between the different likelihoods for the number of migration events. The reliability of inference under these conditions has not been evaluated, however the migration events inferred in our TREEMIX results were consistent with our findings from our formal *f*_*4*_ test for gene flow.

We also compared pairwise nucleotide diversity between *C*. *bondi*, *C*. *laciniatus*, molluscivores, and scale-eaters to determine which pairs are most genetically similar in the candidate introgression regions. Since our genomic dataset only included single individuals from *C*. *bondi* and *C*.*laciniatus* and *F*_*st*_ estimates are a relative measure of divergence based on within population diversity, we calculated *D*_*xy*_, an absolute measure of genetic divergence between-populations. Finally, we generated maximum likelihood phylogenetic trees for the SAGUAROsegment containing the fixed SNPs under a GTR+GAMMA model of sequence evolution using RaxML v.8.2.10 [123]. Support for nodes was assessed by bootstrapping, allowing the number of bootstraps determined by autoMRE function in RaxML, which ranged from 900–1,000 among regions.

## Supporting information

S1 Fig Topologies featuring a monophyletic San Salvador Island clade. Black lineages are the *Cyprinodon* outgroups, red lineages are the San Salvador Island generalists, green lineages are the San Salvador Island molluscivores, dark blue lineages are the large-jawed scale-eaters and light blue lineages are the small-jawed scale-eaters. Percentages indicate the proportion of the *Cyprinodon* genome assigned to each topology.
(TIFF)

S2 Fig Topologies featuring a non-monophyletic San Salvador Island clade. Black lineages are the *Cyprinodon* outgroups, red lineages are the San Salvador Island generalists, green lineages are the San Salvador Island molluscivores, dark blue lineages are the large-jawed scale-eaters and light blue lineages are the small jawed scale-eater. Percentages indicate the proportion of the *Cyprinodon* genome assigned to each topology.
(TIFF)

S3 Fig Linkage disequilibrium decay in San Salvador Island pupfishes. Average r^2^ values for pairwise SNPs A) within a distance of 500,000 bp of each other and B) across the entirety of the largest scaffold of the genome (KL652500.1, 4.2 Mb). r^2^ was calculated from 5 individuals of each of the San Salvador Island species: generalists (red), molluscivores (green), large-jawed scale-eaters (dark blue) and small-jawed scale-eaters (light blue). The black horizontal dashed line in panel A is arbitrarily set at r^2^ = 0.3 as a marker for comparing decay between the four groups.
(TIFF)

S4 Fig Visualization of introgression across the genomes of molluscivores and scale-eaters. Manhattan plot of the *f*_*4*_ values between the San Salvador Island molluscivores, scale-eaters, *C*. *laciniatus* from New Providence Island, Bahamas and *C*. *bondi* from Etang Saumautre, Dominican Republic. Alternating gray/black colors indicate different scaffolds, starting with the largest scaffolds in the top row and the smallest scaffolds in the bottom row. Dotted red lines mark the permutation based two-tailed significance level threshold of 0.02.
(TIFF)

S5 Fig Visualization of introgression across the genomes of molluscivores and generalists. Manhattan plot of the *f*_*4*_ values between the San Salvador Island molluscivores, generalists, *C*. *laciniatus* from New Providence Island, Bahamas and *C*. *bondi* from Etang Saumatre, Dominican Republic. Alternating gray/black colors indicate different scaffolds, starting with the largest scaffolds in the top row and the smallest scaffolds in the bottom row. Dotted red lines mark the permutation based two-tailed significance level threshold of 0.02.
(TIFF)

S6 Fig Visualization of introgression across the genomes of scale-eaters and generalists. Manhattan plot of the *f*_*4*_ values between the San Salvador Island large-jawed scale-eaters, small-jawed scale-eaters, *C*. *laciniatus* from New Providence Island, Bahamas and *C*. *bondi* from Etang Saumautre, Dominican Republic. Alternating gray/black colors indicate different scaffolds, starting with the largest scaffolds in the top row and the smallest scaffolds in the bottom row. Dotted red lines mark the permutation based two-tailed significance level thresholds of 0.02.
(TIFF)

S7 Fig Visualization of introgression across the genomes of large-jawed scale-eaters and molluscivores. Manhattan plot of the *f*_*4*_ values between the San Salvador Island large-jawed scale-eaters, molluscivores, *C*. *laciniatus* from New Providence Island, Bahamas and *C*. *bondi* from Etang Saumatre, Dominican Republic. Alternating gray/black colors indicate different scaffolds, starting with the largest scaffolds in the top row and the smallest scaffolds in the bottom row. Dotted red lines mark the permutation based two-tailed significance level thresholds of 0.02.
(TIFF)

S8 Fig Comparison of variance in *f*_*4*_ to genetic diversity statistics over 10-kb non-overlapping windows. The variance in *f*_*4*_ statistic of a region compared to within-population diversity in A) molluscivores B) scale-eaters, and C) generalists, D) and average within-population diversity in all three species.
(TIFF)

S9 Fig Comparison of *f*_*4*_ to genetic diversity statistics over 10-kb non-overlapping windows. Red dots indicate 10-kb regions with signals of introgression above permutations based significance level. The *f*_*4*_ statistic of a region compared to within-population diversity in A) molluscivores and scale-eaters B) scale-eaters and generalists, and C) molluscivores and generalists D) and average within-population diversity in all three species.
(TIFF)

S10 Fig Candidate adaptive introgression regions in gene *wnt7b* with low diversity in all San Salvador Island species. Row 1 shows the history assigned by SAGUARO to segments along a 700-kb scaffold (dark grey: dominant topology; blue: large-jawed scale-eater topology; light blue: combined scale-eater topology; green: molluscivore topology; light grey: all other topologies; white: unassigned segments). Row 2 shows average *f*_4_ value across non-overlapping 10-kb windows between molluscivores/scale-eaters. Shaded grey box shows region annotated for *ski* gene with exons in red. Row 3 shows average *F*_*st*_ value across non-overlapping 10-kb windows between molluscivores/scale-eaters (turquoise). Row 4 shows between-population divergence (*D*_*xy*_) across non-overlapping 10-kb windows between molluscivores/scale-eaters and molluscivores/*C*. *laciniatus* (grey-dashed). Row 5 shows within-population diversity (*π*) across non-overlapping 10-kb windows (blue-dashed: scale-eater; green: molluscivore). Row 6 shows Tajima’s D across non-overlapping 10-kb windows (blue-dashed: scale-eater; green: molluscivore.
(TIFF)

S11 Fig Candidate adaptive introgression region in gene *plekhg* with low diversity in all San Salvador species. Fixed variants in this region were previously associated with pupfish oral jaw size [55]. Row 1 shows the history assigned by SAGUARO to segments along a 200-kb scaffold (dark grey: dominant topology; blue: large-jawed scale-eater topology; light blue: combined scale-eater topology; green: molluscivore topology; light grey: all other topologies; white: unassigned segments). Row 2 shows average *f*_4_ value across non-overlapping 10-kb windows between mollsucivores/scale-eaters. Shaded grey box shows region annotated for *ski* gene with exons in red. Row 3 shows average *F*_*st*_ value across non-overlapping 10-kb windows between molluscivores/scale-eaters (turquoise). Row 4 shows between-population divergence (*D*_*xy*_) across non-overlapping 10-kb windows between molluscivores/scale-eaters and molluscivores/*C*. *laciniatus* (grey-dashed). Row 5 shows within-population diversity (*π*) across non-overlapping 10-kb windows (blue-dashed: scale-eater; green: molluscivore). Row 6 shows Tajima’s D across non-overlapping 10-kb windows (blue-dashed: scale-eater; green: molluscivore.
(TIFF)

S12 Fig The percentage of segments assigned to the monophyletic San Salvador Island and alternative topologies that contain signatures of species divergence, selection, and introgression. Venn diagrams of the contribution of different sources of genetic variation to speciation in this system based on the overlap of regions with fixed SNPs between the molluscivore and large-jawed scale-eater, significant *f*_*4*_ values of introgression, and Tajima’s D values below the simulation based lower one-tailed significance level of 0.02. Under each topology, we calculated the percentage of I) regions that contain introgressed genetic variation from the Caribbean contributing to species divergence, II) regions that have undergone strong selective sweeps from non-introgressed genetic variation on San Salvador Island, III) adaptively introgressed regions not contributing to species divergence, and IV) regions that have undergone selective sweeps of introgressed variation that contributed to species divergence of the two specialists (i.e. contain fixed SNPs between the specialists). The percentage of segments assigned to topologies, but not assigned to any of the above categories, are provided below the Venn diagrams.
(TIFF)

S13 Fig Maximum likelihood tree of the 4,753 bp SAGUARO segment containing the SNPs fixed between specialists in the gene *ski*. The names indicate the pond locality of the individuals (green: molluscivores; dark blue: large-jawed scale-eaters; light blue: small-jawed scale-eaters; black: pupfish outgroups). The scale bar indicates number of substitutions/bp.
(TIFF)

S14 Fig Maximum likelihood tree of the 2,788 bp SAGUARO segment containing the SNPs fixed between specialists in the gene *rbms3*. The names indicate the pond locality of the individuals (green: molluscivores; dark blue: large-jawed scale-eaters; light blue: small-jawed scale-eaters; black: pupfish outgroups). The scale bar indicates number of substitutions/bp.
(TIFF)

S15 Fig Candidate adaptive introgression region in the gene *pard3*. Fixed variants in this region were previously associated with pupfish oral jaw size [55]. Row 1 shows the history assigned by SAGUARO to segments along a 1-Mb scaffold (dark grey: dominant topology; blue: large-jawed scale-eater topology; light blue: combined scale-eater topology; green: molluscivore topology; light grey: all other topologies; white: unassigned segments). Row 2 shows average *f*_4_ value across non-overlapping 10-kb windows between mollsucivores/scale-eaters. Shaded grey box shows region annotated for *pard3* gene with exons in red. Row 3 shows average *F*_*st*_ value across non-overlapping 10-kb windows between molluscivores/scale-eaters (turquoise). Row 4 shows between-population divergence (*D*_*xy*_) across non-overlapping 10-kb windows between molluscivores/scale-eaters (purple) and scale-eaters/*C*. *laciniatus* (grey-dashed). Row 5 shows within-population diversity (*π*) across non-overlapping 10-kb windows (blue-dashed: scale-eater; green: molluscivore). Row 6 shows Tajima’s D across non-overlapping 10-kb windows (blue-dashed: scale-eater; green: molluscivore.
(TIFF)

S16 Fig Maximum likelihood tree of the 708 bp SAGUARO segment containing the SNPs fixed between specialists in the gene *pard3*. The names indicate the pond locality of the individuals (green: molluscivores; dark blue: large-jawed scale-eaters; light blue: small-jawed scale-eaters; black: pupfish outgroups). The scale bar indicates number of substitutions/bp.
(TIFF)

S17 Fig Candidate adaptive introgression region in an unannotated region. Fixed variants in this region were previously associated with pupfish oral jaw size [55]. Row 1 shows the history assigned by SAGUARO to segments along a 1.4-Mb scaffold (dark grey: dominant topology; blue: large-jawed scale-eater topology; light blue: combined scale-eater topology; green: molluscivore topology; light grey: all other topologies; white: unassigned segments). Row 2 shows average *f*_4_ value across non-overlapping 10-kb windows between mollsucivores/scale-eaters. Row 3 shows average *F*_*st*_ value across non-overlapping 10-kb windows between molluscivores/scale-eaters (turquoise). Row 4 shows between-population divergence (*D*_*xy*_) across non-overlapping 10-kb windows between molluscivores/scale-eaters (purple) and molluscivores/*C*. *laciniatus* (grey-dashed). Row 5 shows within-population diversity (*π*) across non-overlapping 10-kb windows (blue-dashed: scale-eater; green: molluscivore). Row 6 shows Tajima’s D across non-overlapping 10-kb windows (blue-dashed: scale-eater; green: molluscivore.
(TIFF)

S18 Fig Maximum likelihood tree of the 2,943 bp SAGUARO segment containing the SNPs fixed between specialists in the unannotated region on scaffold KL652649.1. The names indicate the pond locality of the individuals (green: molluscivores; dark blue: large-jawed scale-eaters; light blue: small-jawed scale-eaters; black: pupfish outgroups). The scale bar indicates number of substitutions/bp.
(TIFF)

S19 Fig Candidate adaptive introgression region within gene *nbea*. Row 1 shows the history assigned by SAGUARO to segments along an 800-kb scaffold (dark grey: dominant topology; blue: large-jawed scale-eater topology; light blue: combined scale-eater topology; green: molluscivore topology; light grey: all other topologies; white: unassigned segments). Row 2 shows average *f*_4_ value across non-overlapping 10-kb windows between mollsucivores/scale-eaters. Shaded grey box shows region annotated for *nbea* gene with exons in red. Row 3 shows average *F*_*st*_ value across non-overlapping 10-kb windows between molluscivores/scale-eaters (turquoise). Row 4 shows between-population divergence (*D*_*xy*_) across non-overlapping 10-kb windows between molluscivores/scale-eaters (purple) and scale-eaters/*C*. *laciniatus* (grey-dashed). Row 5 shows within-population diversity (*π*) across non-overlapping 10-kb windows (blue-dashed: scale-eater; green: molluscivore). Row 6 shows Tajima’s D across non-overlapping 10-kb windows (blue-dashed: scale-eater; green: molluscivore.
(TIFF)

S20 Fig Maximum likelihood tree of the 1,902 bp SAGUARO segment containing the SNPs fixed between specialists in the gene *nbea*. The names indicate the pond locality of the individuals (green: molluscivores; dark blue: large-jawed scale-eaters; light blue: small-jawed scale-eaters; black: pupfish outgroups). The scale bar indicates number of substitutions/bp.
(TIFF)

S21 Fig The proportion of the genome assigned to each topology by SAGRUARO. The insert is a closer look at the 13 topologies assigned to the smallest proportion of the genome and the largely uninformative 15^th^ topology. This suggests saturation in the variance explained by topologies at 14.
(TIFF)

S22 Fig Molluscivore tree at the end of 15 iterations of SAGUARO on the masked genomic dataset. Black lineages are the *Cyprinodon* outgroups, red lineages are the San Salvador Island generalists, green lineages are the San Salvador Island molluscivores, dark blue lineages are the large jawed scale-eaters and light blue lineages are the small jawed scale-eater. This topology differs from the molluscivore topology created from unmasked genomic dataset (Fig 2A) in that along with the molluscivores, generalists from Mermaid’s Pond, Osprey Lake, Little Lake, Crescent Pond, and Moon Rock Pond appear more closely related to outgroup populations than other San Salvador Island populations.
(TIFF)

S23 Fig The 1^st^ and 99^th^ quantiles of null distributions generated from permutations of the *f*_4_ statistic calculated across sliding windows of the genome. The red lines represent the 1^st^ quantile (left panels) and 99^th^ (right panels) observed *f*_*4*_ values with less than 1% chance of being in the null permutation based distributions of the *f*_*4*_ test combinations including a) molluscivores and scale-eaters, b) molluscivores and generalists, and c) scale-eaters and generalists.
(TIFF)

S24 Fig Distribution of Tajima’s D values from a coalescence simulation including a bottleneck and introgression. The red line represents the 2^nd^ percentile of the distribution and observed values greater than or equal to this were used to determine regions potentially under selective sweeps.
(TIFF)

S1 Table Hypothesized topologies from the SAGUARO analysis. (DOCX)

S2 Table Ancestry proportions expected of small-jawed scale-eaters if they represent hybrids of the large-jawed scale-eaters and generalists. Ancestry in small-jawed scale-eaters is assigned for SNPs fixed (n = 1,887) between the large-jawed scale-eaters and generalists. The genotype count in the 5 small-jawed scale-eater individuals at SNPs fixed between the large-jawed scale-eaters and generalists that are homozygous for one of the parental genotypes or heterozygous between the two (the proportion of loci in is provided in the parentheses; n = 1,887). The observed proportion of ancestry in small-jawed scale-eaters does not fit the proportions expected for F_1_ hybrids (all heterozygotes), backcross with the one of the parental species (half heterozgyous, half homozygous for parental allele), or F_2_ hybrids (half heterozygous, one-fourth homozygous for large-jawed scale-eater, and one-fourth homozygous for generalist). *X*^2^ value and P-value are provided for the *X*^2^ goodness-of-fit test of the observed proportions of ancestry across the genome to those expected of F_2_ hybrids.
(DOCX)

S3 Table 11 candidate adaptive introgression regions in San Salvador Island specialists. Adaptively introgressed regions and gene annotations for fixed SNPs between scale-eater and molluscivore species that lie in genomic regions assigned to one of the three alternative topologies. Asterisks (*) indicate SNPs in gene regions associated with San Salvador Island pupfish oral jaw size variation in a previous study [55]. Bolded genes have known functional effects on craniofacial traits in a model system. Regions that are not annotated for genes are indicated with a dash (-). *P*-values indicate the number of permutations of the candidate region with *f*_*4*_ values greater than or equal to the observed *f*_*4*_ value. The number of fixed SNPs that were in coding positions of a gene are provide in parentheses after the total number of fixed SNPs in the candidate adaptive introgression region. The specialist(s) with a selective sweep detected in the 98^th^ percentile of SweeD composite likelihood ratio test.
(DOCX)

S4 Table Pairwise genetic divergence (Dxy) between molluscivores, scale-eaters, Lake Cunningham, New Providence Island (*C*. *laciniatus*) and Etang Saumatre, Dominican Republic (*C*. *bondi*). NA*(2649) is the unannotated candidate adaptive introgression region on scaffold KL652649.1 and NA (3033) is the unannotated candidate adaptive introgression region on scaffold KL653033.1. The two species with the lowest Dxy are bolded for each region.
(DOCX)

S5 Table Summary of admixture events inferred by TREEMIX for the adaptive introgression candidate regions assigned to the three alternative topologies. San Salvador Island generalist (A), San Salvador Island large-jawed scale-eater (L), San Salvador Island small-jawed scale-eater (S), San Salvador Island molluscivore (M), *C*. *laciniatus* from New Providence Island Bahamas (CUN), *C*. *bondi* from Dominican Republic (ETA), most recent common ancestor of Caribbean pupfish lineages (MRC).
(DOCX)
