# Supplementary figures and images for: Adaptive introgression from distant Caribbean islands contributed to the diversification of a microendemic adaptive radiation of trophic specialist pupfishes
Source: PLoS Genet. 2017 Aug 10;13(8):e1006919. doi: 10.1371/journal.pgen.1006919 (PMC5552031; doi:10.1371/journal.pgen.1006919)

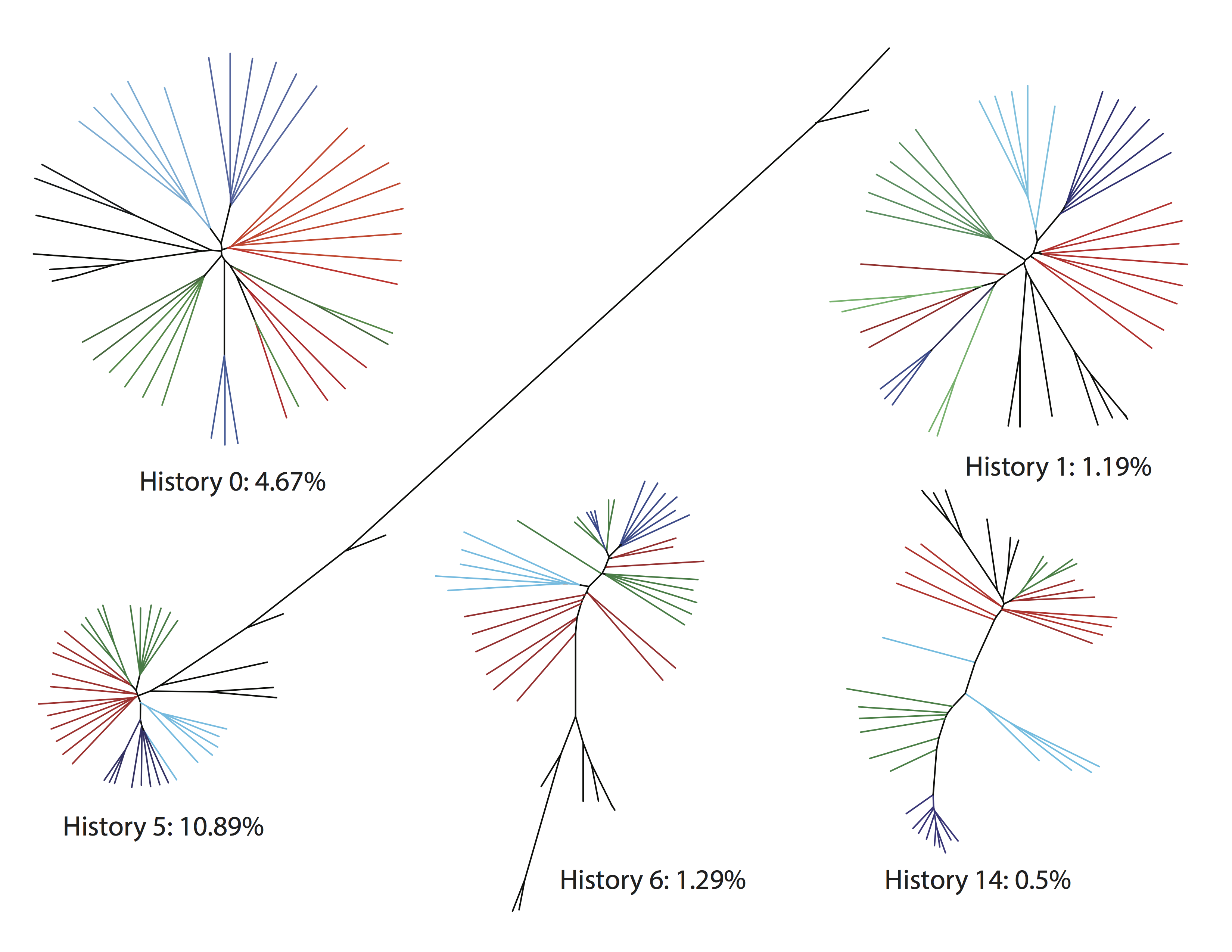

Supplement: S1 Fig — Black lineages are the Cyprinodon outgroups, red lineages are the San Salvador Island generalists, green lineages are the San Salvador Island molluscivores, dark blue lineages are the large-jawed scale-eaters and light blue lineages are the small-jawed scale-eaters. Percentages indicate the proportion of the Cyprinodon genome assigned to each topology. (TIFF) [file pgen.1006919.s001.tiff]

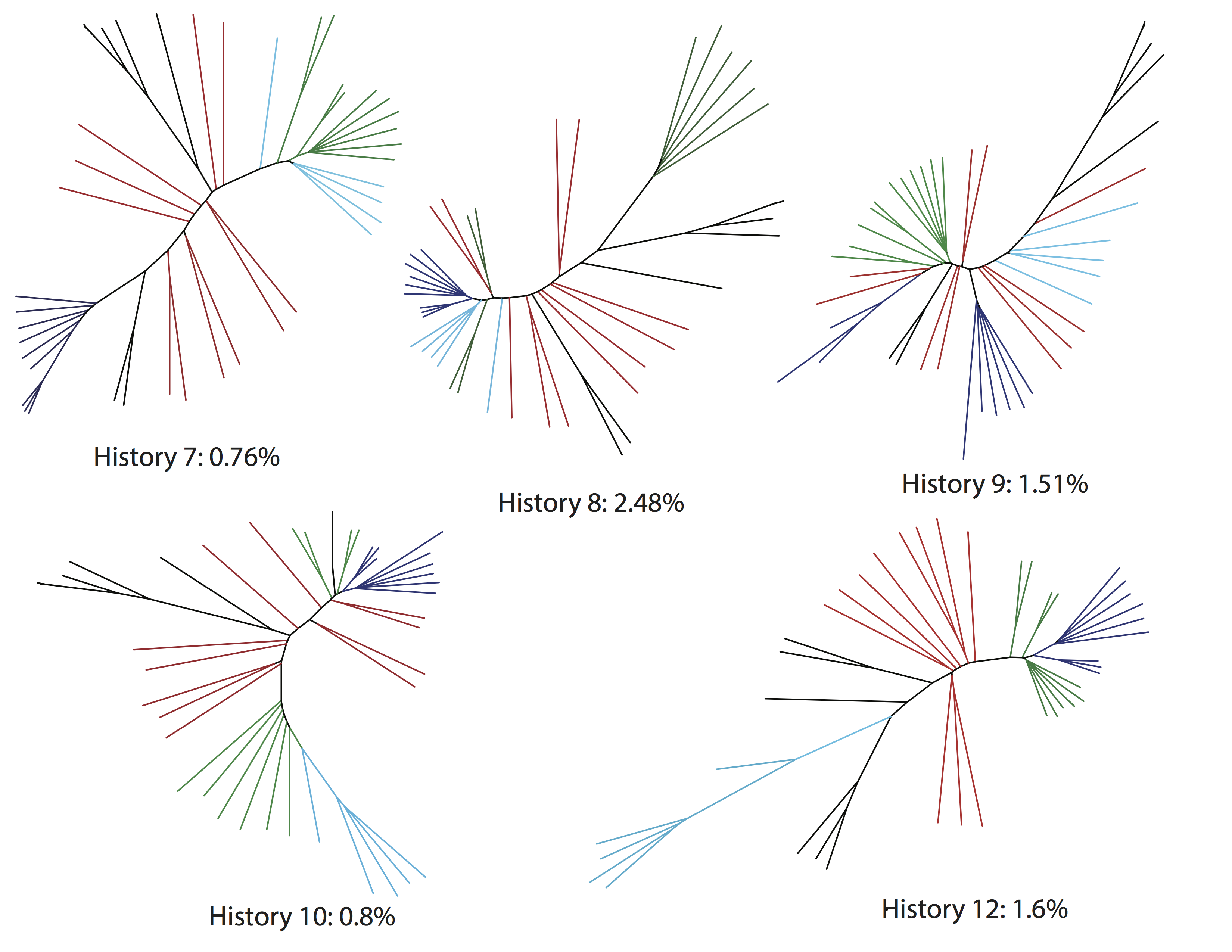

Supplement: S2 Fig — Black lineages are the Cyprinodon outgroups, red lineages are the San Salvador Island generalists, green lineages are the San Salvador Island molluscivores, dark blue lineages are the large-jawed scale-eaters and light blue lineages are the small jawed scale-eater. Percentages indicate the proportion of the Cyprinodon genome assigned to each topology. (TIFF) [file pgen.1006919.s002.tiff]

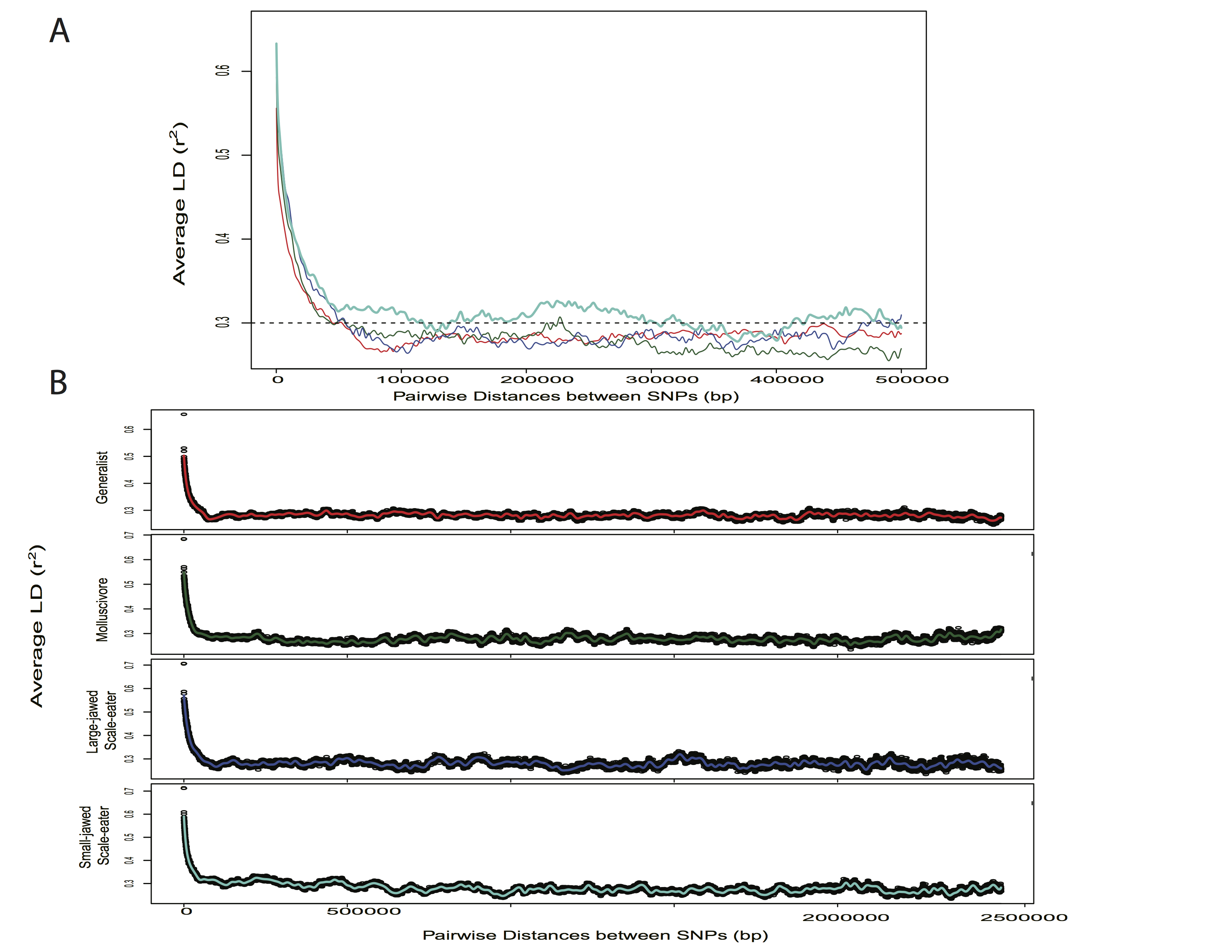

Supplement: S3 Fig — Average r2 values for pairwise SNPs A) within a distance of 500,000 bp of each other and B) across the entirety of the largest scaffold of the genome (KL652500.1, 4.2 Mb). r2 was calculated from 5 individuals of each of the San Salvador Island species: generalists (red), molluscivores (green), large-jawed scale-eaters (dark blue) and small-jawed scale-eaters (light blue). The black horizontal dashed line in panel A is arbitrarily set at r2 = 0.3 as a marker for comparing decay between the four groups. (TIFF) [file pgen.1006919.s003.tiff]

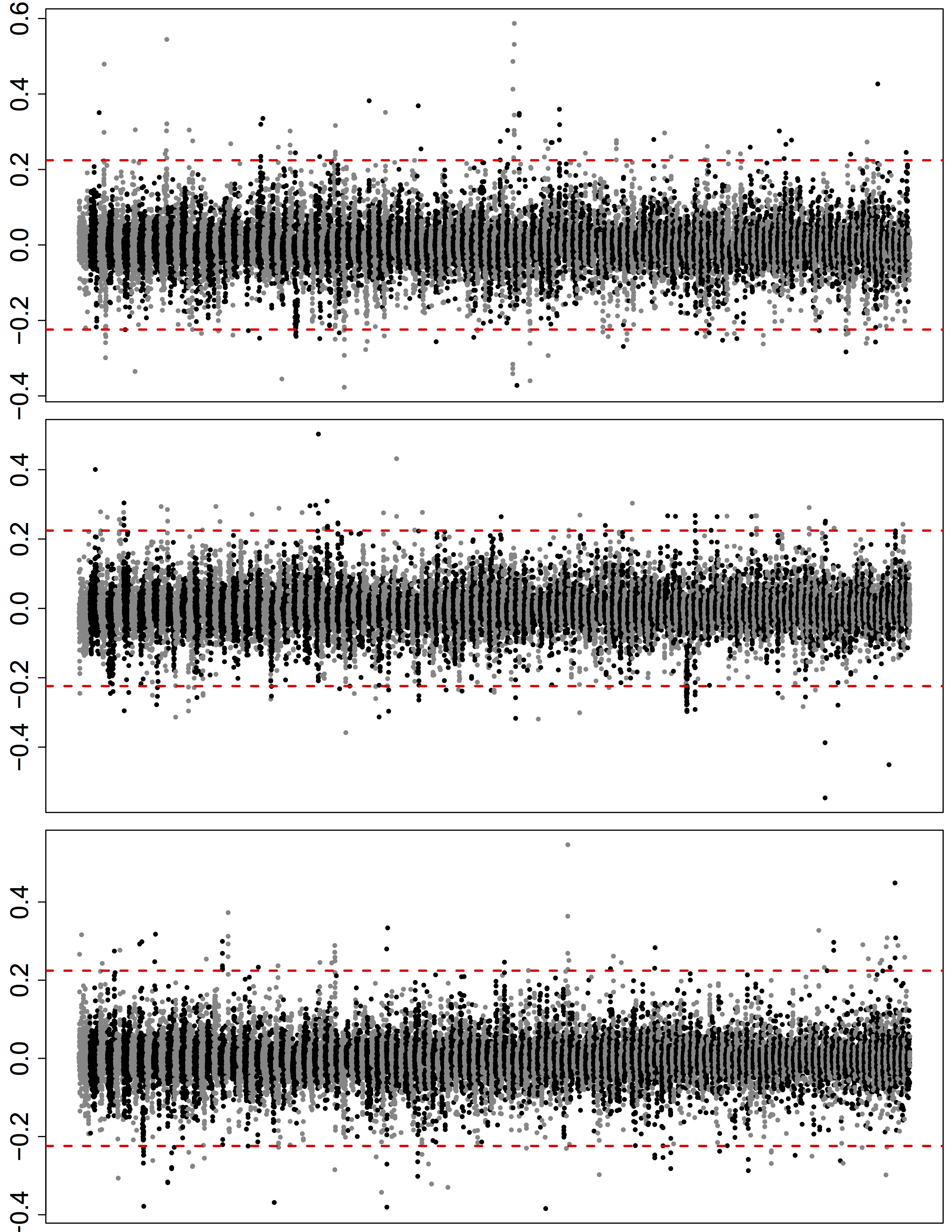

Supplement: S4 Fig — Manhattan plot of the f4 values between the San Salvador Island molluscivores, scale-eaters, C. laciniatus from New Providence Island, Bahamas and C. bondi from Etang Saumautre, Dominican Republic. Alternating gray/black colors indicate different scaffolds, starting with the largest scaffolds in the top row and the smallest scaffolds in the bottom row. Dotted red lines mark the permutation based two-tailed significance level threshold of 0.02. (TIFF) [file pgen.1006919.s004.tiff]

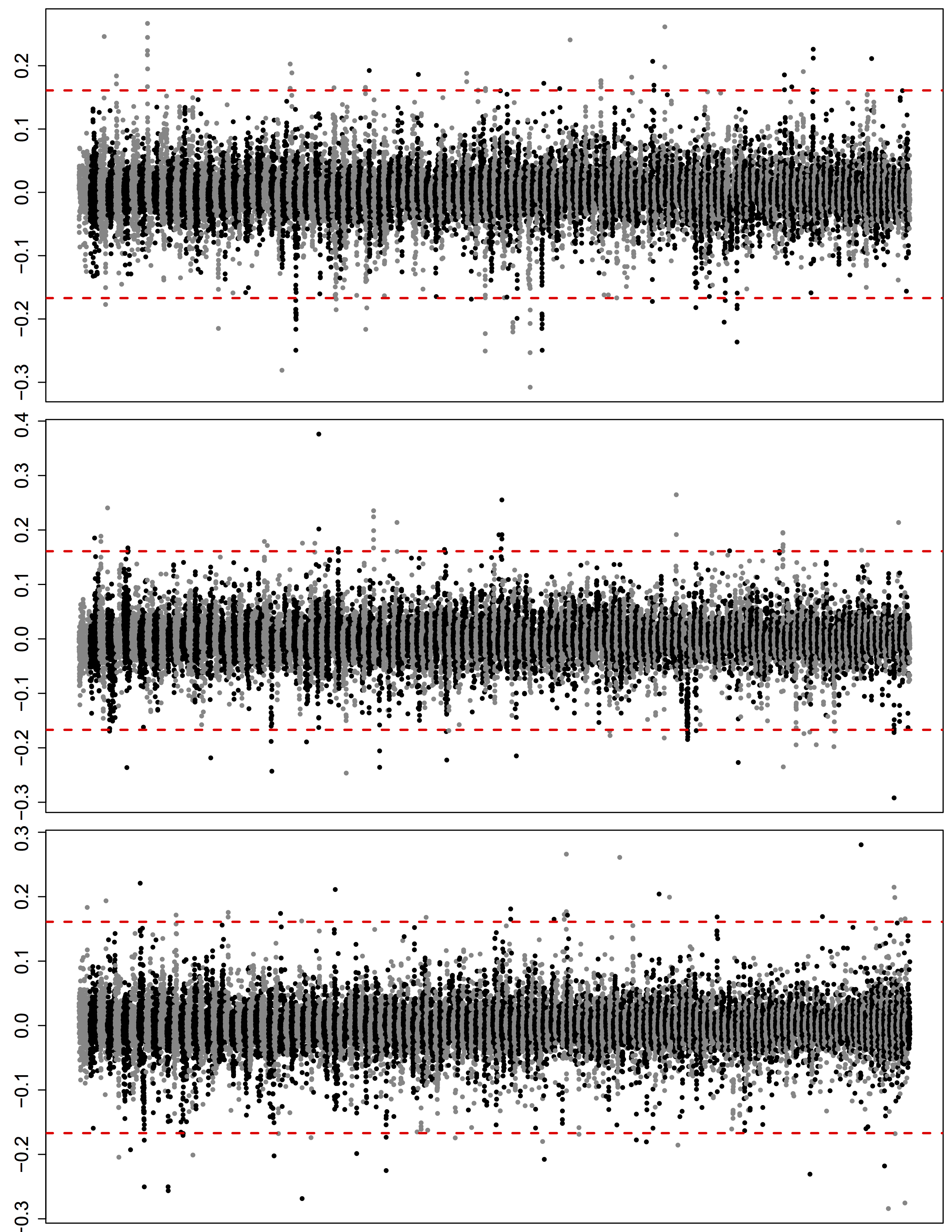

Supplement: S5 Fig — Manhattan plot of the f4 values between the San Salvador Island molluscivores, generalists, C. laciniatus from New Providence Island, Bahamas and C. bondi from Etang Saumatre, Dominican Republic. Alternating gray/black colors indicate different scaffolds, starting with the largest scaffolds in the top row and the smallest scaffolds in the bottom row. Dotted red lines mark the permutation based two-tailed significance level threshold of 0.02. (TIFF) [file pgen.1006919.s005.tiff]

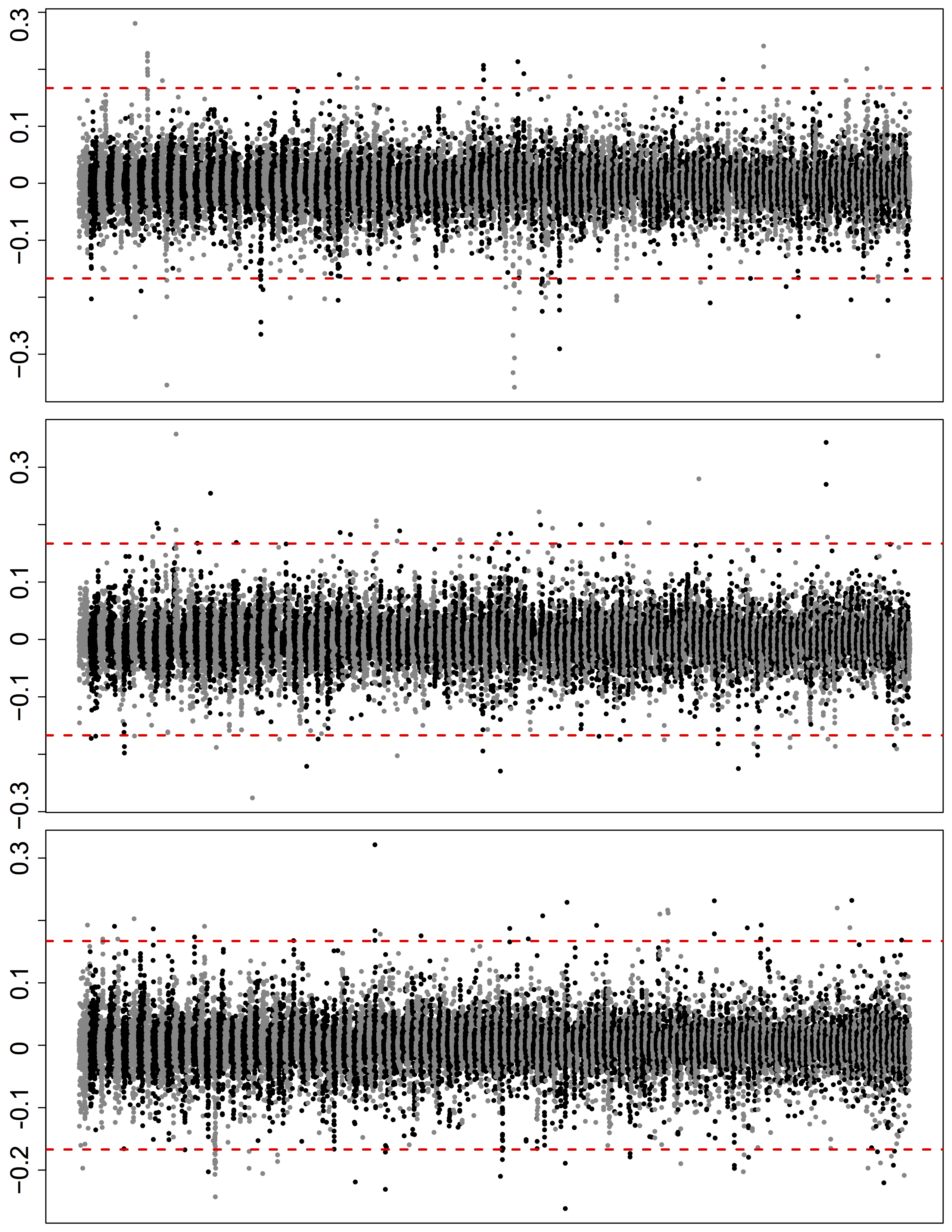

Supplement: S6 Fig — Manhattan plot of the f4 values between the San Salvador Island large-jawed scale-eaters, small-jawed scale-eaters, C. laciniatus from New Providence Island, Bahamas and C. bondi from Etang Saumautre, Dominican Republic. Alternating gray/black colors indicate different scaffolds, starting with the largest scaffolds in the top row and the smallest scaffolds in the bottom row. Dotted red lines mark the permutation based two-tailed significance level thresholds of 0.02. (TIFF) [file pgen.1006919.s006.tiff]

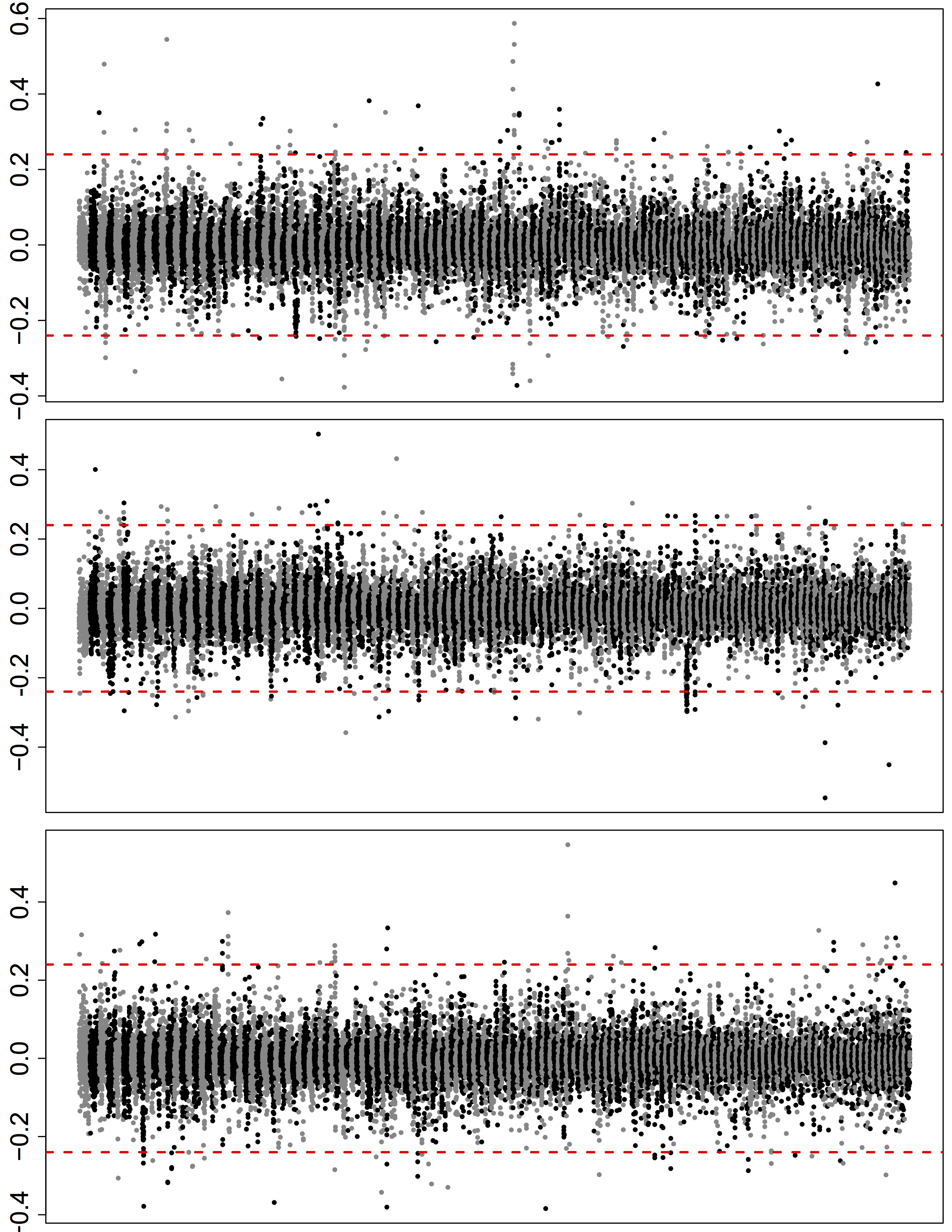

Supplement: S7 Fig — Manhattan plot of the f4 values between the San Salvador Island large-jawed scale-eaters, molluscivores, C. laciniatus from New Providence Island, Bahamas and C. bondi from Etang Saumatre, Dominican Republic. Alternating gray/black colors indicate different scaffolds, starting with the largest scaffolds in the top row and the smallest scaffolds in the bottom row. Dotted red lines mark the permutation based two-tailed significance level thresholds of 0.02. (TIFF) [file pgen.1006919.s007.tiff]

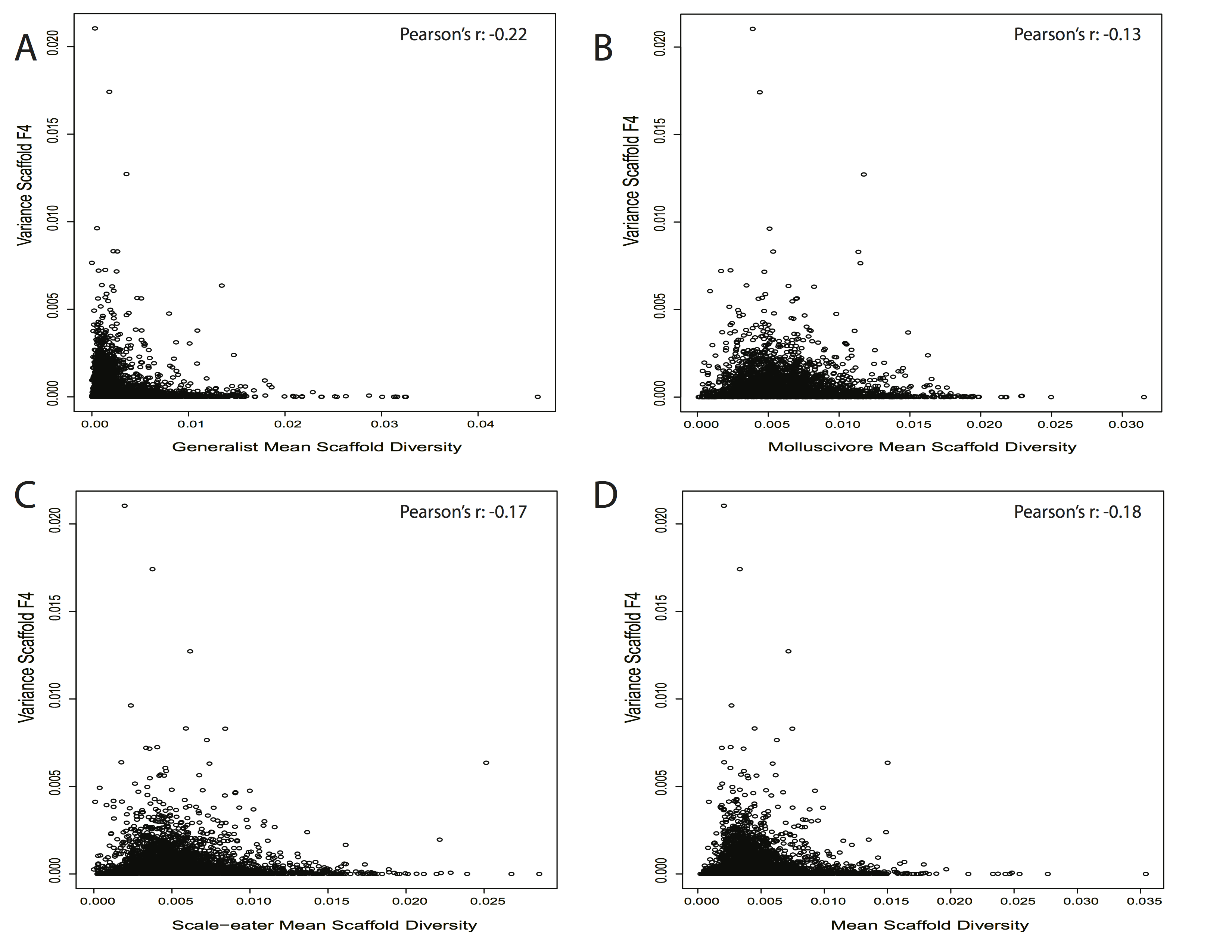

Supplement: S8 Fig — The variance in f4 statistic of a region compared to within-population diversity in A) molluscivores B) scale-eaters, and C) generalists, D) and average within-population diversity in all three species. (TIFF) [file pgen.1006919.s008.tiff]

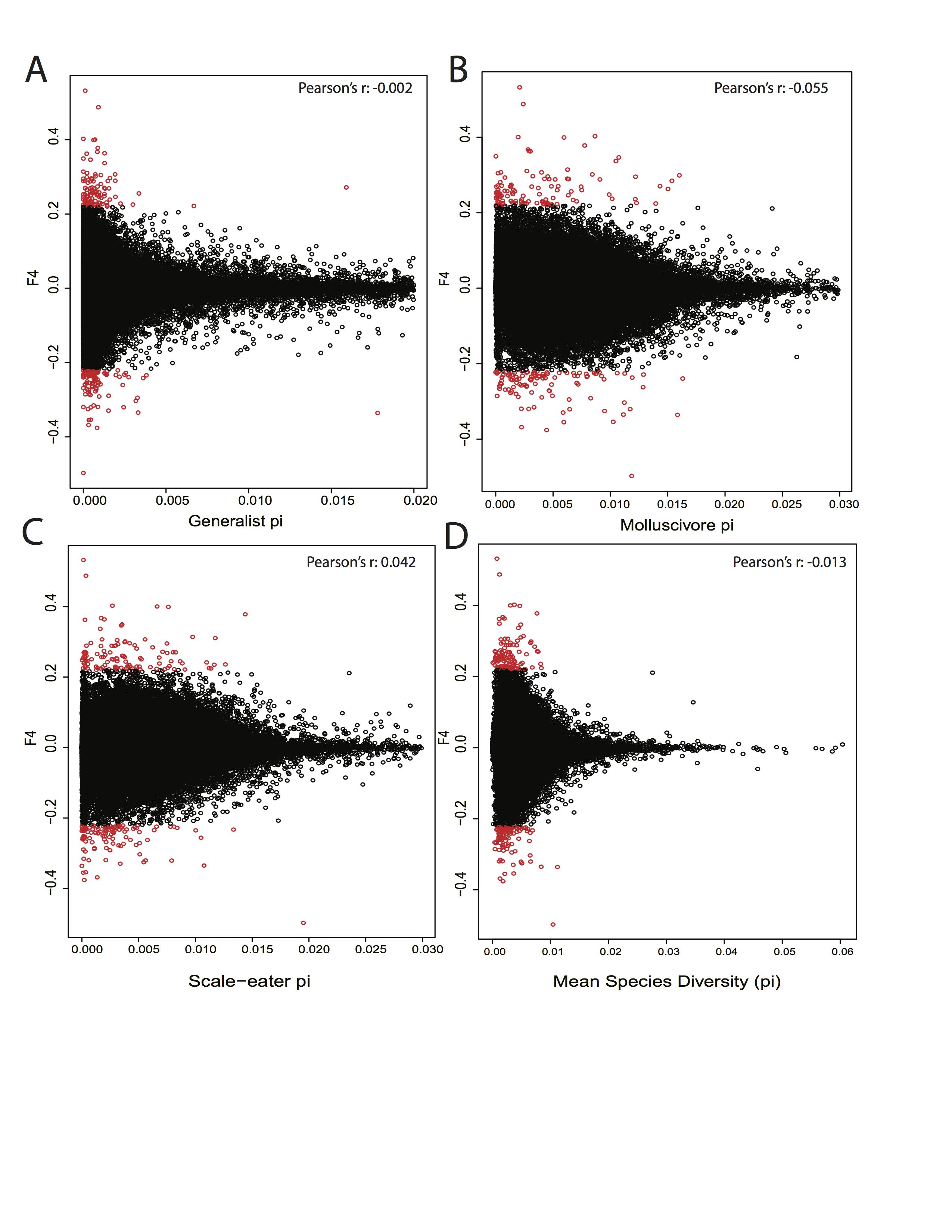

Supplement: S9 Fig — Red dots indicate 10-kb regions with signals of introgression above permutations based significance level. The f4 statistic of a region compared to within-population diversity in A) molluscivores and scale-eaters B) scale-eaters and generalists, and C) molluscivores and generalists D) and average within-population diversity in all three species. (TIFF) [file pgen.1006919.s009.tiff]

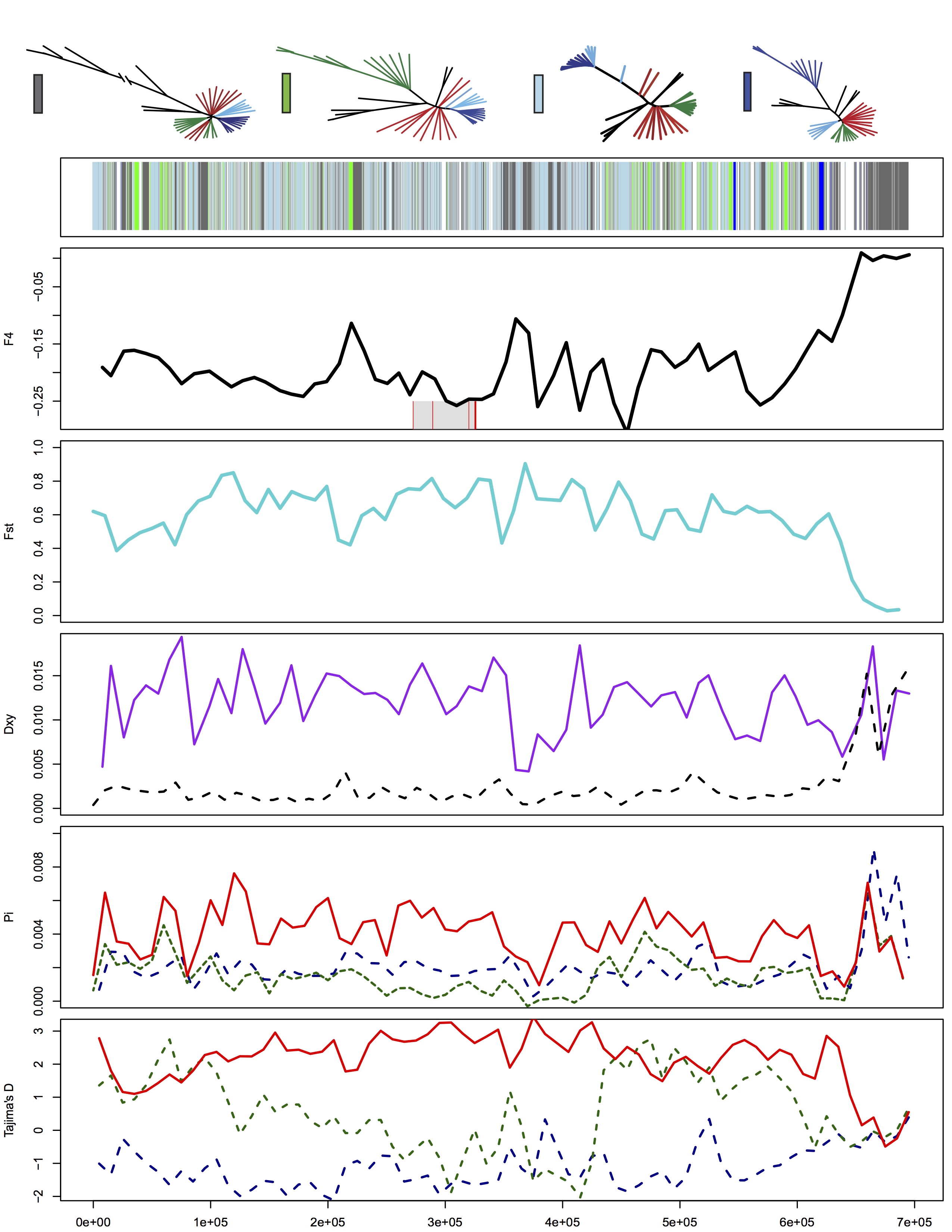

Supplement: S10 Fig — Row 1 shows the history assigned by SAGUARO to segments along a 700-kb scaffold (dark grey: dominant topology; blue: large-jawed scale-eater topology; light blue: combined scale-eater topology; green: molluscivore topology; light grey: all other topologies; white: unassigned segments). Row 2 shows average f4 value across non-overlapping 10-kb windows between molluscivores/scale-eaters. Shaded grey box shows region annotated for ski gene with exons in red. Row 3 shows average Fst value across non-overlapping 10-kb windows between molluscivores/scale-eaters (turquoise). Row 4 shows between-population divergence (Dxy) across non-overlapping 10-kb windows between molluscivores/scale-eaters and molluscivores/C. laciniatus (grey-dashed). Row 5 shows within-population diversity (π) across non-overlapping 10-kb windows (blue-dashed: scale-eater; green: molluscivore). Row 6 shows Tajima’s D across non-overlapping 10-kb windows (blue-dashed: scale-eater; green: molluscivore. (TIFF) [file pgen.1006919.s010.tiff]

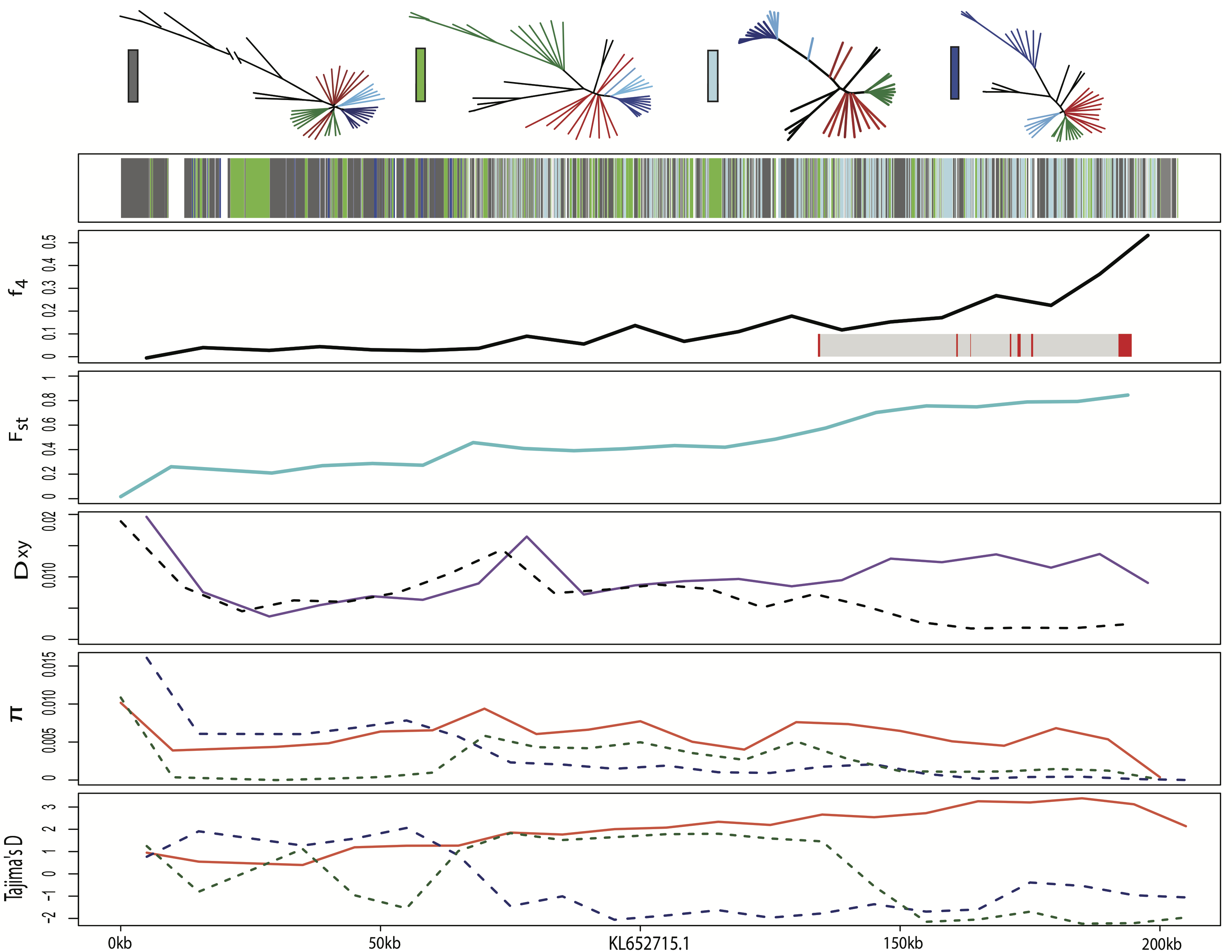

Supplement: S11 Fig — Fixed variants in this region were previously associated with pupfish oral jaw size [55]. Row 1 shows the history assigned by SAGUARO to segments along a 200-kb scaffold (dark grey: dominant topology; blue: large-jawed scale-eater topology; light blue: combined scale-eater topology; green: molluscivore topology; light grey: all other topologies; white: unassigned segments). Row 2 shows average f4 value across non-overlapping 10-kb windows between mollsucivores/scale-eaters. Shaded grey box shows region annotated for ski gene with exons in red. Row 3 shows average Fst value across non-overlapping 10-kb windows between molluscivores/scale-eaters (turquoise). Row 4 shows between-population divergence (Dxy) across non-overlapping 10-kb windows between molluscivores/scale-eaters and molluscivores/C. laciniatus (grey-dashed). Row 5 shows within-population diversity (π) across non-overlapping 10-kb windows (blue-dashed: scale-eater; green: molluscivore). Row 6 shows Tajima’s D across non-overlapping 10-kb windows (blue-dashed: scale-eater; green: molluscivore. (TIFF) [file pgen.1006919.s011.tiff]

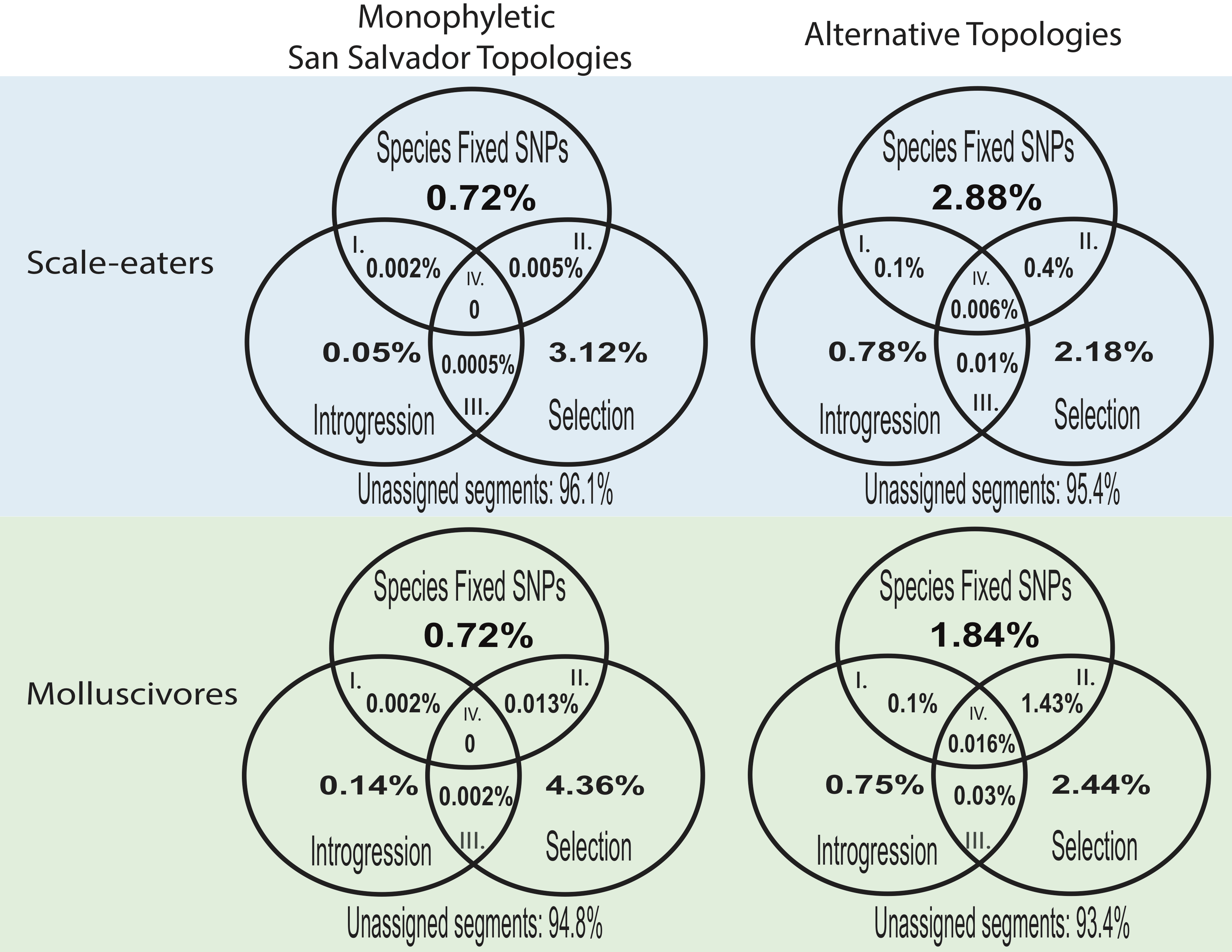

Supplement: S12 Fig — Venn diagrams of the contribution of different sources of genetic variation to speciation in this system based on the overlap of regions with fixed SNPs between the molluscivore and large-jawed scale-eater, significant f4 values of introgression, and Tajima’s D values below the simulation based lower one-tailed significance level of 0.02. Under each topology, we calculated the percentage of I) regions that contain introgressed genetic variation from the Caribbean contributing to species divergence, II) regions that have undergone strong selective sweeps from non-introgressed genetic variation on San Salvador Island, III) adaptively introgressed regions not contributing to species divergence, and IV) regions that have undergone selective sweeps of introgressed variation that contributed to species divergence of the two specialists (i.e. contain fixed SNPs between the specialists). The percentage of segments assigned to topologies, but not assigned to any of the above categories, are provided below the Venn diagrams. (TIFF) [file pgen.1006919.s012.tiff]

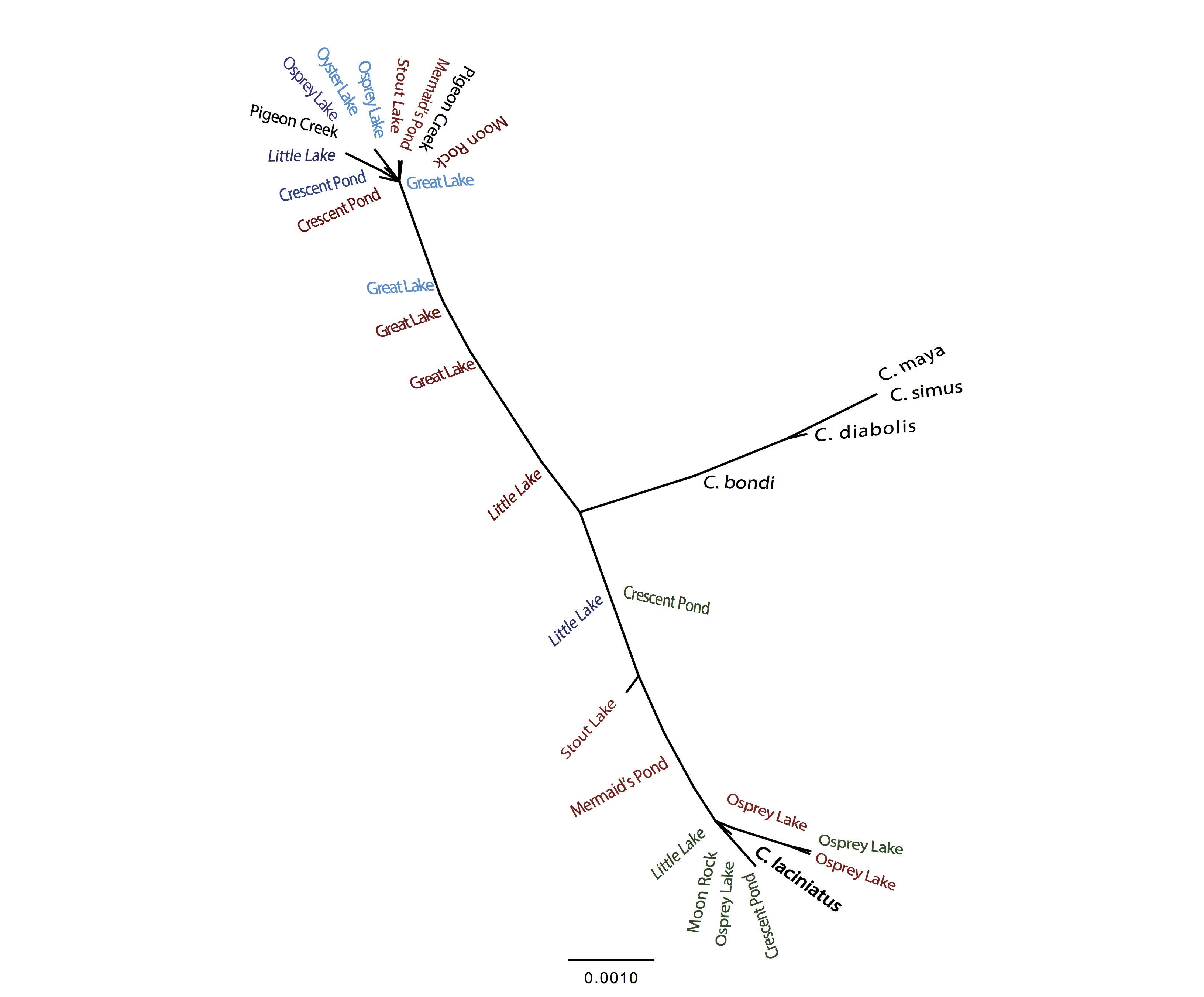

Supplement: S13 Fig — The names indicate the pond locality of the individuals (green: molluscivores; dark blue: large-jawed scale-eaters; light blue: small-jawed scale-eaters; black: pupfish outgroups). The scale bar indicates number of substitutions/bp. (TIFF) [file pgen.1006919.s013.tiff]

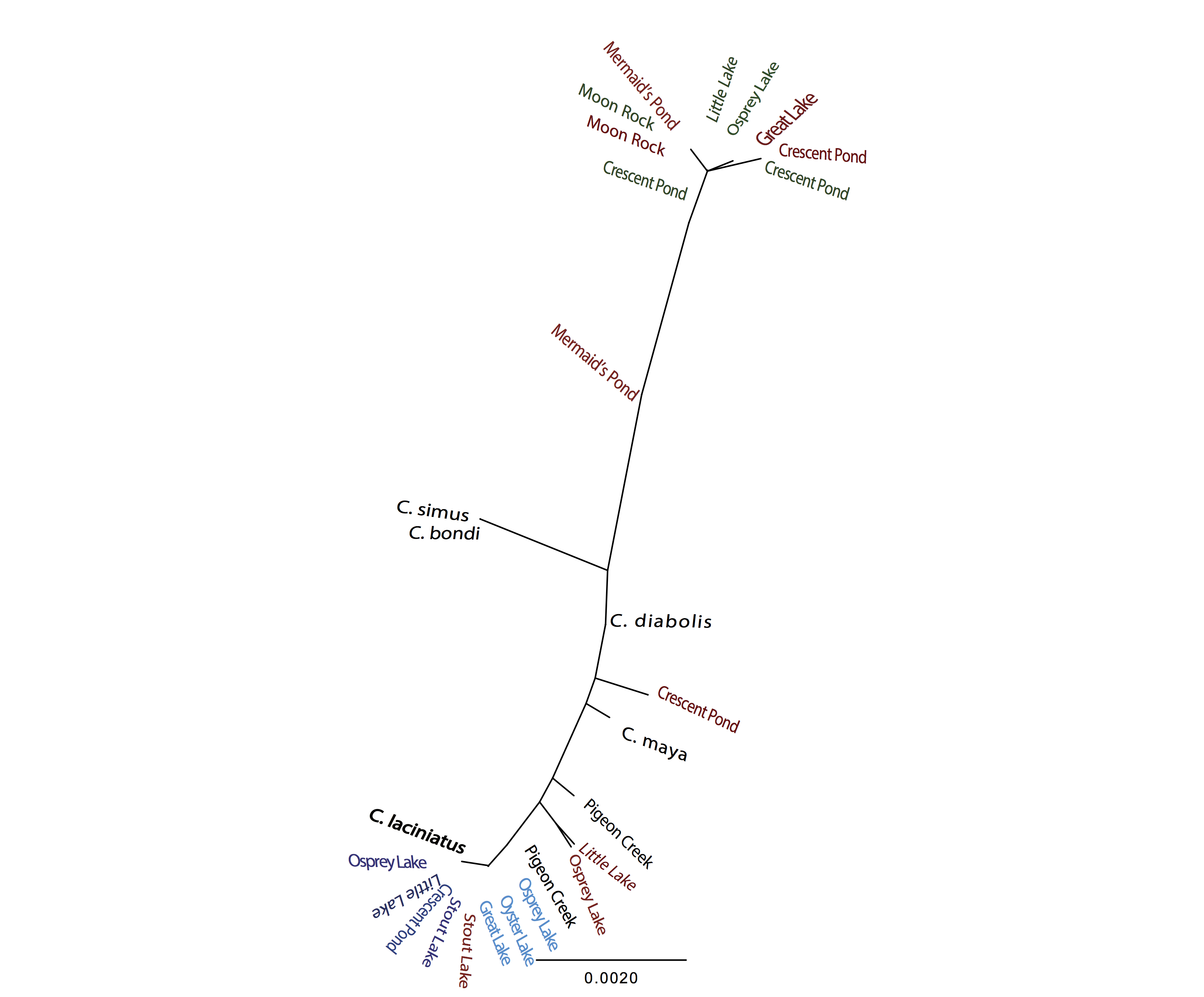

Supplement: S14 Fig — The names indicate the pond locality of the individuals (green: molluscivores; dark blue: large-jawed scale-eaters; light blue: small-jawed scale-eaters; black: pupfish outgroups). The scale bar indicates number of substitutions/bp. (TIFF) [file pgen.1006919.s014.tiff]

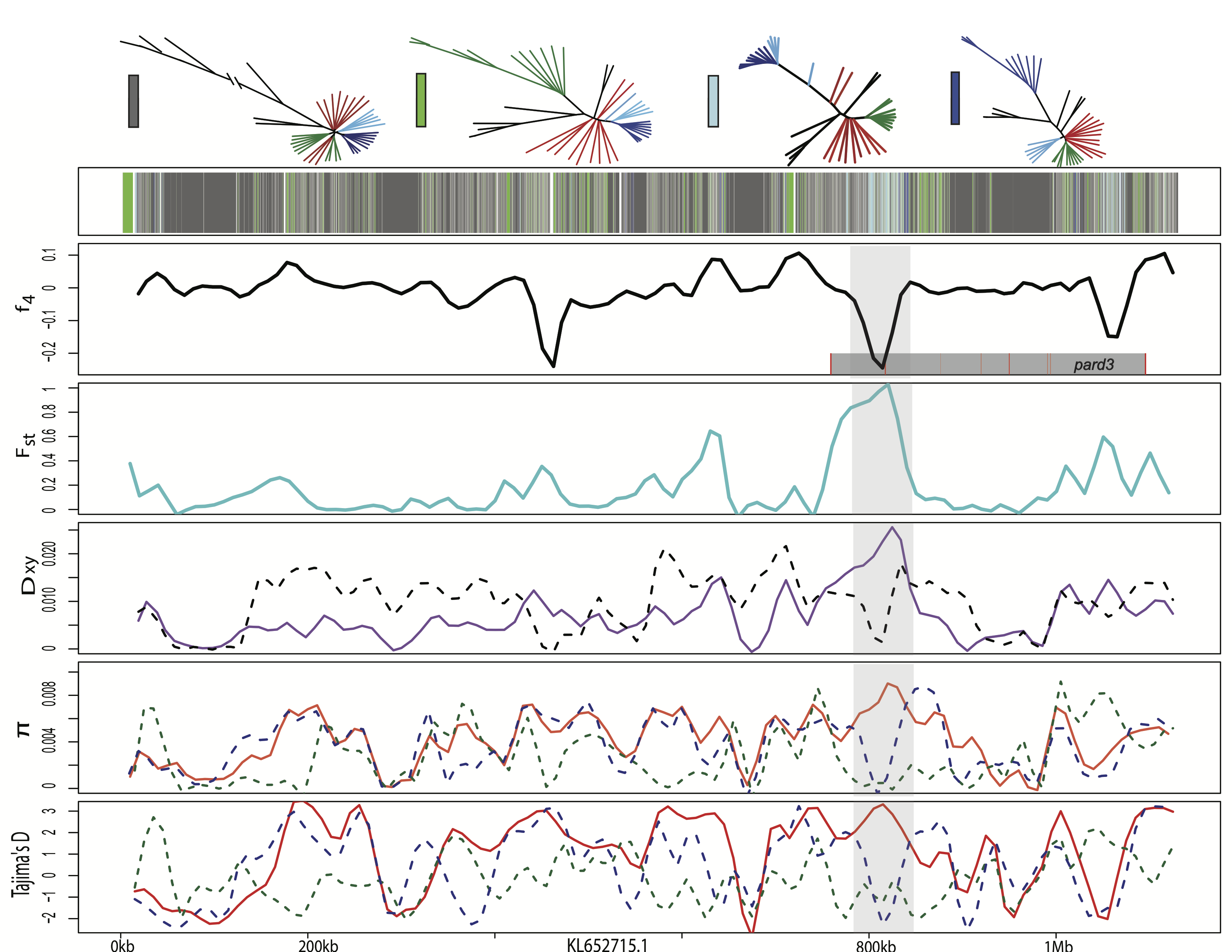

Supplement: S15 Fig — Fixed variants in this region were previously associated with pupfish oral jaw size [55]. Row 1 shows the history assigned by SAGUARO to segments along a 1-Mb scaffold (dark grey: dominant topology; blue: large-jawed scale-eater topology; light blue: combined scale-eater topology; green: molluscivore topology; light grey: all other topologies; white: unassigned segments). Row 2 shows average f4 value across non-overlapping 10-kb windows between mollsucivores/scale-eaters. Shaded grey box shows region annotated for pard3 gene with exons in red. Row 3 shows average Fst value across non-overlapping 10-kb windows between molluscivores/scale-eaters (turquoise). Row 4 shows between-population divergence (Dxy) across non-overlapping 10-kb windows between molluscivores/scale-eaters (purple) and scale-eaters/C. laciniatus (grey-dashed). Row 5 shows within-population diversity (π) across non-overlapping 10-kb windows (blue-dashed: scale-eater; green: molluscivore). Row 6 shows Tajima’s D across non-overlapping 10-kb windows (blue-dashed: scale-eater; green: molluscivore. (TIFF) [file pgen.1006919.s015.tiff]

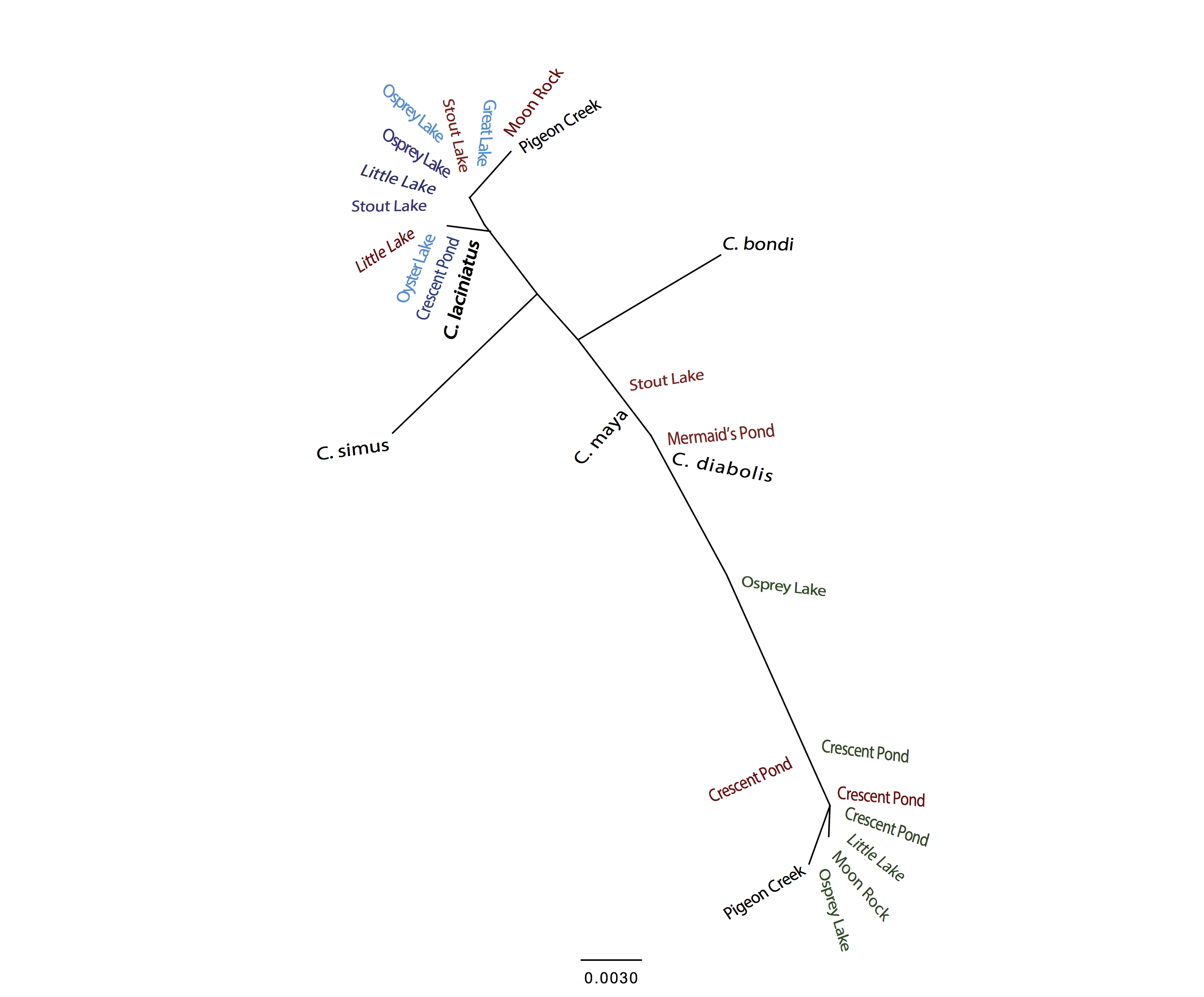

Supplement: S16 Fig — The names indicate the pond locality of the individuals (green: molluscivores; dark blue: large-jawed scale-eaters; light blue: small-jawed scale-eaters; black: pupfish outgroups). The scale bar indicates number of substitutions/bp. (TIFF) [file pgen.1006919.s016.tiff]

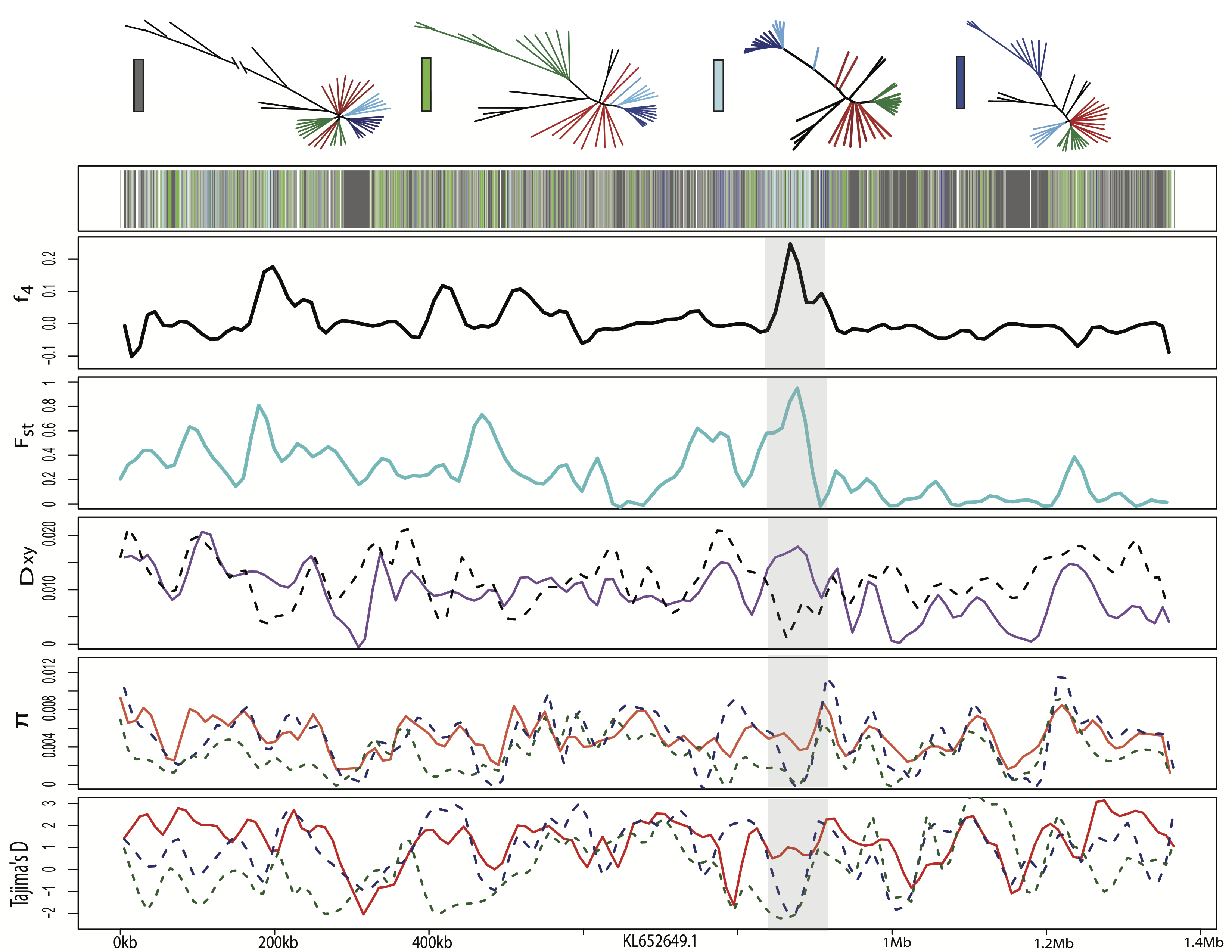

Supplement: S17 Fig — Fixed variants in this region were previously associated with pupfish oral jaw size [55]. Row 1 shows the history assigned by SAGUARO to segments along a 1.4-Mb scaffold (dark grey: dominant topology; blue: large-jawed scale-eater topology; light blue: combined scale-eater topology; green: molluscivore topology; light grey: all other topologies; white: unassigned segments). Row 2 shows average f4 value across non-overlapping 10-kb windows between mollsucivores/scale-eaters. Row 3 shows average Fst value across non-overlapping 10-kb windows between molluscivores/scale-eaters (turquoise). Row 4 shows between-population divergence (Dxy) across non-overlapping 10-kb windows between molluscivores/scale-eaters (purple) and molluscivores/C. laciniatus (grey-dashed). Row 5 shows within-population diversity (π) across non-overlapping 10-kb windows (blue-dashed: scale-eater; green: molluscivore). Row 6 shows Tajima’s D across non-overlapping 10-kb windows (blue-dashed: scale-eater; green: molluscivore. (TIFF) [file pgen.1006919.s017.tiff]

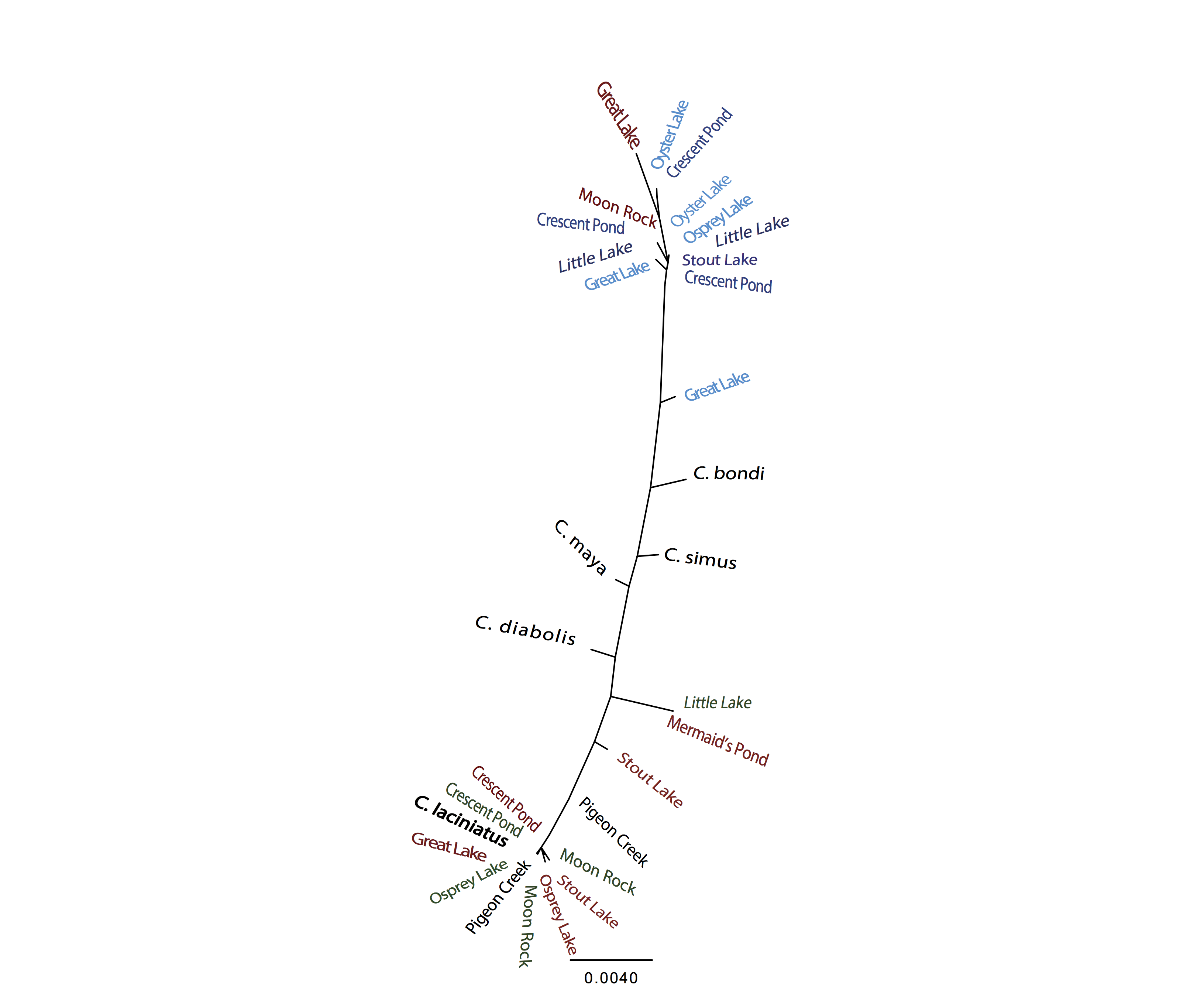

Supplement: S18 Fig — The names indicate the pond locality of the individuals (green: molluscivores; dark blue: large-jawed scale-eaters; light blue: small-jawed scale-eaters; black: pupfish outgroups). The scale bar indicates number of substitutions/bp. (TIFF) [file pgen.1006919.s018.tiff]

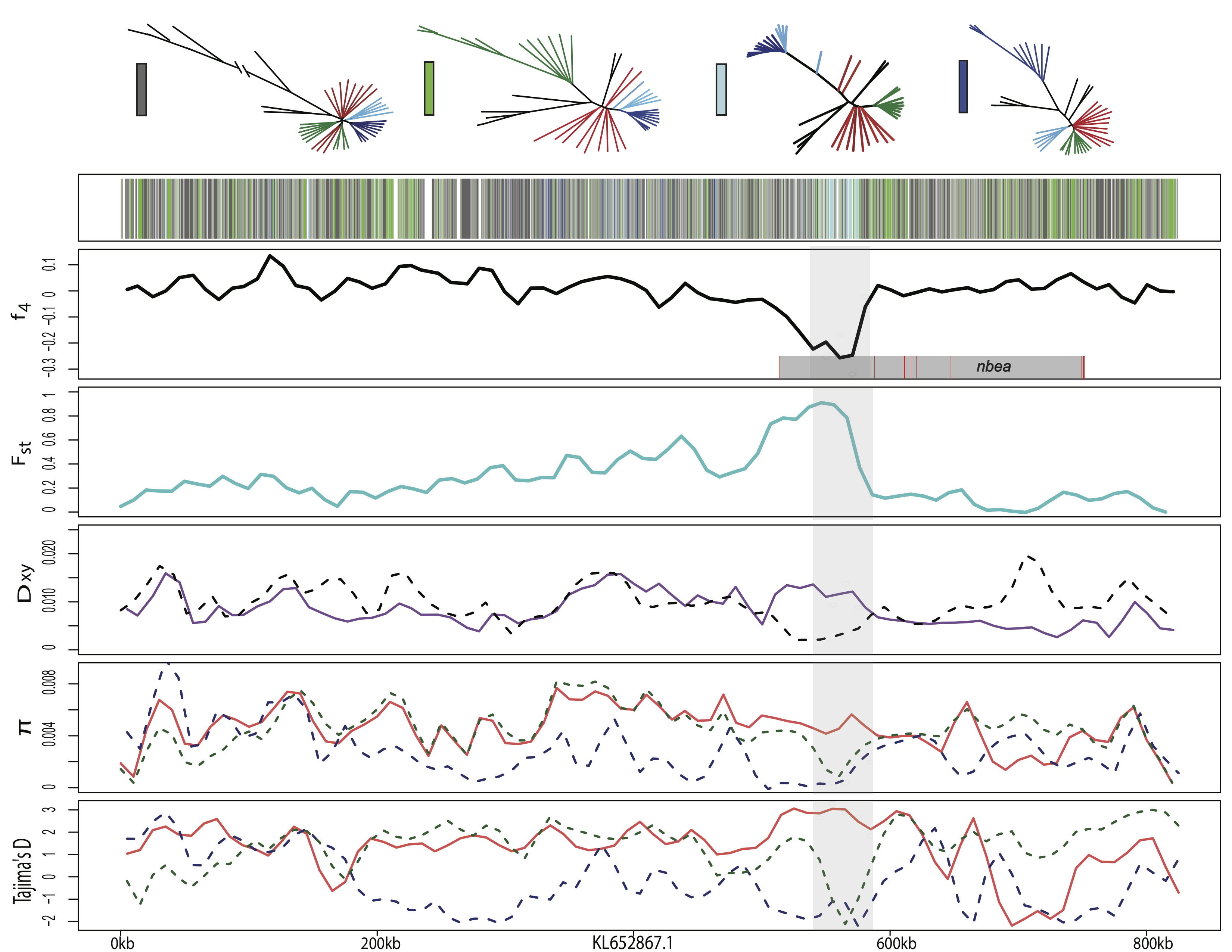

Supplement: S19 Fig — Row 1 shows the history assigned by SAGUARO to segments along an 800-kb scaffold (dark grey: dominant topology; blue: large-jawed scale-eater topology; light blue: combined scale-eater topology; green: molluscivore topology; light grey: all other topologies; white: unassigned segments). Row 2 shows average f4 value across non-overlapping 10-kb windows between mollsucivores/scale-eaters. Shaded grey box shows region annotated for nbea gene with exons in red. Row 3 shows average Fst value across non-overlapping 10-kb windows between molluscivores/scale-eaters (turquoise). Row 4 shows between-population divergence (Dxy) across non-overlapping 10-kb windows between molluscivores/scale-eaters (purple) and scale-eaters/C. laciniatus (grey-dashed). Row 5 shows within-population diversity (π) across non-overlapping 10-kb windows (blue-dashed: scale-eater; green: molluscivore). Row 6 shows Tajima’s D across non-overlapping 10-kb windows (blue-dashed: scale-eater; green: molluscivore. (TIFF) [file pgen.1006919.s019.tiff]

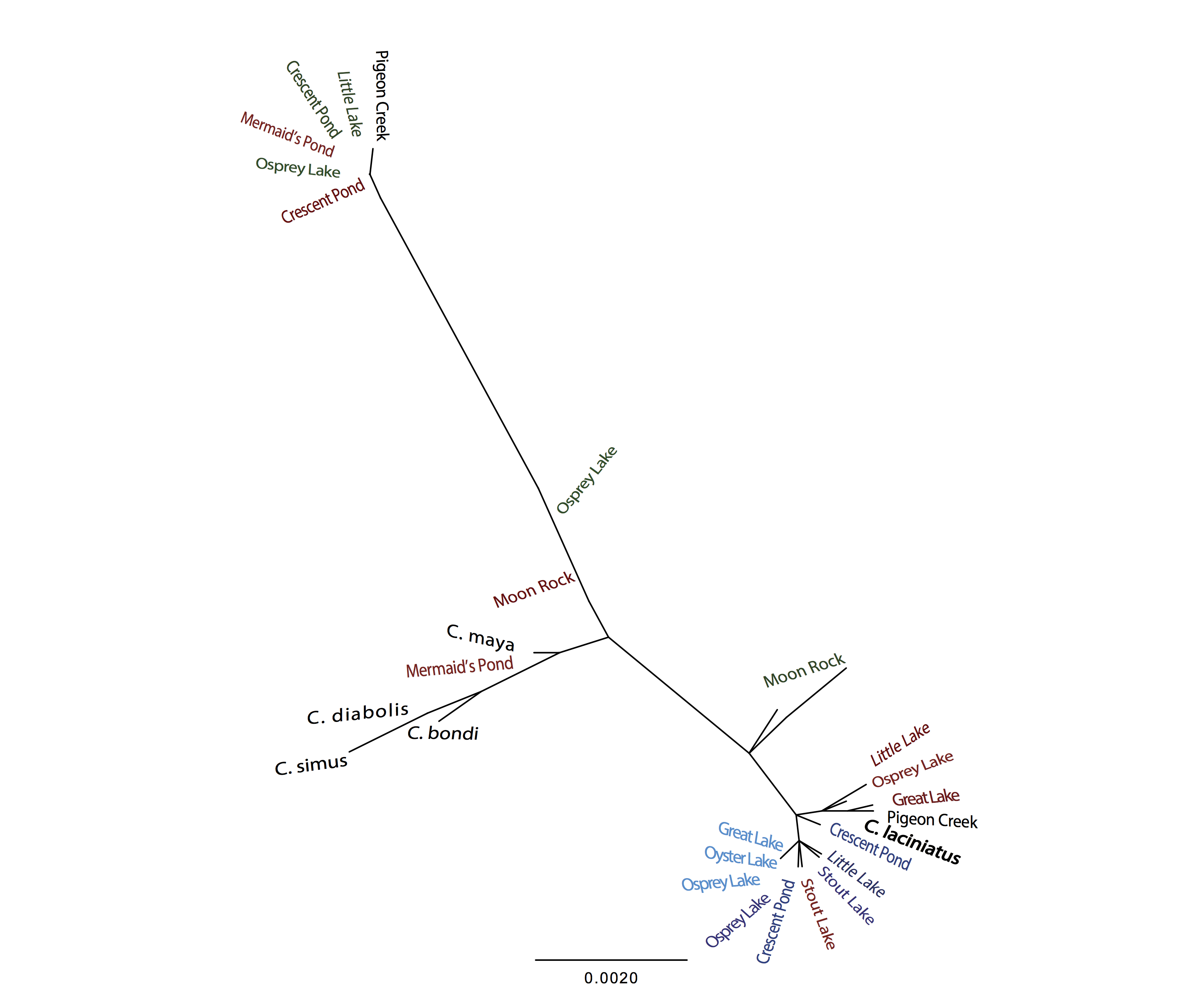

Supplement: S20 Fig — The names indicate the pond locality of the individuals (green: molluscivores; dark blue: large-jawed scale-eaters; light blue: small-jawed scale-eaters; black: pupfish outgroups). The scale bar indicates number of substitutions/bp. (TIFF) [file pgen.1006919.s020.tiff]

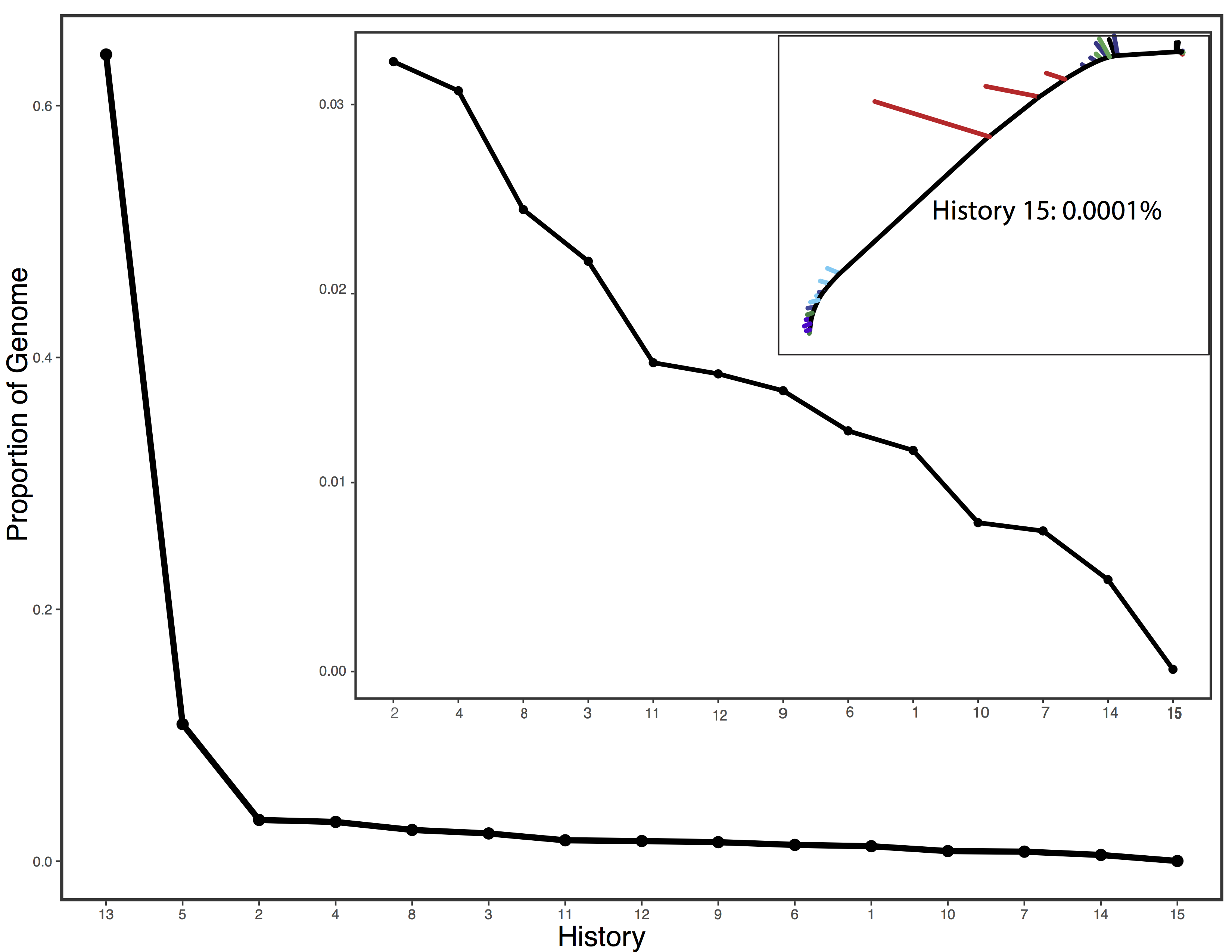

Supplement: S21 Fig — The insert is a closer look at the 13 topologies assigned to the smallest proportion of the genome and the largely uninformative 15th topology. This suggests saturation in the variance explained by topologies at 14. (TIFF) [file pgen.1006919.s021.tiff]

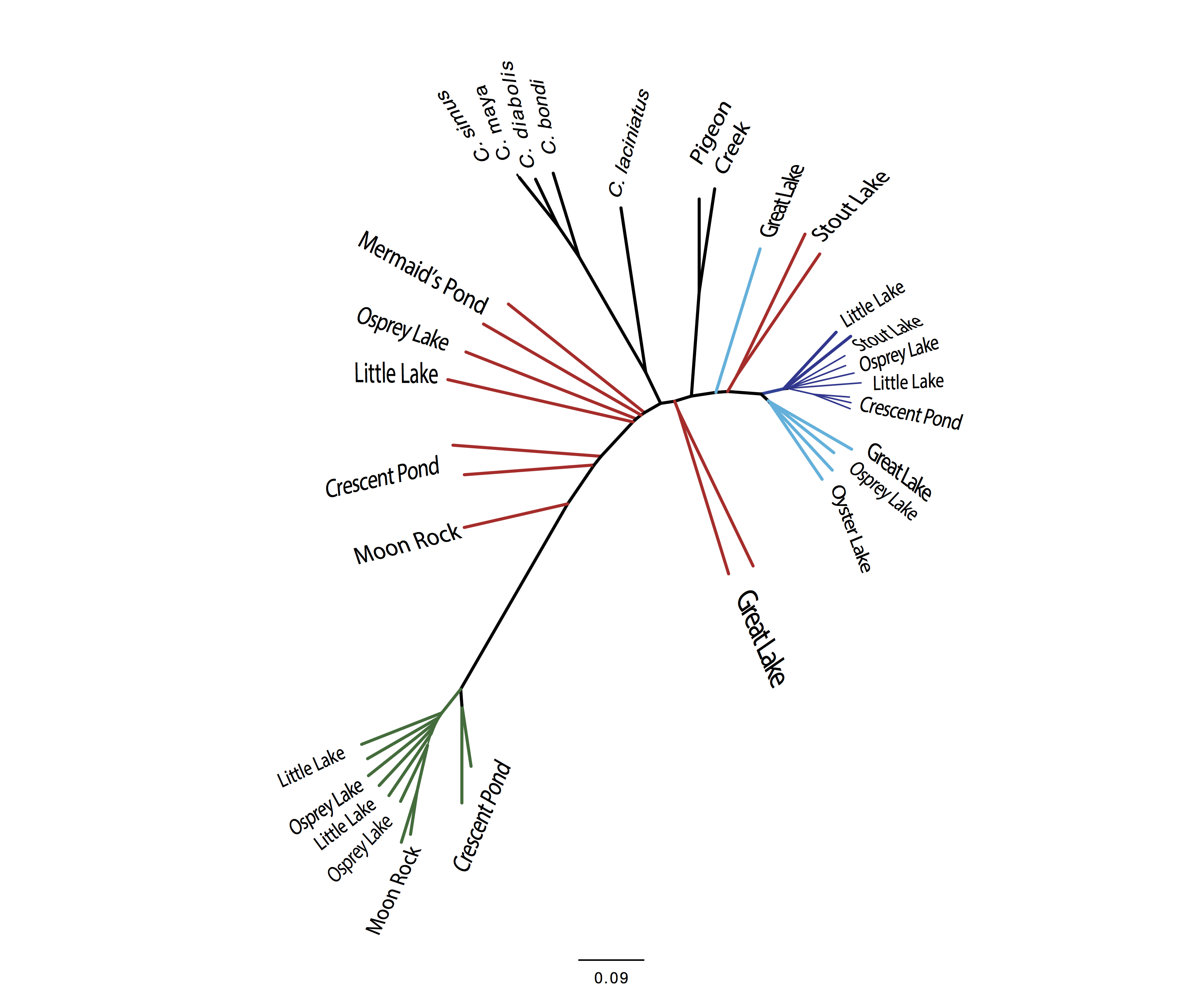

Supplement: S22 Fig — Black lineages are the Cyprinodon outgroups, red lineages are the San Salvador Island generalists, green lineages are the San Salvador Island molluscivores, dark blue lineages are the large jawed scale-eaters and light blue lineages are the small jawed scale-eater. This topology differs from the molluscivore topology created from unmasked genomic dataset (Fig 2A) in that along with the molluscivores, generalists from Mermaid’s Pond, Osprey Lake, Little Lake, Crescent Pond, and Moon Rock Pond appear more closely related to outgroup populations than other San Salvador Island populations. (TIFF) [file pgen.1006919.s022.tiff]

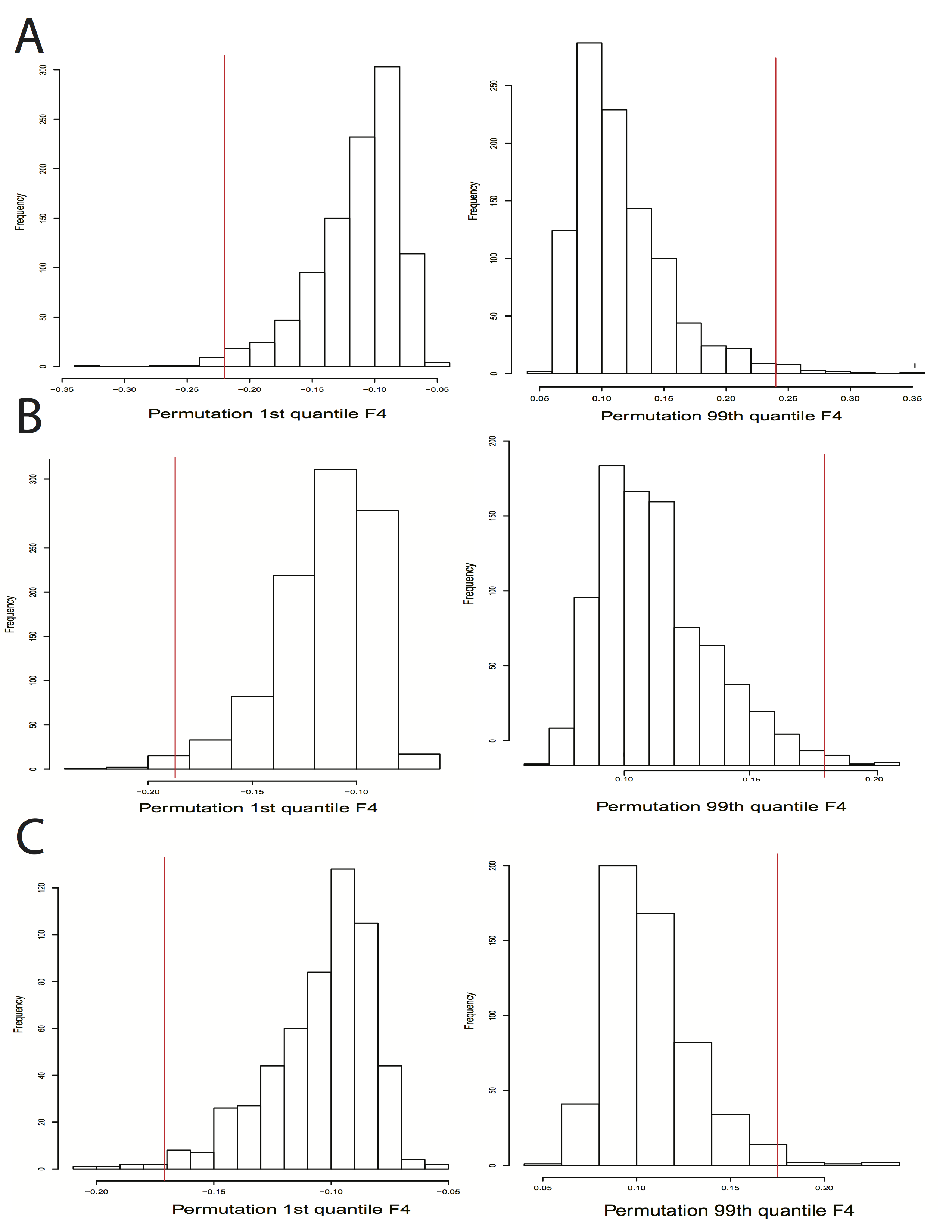

Supplement: S23 Fig — The red lines represent the 1st quantile (left panels) and 99th (right panels) observed f4 values with less than 1% chance of being in the null permutation based distributions of the f4 test combinations including a) molluscivores and scale-eaters, b) molluscivores and generalists, and c) scale-eaters and generalists. (TIFF) [file pgen.1006919.s023.tiff]
