# Supplementary material for: Adaptive introgression from distant Caribbean islands contributed to the diversification of a microendemic adaptive radiation of trophic specialist pupfishes
Source: PLoS Genet. 2017 Aug 10;13(8):e1006919. doi: 10.1371/journal.pgen.1006919 (PMC5552031; doi:10.1371/journal.pgen.1006919)
Supplement: S1 Table — (DOCX) [file pgen.1006919.s025.docx]

**S1 Table. Hypothesized topologies from the SAGUARO analysis.**

|  |  | **Cumulative length (bp)** | | **Percent** |
| --- | --- | --- | --- | --- |
| **Monophyletic** | |  | | **82.6** |
|  | Dominant | 580,033,357 | | 64.06 |
|  | History 5 | 98,537,488 | | 10.89 |
|  | History 0 | 42,238,737 | | 4.67 |
|  | History 6 | 11,706,448 | | 1.29 |
|  | History 1 | 10,761,277 | | 1.19 |
|  | History 14 | 4,479,983 | | 0.5 |
| **Non-monophyletic** | |  | | **17.4** |
|  | Large jawed scale-eater | | 29,638,216 | 3.27 |
|  | Molluscivore | 28,221,917 | | 3.12 |
|  | History 8 | 22,451,018 | | 2.48 |
|  | History 3 | 19,942,360 | | 2.2 |
|  | Scale-eater | 15,017,585 | | 1.66 |
|  | History 12 | 14,475,837 | | 1.6 |
|  | History 9 | 13,655,853 | | 1.51 |
|  | History 10 | 7,250,529 | | 0.8 |
|  | History 7 | 6,848,171 | | 0.76 |
| **Total** |  | **905,258,776** | | **100** |
