# Supplementary material for: Adaptive introgression from distant Caribbean islands contributed to the diversification of a microendemic adaptive radiation of trophic specialist pupfishes
Source: PLoS Genet. 2017 Aug 10;13(8):e1006919. doi: 10.1371/journal.pgen.1006919 (PMC5552031; doi:10.1371/journal.pgen.1006919)
Supplement: S2 Table — Ancestry in small-jawed scale-eaters is assigned for SNPs fixed (n = 1,887) between the large-jawed scale-eaters and generalists. The genotype count in the 5 small-jawed scale-eater individuals at SNPs fixed between the large-jawed scale-eaters and generalists that are homozygous for one of the parental genotypes or heterozygous between the two (the proportion of loci in is provided in the parentheses; n = 1,887). The observed proportion of ancestry in small-jawed scale-eaters does not fit the proportions expected for F1 hybrids (all heterozygotes), backcross with the one of the parental species (half heterozgyous, half homozygous for parental allele), or F2 hybrids (half heterozygous, one-fourth homozygous for large-jawed scale-eater, and one-fourth homozygous for generalist). X2 value and P-value are provided for the X2 goodness-of-fit test of the observed proportions of ancestry across the genome to those expected of F2 hybrids. (DOCX) [file pgen.1006919.s026.docx]

**S2 Table. Ancestry proportions expected of small-jawed scale-eaters if they represent hybrids of the large-jawed scale-eaters and generalists.** Ancestry in small-jawed scale-eaters is assigned for SNPs fixed (n=1,887) between the large-jawed scale-eaters and generalists. The genotype count in the 5 small-jawed scale-eater individuals at SNPs fixed between the large-jawed scale-eaters and generalists that are homozygous for one of the parental genotypes or heterozygous between the two (the proportion of loci in is provided in the parentheses). The observed proportion of ancestry in small-jawed scale-eaters does not fit the proportions expected for F_1_ hybrids (all heterozygotes), backcross with the one of the parental species (half heterozgyous, half homozygous for parental allele), or F_2_ hybrids (half heterzygous, one-fourth homozygous for large-jawed scale-eater, and one-fourth homozygous for small-jawed scale-eater). *X*^2^ value and P-value are provided for the *X*^2^ goodness-of-fit test of the observed proportions of ancestry across the genome to those expected of F_2_ hybrids.

| Individual | Homozygous Large-jawed Scale-eater | Heterozygous | Homozygous Generalist | F_2_ hybrid *χ*^2^ | F_2_ hybrid *χ*^2^ P-value |
| --- | --- | --- | --- | --- | --- |
| GREP1 | 785 (0.42) | 227 (0.15) | 825 (0.43) | 538.28 | 1.3 x 10^-117^ |
| GREP2 | 261 (0.13) | 514 (0.27) | 1129 (0.59) | 446.01 | 1.41 x 10^-97^ |
| OSPP2 | 625 (0.33) | 423 (0.22) | 839 (0.45) | 332.57 | 9.01x10^-71^ |
| OYSP1 | 729 (0.39) | 300 (0.16) | 858 (0.45) | 500.31 | 2.28x10^-109^ |
| OYSP3 | 863 (0.46) | 228 (0.12) | 796 (0.42) | 634.61 | 1.56x10^-138^ |
| Average | 652.5 (0.35) | 348.4 (0.18) | 886.9 (0.47) | 429.6 | 5.16x10^-94^ |
