# Supplementary material for: Adaptive introgression from distant Caribbean islands contributed to the diversification of a microendemic adaptive radiation of trophic specialist pupfishes
Source: PLoS Genet. 2017 Aug 10;13(8):e1006919. doi: 10.1371/journal.pgen.1006919 (PMC5552031; doi:10.1371/journal.pgen.1006919)
Supplement: S3 Table — Adaptively introgressed regions and gene annotations for fixed SNPs between scale-eater and molluscivore species that lie in genomic regions assigned to one of the three alternative topologies. Asterisks (*) indicate SNPs in gene regions associated with San Salvador Island pupfish oral jaw size variation in a previous study [55]. Bolded genes have known functional effects on craniofacial traits in a model system. Regions that are not annotated for genes are indicated with a dash (-). P-values indicate the number of permutations of the candidate region with f4 values greater than or equal to the observed f4 value. The number of fixed SNPs that were in coding positions of a gene are provide in parentheses after the total number of fixed SNPs in the candidate adaptive introgression region. The specialist(s) with a selective sweep detected in the 98th percentile of SweeD composite likelihood ratio test. (DOCX) [file pgen.1006919.s027.docx]

**S3 Table. 11 candidate adaptive introgression regions in San Salvador specialists.** Adaptively introgressed regions and gene annotations for fixed SNPs between scale-eater and molluscivore species that lie in genomic regions assigned to one of the three alternative topologies. Asterisks (*) indicate SNPs in gene regions associated with San Salvador pupfish oral jaw size variation in a previous study [55]. Bolded genes have known functional effects on craniofacial traits in a model system. Regions that are not annotated for genes are indicated with a dash (-). *P*-values indicate the number of permutations of the candidate region with *f_4_* values greater than or equal to the observed *f_4_* value. The number of fixed SNPs that were in coding positions of a gene are provide in parentheses after the total number of fixed SNPs in the candidate adaptive introgression region. The specialist(s) with a selective sweep detected in the 98^th^ percentile of SweeD composite likelihood ratio test.

|  |  |  |  |  |  |  |  | Generalist | | Molluscivore | | Scale-eater | | |
| --- | --- | --- | --- | --- | --- | --- | --- | --- | --- | --- | --- | --- | --- | --- |
| Scaffold | Segment | Gene | *f_4_* | *P*-value | Sweep | Fixed SNPs | Avg SNP Coverage | Tajima's D | pi | Tajima's D | pi | Tajima's D | pi | |
| KL652649.1 | 863668-873661 | NA* | 0.2536 | 0 | scale-eaters | 14(-) | 5.57+0.34 | 0.86 | 0.006 | -2.48 | 0.0011 | -2.04 | 0.00024 | |
| KL652702.1 | 312277-322263 | celf4 | 0.2461 | 0.001 | scale-eaters | 27(0) | 6.36+0.53 | 2.72 | 0.0042 | -2.29 | 0.00045 | -1.54 | 0.000032 | |
| KL652715.1 | 799363-809363 | pard3* | -0.223 | 0 | scale-eaters | 57(0) | 5.55+0.63 | 2.95 | 0.0065 | -2.18 | 0.0056 | -0.68 | 0.00022 | |
| KL652867.1 | 545190-575190 | nbea | -0.28 | 0 | molluscivores | 40(0) | 6.02+0.39 | 2.63 | 0.0043 | -0.86 | 0.000093 | -2.18 | 0.00036 | |
| KL652964.1 | 411177-421153 | **rbms3** | -0.2735 | 0.001 | scale-eaters | 1(0) | 5.21 | 2.95 | 0.0041 | -1.08 | 0.0011 | -2.19 | 0.0000098 | |
| KL652983.1 | 266059-276054 | **ski*** | 0.2606 | 0 | molluscivores | 3(1) | 6.19+0.11 | 2.22 | 0.0046 | -2.24 | 0.000082 | -1.18 | 0.0016 | |
| KL653033.1 | 403145-413142 | NA | -0.2798 | 0 | molluscivores/  scale-eaters | 1(-) | 6.05 | 2.09 | 0.0055 | -1.96 | 0.001 | -0.81 | 0.0038 | |
| KL653171.1 | 362672-372487 | ltbp2 | -0.2546 | 0 | molluscivores | 2(0) | 4.88+0.17 | 0.52 | 0.0055 | -2.02 | 0.0022 | 0.49 | 0.0038 | |
| KL653356.1 | 50344-70348 | srbd1 | 0.26 | 0.001 | molluscivores/  scale-eaters | 19(0) | 5.93+0.68 | 3.25 | 0.006 | -2.11 | 0.0003 | -1.36 | 0.00018 | |
| KL653356.1 | 70356-80348 | srbd1 | -0.2666 | 0 | molluscivores/  scale-eaters | 20(0) | 5.92+0.59 | 2.38 | 0.0056 | -1.98 | 0.000052 | -0.167 | 0.00027 | |
| KL653906.1 | 10377-20368 | mcu | -0.2275 | 0 | molluscivores | 7(0) | 6.01+0.96 | 0.46 | 0.0034 | -2.19 | 0.00087 | -1.66 | 0.0017 |  |
| Introgressed regions removed from candidates | | | | | | | | | | | | | | |
| KL653706.1 | 183622-203517 | plekhg* | 0.43 | 0.002 | molluscivores | 41(0) | 6.04+0.69 | 2.13 | 0.00035 | -1.96 | 0.000066 | -1.05 | 0.000011 |  |
| KL652959.1 | 265592-315719 | wnt7b | -0.24 | 0.004 | molluscivores/  scale-eaters | 28(0) | 5.75+0.78 | 2.64 | 0.001 | -1.16 | 0.0026 | -1.94 | 0.0057 |  |
