# Supplementary material for: Adaptive introgression from distant Caribbean islands contributed to the diversification of a microendemic adaptive radiation of trophic specialist pupfishes
Source: PLoS Genet. 2017 Aug 10;13(8):e1006919. doi: 10.1371/journal.pgen.1006919 (PMC5552031; doi:10.1371/journal.pgen.1006919)
Supplement: S4 Table — NA*(2649) is the unannotated candidate adaptive introgression region on scaffold KL652649.1 and NA (3033) is the unannotated candidate adaptive introgression region on scaffold KL653033.1. The two species with the lowest Dxy are bolded for each region. (DOCX) [file pgen.1006919.s028.docx]

**S4 Table. Pairwise genetic divergence (Dxy) between molluscivores, scale-eaters, Lake Cunningham, New Providence Island (*C. laciniatus*) and Etang Saumatre, Dominican Republic (*C. bondi*).** NA*(2649) is the unannotated candidate adaptive introgression region on scaffold KL652649.1 and NA (3033) is the unannotated candidate adaptive introgression region on scaffold KL653033.1. The two species with the lowest Dxy are bolded for each region.

| Comparison | ski | pard3 | plekhg | NA*(2649) | rbms3 | nbea | wnt7b | celf4 | ltbp2 | srbd1 (+f4) | srbd1 (-f4) | mcu | NA (3033) |
| --- | --- | --- | --- | --- | --- | --- | --- | --- | --- | --- | --- | --- | --- |
| scale-eater v.  molluscivore | 0.013 | 0.017 | 0.014 | 0.019 | 0.013 | 0.012 | 0.014 | 0.1 | 0.01 | 0.0014 | 0.015 | 0.013 | 0.019 |
| scale-eater v.  generalist | 0.0075 | 0.0091 | 0.0045 | 0.0009 | 0.0104 | 0.0073 | 0.008 | 0.008 | 0.01 | 0.008 | 0.01 | 0.004 | 0.008 |
| molluscivore v.  generalist | 0.0073 | 0.0087 | 0.014 | 0.0195 | 0.0059 | 0.0069 | 0.0079 | 0.0019 | 0.001 | 0.005 | 0.007 | 0.0099 | 0.005 |
| scale-eater vs.  *C. laciniatus* | 0.014 | **0.001** | 0.015 | 0.019 | **0.002** | **0.0017** | **0.001** | 0.011 | **0.008** | 0.014 | **0.003** | **0.0017** | 0.012 |
| molluscivore v.  *C. laciniatus* | **0.0011** | 0.018 | **0.0018** | **0.0008** | 0.015 | 0.012 | 0.015 | **0.0004** | 0.012 | **0.0005** | 0.012 | 0.014 | **0.0002** |
| scale-eater v.  *C. bondi* | 0.019 | 0.024 | 0.016 | 0.022 | 0.018 | 0.015 | 0.016 | 0.014 | 0.018 | 0.016 | 0.017 | 0.012 | 0.015 |
| molluscivore v.  *C. bondi* | 0.017 | 0.022 | 0.013 | 0.024 | 0.012 | 0.013 | 0.015 | 0.013 | **0.009** | 0.015 | 0.012 | 0.016 | 0.014 |
