# Supplementary material for: Adaptive introgression from distant Caribbean islands contributed to the diversification of a microendemic adaptive radiation of trophic specialist pupfishes
Source: PLoS Genet. 2017 Aug 10;13(8):e1006919. doi: 10.1371/journal.pgen.1006919 (PMC5552031; doi:10.1371/journal.pgen.1006919)
Supplement: S5 Table — San Salvador Island generalist (A), San Salvador Island large-jawed scale-eater (L), San Salvador Island small-jawed scale-eater (S), San Salvador Island molluscivore (M), C. laciniatus from New Providence Island Bahamas (CUN), C. bondi from Dominican Republic (ETA), most recent common ancestor of Caribbean pupfish lineages (MRC). (DOCX) [file pgen.1006919.s029.docx]

**S5 Table. Summary of admixture events inferred by TREEMIX for the adaptive introgression regions assigned to the three alternative topologies.** San Salvador generalist (A), San Salvador large-jawed scale-eater (L), San Salvador small-jawed scale-eater (S), San Salvador molluscivore (M), *C laciniatus* from New Providence Island Bahamas (CUN), *C. bondi* from Dominican Republic (ETA), most recent common ancestor of Caribbean pupfish lineages (MRC).

| **Scaffold** | **Segment** | **F4** | **gene** | Admix events | CUN into L | CUN into M | ETA into L | ETA into M | Other |  |  |  |
| --- | --- | --- | --- | --- | --- | --- | --- | --- | --- | --- | --- | --- |
|  |  |  |  |  |  |  |  |  |  |  |  |  |
| KL652649.1 | 863668-873661 | 0.2536 | NA* | 3 |  |  |  |  | root M/CUN/PIG/G into S | MRC into A | MRC into PIG |  |
| KL652702.1 | 312277-322263 | 0.2461 | celf4 | 2 |  |  |  |  | M into L | L into S | S into L |  |
| KL652715.1 | 799363-809363 | -0.223 | pard3* | 2 |  |  |  |  | M into MRC | M into G |  |  |
| KL652867.1 | 545190-575190 | -0.28 | nbea | 3 |  |  |  |  | CUN into PIG | S into ETA | M into S |  |
| KL652983.1 | 269059-279054 | 0.2606 | **ski*** | 1 |  | X |  |  |  |  |  |  |
| KL653033.1 | 403145-413142 | -0.279 | NA | 4 |  |  |  |  | ETA into A | M into A | M into S | M into L |
| KL653171.1 | 362672-372487 | -0.255 | ltbp2 | 2 | X |  |  |  | M into A |  |  |  |
| KL653356.1 | 50344-70348 | 0.26 | srbd1 | 2 |  |  |  |  | PIG into S | root M,CUN,L into G |  |  |
| KL653356.1 | 70356-80348 | -0.267 | srbd1 | 2 | X |  |  |  | PIG into S |  |  |  |
| KL653906.1 | 10377-20368 | -0.228 | mcu | 2 |  |  |  |  | M into PIG | M into A |  |  |
| KL652964.1 | 411177-421153 | -0.275 | rbms3 | 3 |  |  |  |  | PIG into ETA | root L,S into PIG | root L,S into A |  |
